# Burden of Bacterial Antimicrobial Resistance in Libya, 1970–2024: A Systematic Meta-Analysis with Projections to 2050

**DOI:** 10.3390/antibiotics15010092

**Published:** 2026-01-16

**Authors:** Farag A. Bleiblo, Madiha W. El-Awamie, Nariman A. Elsharif, Muetaz M. Feetouri, Ibtihag S. Alogali, Abdelhafid A. Mohamed, Mahmoud A. Aloriby, Allaaeddin A. El Salabi, Tarek S. Bader, Souad A. Moftah, Omar S. Alqabbasi, Guma M. Abdeldaim, Eman M. Almajbry, Mohamed M. Khamid, Yousef M. Hasen, Yusra Layas, Shamsi S. Shamsi, Ali M. Milad, Abdulah D. Alamami, Ghaliah H. Elraid, Aziza S. Hamed, Aeshah A. Altajouri

**Affiliations:** 1 Department of Microbiology, Faculty of Science, University of Benghazi, Benghazi P.O. Box 1308, Libya; madiha.elawamie@uob.edu.ly (M.W.E.-A.); nariman.elsharif@uob.edu.ly (N.A.E.); eman.mohamed@uob.edu.ly (E.M.A.); 2 Department of Biomedical Sciences, Faculty of Pharmacy, University of Benghazi, Benghazi P.O. Box 1308, Libya; muetaz.feetouri@uob.edu.ly (M.M.F.); ibtihag.saleh@uob.edu.ly (I.S.A.); abdullah.alamami@uob.edu.ly (A.D.A.); ghaliah.hamed@uob.edu.ly (G.H.E.); 3 Department of Computer Science, School of Basic Sciences, Libyan Academy of Postgraduate Studies, Benghazi P.O. Box 1314, Libya; hafithmathe@lab.edu.ly; 4 Department of Pathology, Medical Center, Libyan International Medical University, Benghazi P.O. Box 84373, Libya; mahmoud.aloriby@limu.edu.ly; 5 Department of Public Health, Faculty of Medical Technology, University of Tripoli, Tripoli P.O. Box 82648, Libya; a_elsalabi@uot.edu.ly; 6 NAVCO Pharmaceuticals, Burlington, ON L7L 6B5, Canada; tarek@navcopharma.com; 7 Department of Physiology, Faculty of Medicine, University of Benghazi, Al-Marj Campus, Al-Marj P.O. Box 1308, Libya; souad.moftah@uob.edu.ly; 8 Department of Molecular Diagnostics, Faculty of Biomedical Sciences, University of Benghazi, Benghazi P.O. Box 1308, Libya; omar.algabbasi@uob.edu.ly; 9 Department of Medical Microbiology, Faculty of Medicine, University of Benghazi, Benghazi P.O. Box 1308, Libya; guma.abdeldaim@uob.edu.ly; 10 Department of Physiology, Faculty of Medicine, University of Benghazi, Benghazi P.O. Box 1308, Libya; mohamed.mahdi@uob.edu.ly; 11 Department of Pathology, Faculty of Dentistry, University of Zawia, Zawia P.O. Box 16418, Libya; y.hasen@zu.edu.ly (Y.M.H.); a.milad@zu.edu.ly (A.M.M.); 12 Department of Chemistry, Faculty of Science, University of Benghazi, Benghazi P.O. Box 1308, Libya; yusra.layas@uob.edu.ly; 13 Department of Medical Laboratory, Faculty of Medical Technology, Sebha University, Sebha P.O. Box 68, Libya; sha.saad@sebhau.edu.ly; 14 Laboratory of Microbiology, Al-Galaa Hospital, Benghazi P.O. Box 84373, Libya; alhkhatrani@yahoo.com; 15 Asaleem Medical Laboratory, Unit of Microbiology, Benghazi P.O. Box 84373, Libya; aishat@yahoo.com

**Keywords:** meta-analysis, PRISMA, Libya, antimicrobial resistance, global burden of disease, disability-adjusted life years, Gram-negative bacterial infections, tuberculosis, multidrug-resistant pathogens

## Abstract

**Background:** Libya, a conflict-affected North African country, has a fragile health system and poor surveillance, leaving it largely underrepresented in global estimates. Earlier Libyan reviews were descriptive, lacking breakpoint standardization, isolate-level pooling, or AMR-attributable mortality and DALY estimates. To our knowledge, this study represents the first comprehensive report that integrates phenotypic and genotypic data to estimate deaths and DALYs attributable to AMR-induced mortality and morbidity, describe spatiotemporal patterns, and model future trajectories. **Methods:** We performed a meta-analysis according to the PRISMA 2020 guideline of Libyan studies reporting phenotypic or genotypic resistance among clinical bacterial isolates (1970–2024), combined with microbiology records from hospitals and national surveillance systems (preregistered in PROSPERO ID: CRD420251066018). Susceptibility results were standardized to CLSI/EUCAST and deduplicated using WHO GLASS first-isolate rules. We used random-effects meta-regression to estimate pooled resistance, and the counterfactual approach of Global Burden of Disease (GBD) was applied to estimate AMR-attributable DALYs. Molecular data on resistance genes, sequence types, and tuberculosis mutations were systematically collected. **Results:** We included 62 eligible studies together with national and facility-level surveillance datasets, providing isolate-level susceptibility data for 31,439 clinical isolates from Libya. In 2024, we estimated 2183 deaths (95% UI 1752–2614) attributable to AMR, representing 9.7% (95% UI 7.8–11.6) of total deaths with a mortality rate of 15.2 per 100,000 (12.2–18.2). DALYs attributable to AMR increased from 14,628 (95% UI 11,702–17,554) in 1970 to 96,715 (95% UI 77,372–116,058). The highest pooled resistance involved carbapenem-resistant/MDR *A. baumannii*, third-generation cephalosporin- and fluoroquinolone-resistant Enterobacterales, and carbapenem-resistant *P. aeruginosa*. Molecular data showed widespread ESBLs, OXA-/NDM-type carbapenemases, plasmid-mediated colistin resistance, high-risk *E. coli* ST131 and *K. pneumoniae* ST147 lineages, and canonical drug-resistant *M. tuberculosis* mutations. **Conclusions:** Combined with global and regional evidence, our findings suggest a high and increasing burden of AMR in Libya. These findings emphasize the need for rapid expansion of data collection systems, GLASS-aligned surveillance, diagnostic capacities, and infection control measures.

## 1. Introduction

Antimicrobial Resistance (AMR) is an emerging global health threat with profound consequences for under-resourced nations with fragile health systems. The WHO has identified AMR as one of the top ten current public health challenges. Recently, it has been estimated that 1.27 million deaths occur annually because of drug-resistant infections and an additional 4.95 million deaths are also indirectly associated with drug-resistant pathogens globally [1,2]. International initiatives, including the WHO Global Action Plan and One Health frameworks, promote coordinated action across human, animal, and environmental sectors [3,4], but implementation remains inconsistent, particularly in low-income, politically unstable countries where poor governance, weak surveillance, and multisectoral coordination issues restrain progress [5].

Poor surveillance systems, weak regulatory measures, and regional conflicts within the Eastern Mediterranean/North African regions (EMENA) further exacerbate the AMR challenges. Recent WHO-EMRO reports have shown that resistance patterns are rising, particularly among *Acinetobacter baumannii*, *Pseudomonas. aeruginosa*, and carbapenem-resistant Enterobacterales (CRE) [6,7]. In Libya, long-term conflict since 2011 has further aggravated this situation, resulting in the destruction of health facilities, the impairment of antimicrobial supply chains, and a deterioration of the infection prevention and control systems [8]. Legislation in the human and veterinary sectors in the country is weak, and non-prescription antibiotic sales are common [9,10]. Low-quality medications are common due to weak regulation, while poor microbiology diagnostics frequently result in the empirical use of broad-spectrum antibiotics [11].

Despite the rising AMR threat, Libya lacks a comprehensive nationwide surveillance system, and data are currently fragmented both geographically and temporally [12]. Most studies are limited to urban health facilities, use diverse methodologies, and provide limited information on the burden of AMR in Libya [13,14]. Phenotypic antimicrobial susceptibility testing still prevails, with limited use of molecular diagnostics. Therefore, there is limited availability of Libyan clinical isolates archived in international genomic databases, including GenBank and the European Nucleotide Archive [15], which restricts our knowledge of resistance mechanisms, genetic spread, and transmission. The integration of molecular epidemiology in AMR surveillance may help better understand resistance patterns, assist in tracking lineages, and inform national policy [16].

Two reports at the national level have previously reviewed AMR in Libya. Ghenghesh and collogues (1970–2011) reported sparse and methodologically heterogeneous data, with high resistance in enteric bacteria, methicillin-resistant *Staphylococcus aureus* (MRSA), and multidrug-resistant *M. tuberculosis*. The authors emphasized the absence of structured surveillance and standardized AMR reporting [17]. Atia et al. subsequently conducted a systematic literature review of 43 studies published between 2002 and 2021, encompassing 18,160 antimicrobial susceptibility tests and observed high levels of resistance to commonly used antibiotics in common clinical syndromes. The authors thus call for a national AMR action plan to be developed and for improvement in laboratory capacity [18].

Although these reviews highlighted that AMR is a serious and rising problem in Libya, they were restricted to the published literature and largely descriptive. In addition, they did not standardize susceptibility data to CLSI/EUCAST or WHO-GLASS definitions, and combined them with routine hospital or national surveillance datasets to produce estimates of deaths or DALYs [19,20,21,22]. Consequently, Libya remains under-represented in global AMR burden estimates, and no national analysis has yet linked phenotypic resistance, molecular mechanisms, and health loss over time.

Building on these Libyan studies, we used surveillance data and GBD methods to model the national burden and time trends in priority bacterial AMR from 1 January 1970 through 31 December 2024 in Libya. It investigates molecular epidemiology (prevalent resistance genes and, where provided, phenotype–genotype concordance), maps spatiotemporal patterns, and projects outcomes to 2050 following the reference and intervention scenarios as well as uncertainty intervals. We used an integrated approach of systematic review, meta-analysis, mathematical modelling, available whole-genome sequencing (WGS) evidence, and scenario-based forecasting. Reporting follows PRISMA 2020, and susceptibility data are interpreted using CLSI and EUCAST standards [17,18,19,20]. Forecasting models incorporated varying intervention scenarios to generate policy-relevant estimates for use in decision-making on AMR control within the context of Libya’s existing health system.

## 2. Materials and Methods

### 2.1. Study Design

In this systematic analysis, we integrated the literature estimates, molecular surveillance data, geospatial predictions, and predictive models from the available sources of bacterial AMR. The present systematic review and meta-analysis were conducted as per PRISMA (2020) guidelines and registered in PROSPERO (CRD420251066018). To compare the model risk framework to the risk approach, justified based on Libya’s fragile and conflict-affected health system, the GBD was adopted [6,23]. Results reporting was performed following PRISMA 2020 and GATHER guidelines; surveillance datasets were gathered using WHO first-isolate de-duplication and standardized to recent CLSI/EUCAST breakpoints. The analysis covered period between 01 January 1970 and 31 December 2024, further subdivided by the following phases to represent temporal changes in AMR burden: (1) pre-conflict baseline development (1970–2010); (2) acute healthcare disruption (2011–2015); (3) fragmented governance (2016–2020); and (4) partial system reconstitution (2024). The burden of resistance was forecasted to 2050 at 5-year intervals for future planning purposes.

### 2.2. Data Sources

We used a multi-source strategy for the heterogeneous and fragmented assessment of AMR surveillance in Libya. The strategy was employed to consider spatial, institutional, temporal, and surveillance differences to make our dataset representative and analytically robust. The aim was to derive a standardized datasets that contribute to better epidemiological and longitudinal trend analyses. A summary of data sources is shown in Table S1. Data were collected from five main sources: national and subnational surveillance data, including hospital and clinical microbiology laboratory (private and public) records of AMR (including, Benghazi Medical Center, Al-Jalaa Hospital, Al-Kweifiya Hospital, Sabha Medical Hospital, and tertiary hospitals in Tripoli); published peer-reviewed literature or systematic reviews; reports on antimicrobial drug usage/sales (including aggregated antibiotic dispensing data from a small convenience sample of private community pharmacies in Benghazi and other Libyan cities where available); and mortality data/hospital discharge datasets. A summary matrix of data sources, coverage, and extraction fields is presented in Table S1. These sources were assessed for methodological integrity, extent of temporal and spatial coverage, as well as availability of metadata. Inclusion criteria were quantitative AST results accompanied by protocols, interpretive breakpoints, and isolate metadata. Data were excluded if they lacked clear methodological details and were collected through non-standardized AST methods.

To maintain consistency with the analysis structure, we established a hierarchical data structure (shown in Table S1). This structure defined for each dataset the origin, level of surveillance and type of AMR metric and allowed for a stratified weighting and calibration in the modelling. This model is also designed to ensure methodological consistency across the four targeted historical phases of development in Libya’s health systems trajectory. The resulting databases reflect clinical microbiology trends and assess health system factors affecting AMR emergence and spread.

### 2.3. Systematic Review and Meta-Analysis

**Figure 1:**
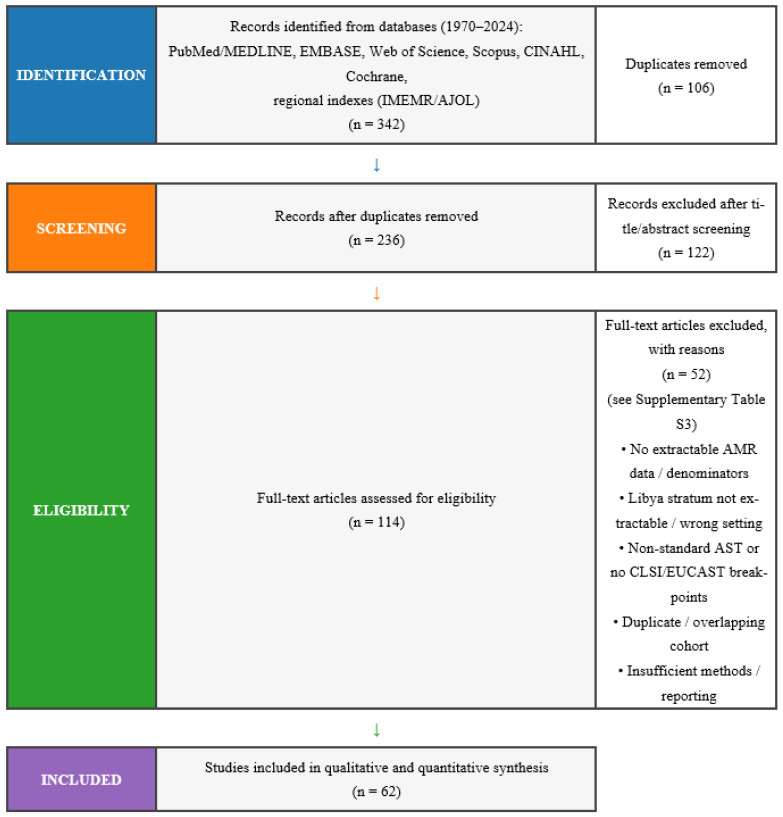
**Flowchart of PRISMA-2020 study selection for the systematic review of antimicrobial resistance in Libya (1970–2024).** Records were identified through database searches (PubMed/MEDLINE, EMBASE, Web of Science, Scopus, CINAHL, Cochrane Library and IMEMR/AJOL) (n = 342); duplicates removed (n = 106); records screened at title/abstract level (n = 236); records excluded (n = 122); full-text articles assessed for eligibility (n = 114; of those n = 52 further excluded, see Supplementary Table S1); studies included in qualitative and quantitative synthesis (n = 62).

We conducted this review according to PRISMA 2020, which was preregistered under (PROSPERO ID: CRD420251066018). A completed PRISMA 2020 checklist is provided in the Supplementary Materials. Combined geospatial terms (‘Libya’, ‘Tripoli’, ‘Benghazi’, ‘Cyrenaica’, ‘Fezzan’, and ‘Tripolitania’) with resistance terms including organism-drug and resistance-mechanism pairs (antimicrobial resist or antibiotic resist) were structured in a Boolean search. Complete search strings and export logs are shown in Table S2. We searched PubMed/MEDLINE, EMBASE, Web of Science Core Collection, Scopus, and Cumulative Index to Nursing and Allied Health Literature (CINAHL), as well as Global Index Medicus: WHO regional databases using the search tool such as Index Medicus for the Eastern Mediterranean Region (IMEMR) and African Journals Online (AJOL) from 1 January 1970 to 31 December 2024 with an English and Arabic language restriction. The number of raw hits and de-duplication counts are listed in Table S2. We found a total of 342 records across databases: After removing 106 duplicates, 236 titles/abstracts were screened, which then excluded non-relevant records (122). Full texts of 114 articles were assessed, further excluding articles for various reasons; in most cases, the exclusion was related to outcome reporting. A pool of 62 studies entered the qualitative–quantitative analysis. Complete search strategies (database, platform, dates, limits, and full Boolean strings) are presented in Table S2. The PRISMA 2020 flow diagram (Figure 1) provides an overview of the study selection process.

Studies on humans from Libya (of any age, inpatients or outpatients) were eligible if the clinical specimens identified a pathogenic bacterial isolate; multicountry studies were included if a Libyan stratum was discernible. The main outcomes were the frequency of non-susceptible/resistant isolates, stratified by organism–drug at concordant CLSI/EUCAST breakpoints. The secondary outcomes were the subgroup and trend analyses planned a priori based on time phase, setting, sample type, age group, AST technique, and bias-risk category. The present study design was surveillance, laboratory-based prevalence, examining cohorts/clinical studies with extractable denominators (n tested) and numerators (n resistant/non-susceptible).

For eligibility criteria, we included Libya-only studies (and those Libyan-strata extractable) and with AST by disc diffusion, broth microdilution, automated systems, or gradient strips according to the CLSI/EUCAST, with routine quality control. We excluded non-standard AST studies and studies with no breakpoints or sample dates outside our preferred range when raw MIC/zone data were not available to allow for reclassification. We also excluded data with very small series of a given organism drug pair (n tested <30) from quantitative synthesis, inaccurate geography and time window sources that came from animal/environmental isolates testing, outbreak reports written based on only one phenotype, and reviews/editorials/abstracts that had no extractable data. Examples for reasons to exclude full-text literature are given in Table S3.

Records were de-duplicated and screened by title/abstract and full text by two independent reviewers; any disagreements were resolved through consensus or third-party adjudication. The level of inter-rater agreement was substantial (Cohen’s κ = 0.87). Two authors independently extracted identifiers (first author, year, region/setting); design; enrolment period and recruitment strategies; population criteria; specimen collection types; organisms and antimicrobials studied by the AST method, for which a breakpoint was obtained; AST method used; breakpoint standard/version (indicating Breakpoint standard CLSI/EUCAST); quality-control reporting; and numerators/denominators for each organism–drug pair. Denominators were derived from text/tables/figures. Non-susceptibility was considered intermediate plus resistant unless raw MIC/zone data permitted reclassification to contemporary breakpoints.

Prevalence studies were assessed using the Joanna Briggs Institute (JBI) checklist (AMR-adapted domains). The quality of evidence for observational comparative designs was also evaluated by ROBINS-I. The domains for compatibility with the AST, reporting of the breakpoint version and quality control reporting of AST were regarded as important. Study-level risk was predefined (Low/Moderate/High) with justification as indicated in Table S4.

All eligible studies were included in primary analyses, and sensitivity analyses excluded high-risk studies. Risk-of-bias category and focused critical domains were used as moderators in meta-regression and subgroup analyses. Leave-one-out influence diagnostics were stratified by risk of bias. Both small-study effects and reporting bias are discussed in the Section 2.4.

We also searched ClinicalTrials.gov and WHO ICTRP for continuing or unpublished trials of AMR in Libya. Records from registries were sifted using identical inclusion criteria. Data (including extractable AST numerators and denominators for posted results) were considered; other data were described narratively and compared to bibliographic sources. This systematic review and meta-analysis were conducted and reported in accordance with PRISMA 2020; a completed PRISMA checklist and flow diagram are provided in S1 Checklist and Figure 1. Full texts assessed for eligibility, and studies included full-text exclusions are listed in Supplementary Table S3.

### 2.4. Statistical and Computational Analysis

#### 2.4.1. Integration and Management of Data

We developed a relational database to combine and harmonize all the eligible datasets. For inference and data harmonization, we applied a relational database based on an instance of PostgreSQL (v14.5) coupled with an extension for geospatial and genomic data attributes (e.g., PostGIS for geospatial features). Data ingestion was implemented with a reusable ETL (Extract, Transform, Load) pipeline written in Python’s pandas (v2.0.1) and SQLAlchemy (v2.0.0). The resulting schema for each transformation step was validated for referential integrity and validation constraints. The schema allowed for direct or hierarchical linking of healthcare facility property and patient- and isolate-related characteristics to the AMR phenotyping test. Data records were consistent due to foreign key constraints and index optimization of queries. This schema allows for rapid stratification both according to location and clinical details, which was exploited to provide population-level summaries as well as to examine resistance profiles. Software version, the main parameters, and quality control diagnostics (heterogeneity metrics, influence, and funnel eligibility) are presented in Table S5. Complete numerical meta-analytic results (k, n, pooled prevalence with 95% CI, τ^2^, I^2^, Q with *p*-values, and 95% prediction intervals where k ≥ 3) are shown in Table S7. The genomic variables were extracted from published data; any new sequencing information was not generated or analyzed. All analysis scripts were version-controlled (Python/R) with deterministic random seeds, and a data dictionary and ETL audit trail are provided in Tables S1 and S2.

#### 2.4.2. Handling Overlapping and Duplicate Data

A hierarchical structural analysis was employed to eliminate redundant and duplicated data. In the case of multiple published papers with overlapping data, the publication that provided the most informative report was included, considering publication size and completeness of primary or long-term endpoints. Original articles were included if they contained data not reported elsewhere in another article that was included. For isolate-level surveillance tabulations, we applied GLASS-concordant de-duplication (i.e., the first isolate for each patient, pathogen, and specimen type within a surveillance period; stratified outputs were also de-duplicated within GLASS strata). If a study contributed multiple non-independent effect sizes (e.g., several wards/specimen types), we selected the most comparable stratum for the primary analysis and used small-sample-corrected cluster-robust variance estimation in sensitivity checks.

#### 2.4.3. Processing of Spatiotemporal Data

We used geostatistical downscaling methods (space–time smoothing) to process inconsistent and limited AMR surveillance data that covered scattered regions in Libya. We applied the kernel density estimation (KDE) using an adaptive bandwidth and cross-validated for space–time tuning. KDE was performed using a Gaussian kernel with an adaptive k-nearest-neighbour bandwidth (default k = 50) by rolling-origin cross-validation, and edge-corrected at national borders. This technique allowed for the optimization of bandwidth choice against the trade-off between precision and spatial coverage, particularly in areas with low reporting. The spatiotemporal KDE reads are shown in Supplementary Equation (S1). To account for sparsity, we applied a Bayesian hierarchical model with the INLA framework. Model-based spatial random effects and temporal autocorrelation structures were added to this model to stabilize prevalence estimates through space over time. The log-prevalence model is presented in Supplementary Equation (S2). Because reporting was geographically clustered (“patchy”), the INLA spatiotemporal model was used to strength across neighbouring regions and adjacent years via spatial random effects and temporal autocorrelation (partial pooling). This stabilizes estimates in sparsely sampled areas while preserving wider uncertainty where data are limited. Therefore, regions with low reporting are not treated as equally well-observed as high-coverage urban settings.

#### 2.4.4. Methodology of Missing Data

To assess the risk of structural missingness, we distinguished between Missing at Random (MAR) and Missing Not at Random (MNAR) mechanisms. We imputed MAR covariates by multiple imputation of chained equation (MICE) to obtain 50 datasets with 200 iterations. We constructed an imputation model with 47 covariates (e.g., conflict intensity, access to healthcare services or development indices) to further increase precision and minimize bias. In the case of MNAR, if missingness was clustered at the peak of conflict or institution closure, we used a pattern-mixture model. Sensitivity parameter values were obtained from Libyan clinical microbiologists and AMR surveillance experts. The MNAR pattern-mixture sub-model appears in Supplementary Equation (S3). This approach ensured analytical robustness during periods of regular data censure and disrupted reporting. To evaluate the possibility that rural and conflict-affected locations are systematically under-captured, we treated conflict intensity and service access as predictors of missingness and incorporated them into the missing-data model. We report uncertainty intervals for all key estimates and interpret findings considering these coverage limitations.

#### 2.4.5. Sensitivity Analysis

We performed sensitivity analyses to evaluate robustness to (i) conflict-related structural missingness and (ii) geographic under-coverage. First, we refit the core models, excluding peak-disruption years (conflict period), to test whether trend direction and key estimates were driven by those intervals. Second, we performed MNAR sensitivity analyses using a delta-adjustment/pattern-mixture approach by varying the MNAR sensitivity parameter across plausible ranges to assess tipping-point behaviour. Third, we performed a coverage sensitivity analysis by down-weighting high-volume urban hospital series and/or applying population-based re-weighting to evaluate the influence of clustered reporting on national estimates.

### 2.5. Geospatial and Regional Analytical Methods

#### Geographic Stratification

Libya was divided into seven pre-established epidemiological units: Tripolitania, Cyrenaica, Fezzan, Coastal Strip, Sirte Basin, Southern Desert, and Central Highlands. The dynamics of AMR were studied at four levels (national, regional, district, and facility). We applied a spatial analytic triad approach to map the clustering and heterogeneity of AMR prevalence. Global spatial autocorrelation was explored using Moran’s I and Geary’s C statistics after adapting the spatial weights. Hotspot analysis was used to identify hot and cold spots using the Getis–Ord Gi measure. Geographically weighted regression (GWR) was used to explore geographically heterogenous associations between AMR and health system or demographic factors. The GWR specification is shown in Supplementary Equation (S4). This model enabled locally adaptive regression, which exposed context-specific drivers of resistance throughout Libya.

To measure the effects of healthcare access on AMR distribution, we used an enhanced two-step floating catchment area (E2SFCA). This model combines distance (physical–Euclidean and road networks), capacity (number of beds, diagnoses, staff positions), as well as disruption from conflict on events. Availability was modelled using WorldPop gridded population estimates for high-resolution equity analysis. Adjusted accessibility scores (A-i) were then included as covariates in the GWR models to adjust for the structural inequality of healthcare accessibility. By incorporating both the spatial closeness and system functionality, we were able to better interpret AMR gradients at a regional level.

### 2.6. Burden Estimation

To provide analytical outcomes, our quantitative analysis was focused on resistance prevalence per WHO-prioritized pathogen–drug combinations. Secondary analyses included the addition of temporal trends, stratified estimates by clinical setting and phenotype-genotype concordance measures. All resistance classifications were retro-adjusted to modern interpretative criteria (CLSI/EUCAST) for comparison with current thresholds of resistance likelihood. Study-level proportions were logit-transformed, pooled, and back-transformed via the inverse logit to produce overall combined estimates of resistance prevalence. A restricted maximum likelihood (REML) random-effects model was used in all instances of analysis to produce stable effect estimates in the presence of inter-study heterogeneity. Between-study variance (τ^2^) was estimated by REML; we report τ^2^, I^2^, and Cochran’s Q with *p*-value. Prediction intervals were also calculated where applicable to allow for anticipated heterogeneity in future surveillance studies. Possible small-study effects were assessed by visual inspection of funnel plots and Peters’ test for proportion outcomes. Influential studies were identified by Cook’s distance and DFBETAS, with influence diagnostics used to inform leave-one-out sensitivity analyses. Further sensitivity analyses were conducted by limiting to studies deemed to be at low risk of bias, removing data extracted from AST procedures that were not standardized and exploring the influence of different interpretative standards (CLSI versus EUCAST). Inputs, assumptions, transformations (including breakpoint harmonization and first-isolate de-duplication), model specifications, and data/code access details are documented in a GATHER-transparency inventory in the Supplement Table S8.

The counterfactual risk modelling using a GRAM-aligned framework was used to estimate the burden of health effects associated with AMR in Libya: DALYs were calculated as YLL + YLD using GBD-consistent disability weights and standard life expectancy. Three types of comparative case series were developed: (1) resistant versus susceptible infection of the same pathogen; (2) infected case versus non-infected baseline population; and (3) resistant infection versus infection with an alternative pathogen lacking the resistance profile. Attributable mortality was derived from pathogen-specific and resistance-specific mortality counts, stratified by age, sex, and comorbidity, and adjusted using relative risk estimates for susceptible and resistant infections. Attributable mortality was calculated using Supplementary Equation (S5). In addition to estimates of mortality, levels for Years of Life Lost (YLL) and Years Lived with Disability (YLD) were produced for DALY calculations. YLLs were calculated using age-specific life expectancy from Libya-specific World Bank tables, and YLDs were multiplied by the disability weights equivalent to those of the GBD approach. This methodology enabled the estimation of population-adjusted burden estimates, accounting for differences in age and sex among Libya’s decentralized health system, providing varying levels of access to care.

### 2.7. Forecasting and Scenario-Based Modelling

To project the burden of AMR in Libya through 2050, we used a multi-model approach. In this method, we combined four complementary modelling approaches to investigate temporal dynamics, causality, and complexity of pathogen transmission. Estimates were based on accumulated surveillance data and published molecular reports. We also used the Prophet time-series decomposition with additional Fourier terms to investigate seasonality and employed priors to reflect a changepoint due to disruption from the Libyan conflict. Furthermore, to predict the influence of antimicrobial consumption, infection control interventions and health system performance on resistance profiles, we employed a Bayesian structural equation model (BSEM). Third, machine learning algorithms, including XGBoost and LSTM neural networks, were used to investigate non-linear relationships and lags that were not captured previously by conventional approaches. Lastly, compartmental mechanisms models, including terms of acquisition of resistance, horizontal gene transfer, and intervention effects, were used to estimate host and population transmission. Point estimates from models were combined by Bayesian model averaging according to out-of-sample predictive value across pre-specified stratified validation subsets. The aim of this ensemble-based structure was to obtain more flexible uncertainty quantification and prediction performance.

A structured Delphi process of national and international AMR experts was employed to develop four alternative scenarios for how the policy response could shape future AMR burden in Libya. Scenario 1 was the reference case (predefined as the continuation of ongoing antimicrobial use, diagnostic coverage, and the health system’s capacity). For Scenario 2 (WHO-GAP), the national adoption of WHO-GAP on AMR, with improved stewardship, surveillance and infection prevention, was simulated. In Scenario 3, we simulated systemic failure to be comparable to health-systems collapse under the conflict environment. Regional cooperation in Scenario 4 was modelled and enhanced through greater cross-border coordination, harmonized AMR surveillance, and coalescence among North African/Mediterranean nations. Confidence in the predicted estimates was quantified with the stochastic sensitivity analysis, which sampled 10,000 parameter sets via Latin hypercube sampling (LHS) and maintained rank correlations between key input parameters. The estimates were reported as posterior medians and 95% uncertainty intervals, including the parameter and between-model uncertainties.

### 2.8. Molecular Data Abstraction

In the study, we did not produce any novel sequencing data. Molecular epidemiology data (i.e., resistance genes, carbapenemase families, plasmid replicons, sequence types/lineages, SNP-cluster results) were based on the available peer-reviewed Libyan publications and/or national/regional surveillance reports of studies that qualified for inclusion in our systematic review [24,25,26,27,28,29,30,31,32,33,34,35,36,37,38,39,40,41,42,43,44,45,46]. For each report included, we extracted the organism name, year/region of isolate collection, setting where the study was conducted (e.g., hospital), method (PCR, MLST or WGS), gene/allele names (e.g., *bla*NDM-1 and *bla*OXA-23), plasmid replicon type(s) of interest to this review, and sequence type or lineage of the relevant organisms for that description, as well as pairwise single nucleotide polymorphism distances and and-cluster definitions. Data quality items (platform, read metrics, assembly/QC thresholds, resistance gene callers) were recorded if reported. We also did not perform any re-assessment of raw reads or assemblies. Reported frequencies are based on these merged data and expressed as sequenced/typed isolates per species in the respective sources.

### 2.9. Integration of Laboratory and Surveillance Data

A hierarchical resistance ontology was developed to combine phenotypic data with the published genomic surveillance data, thereby harmonizing these two sources in an integrated analysis framework. The ontology was split into four hierarchical levels: Level 1, phenotypic resistance profiles only; Level 2, inclusion of known PCR or targeted molecular detected resistance genes; Level 3, placing reported genes in context with genetic location, such as mobile genetic elements, chromosomal context, and insertion sequences; and finally, Level 4, genome-wide determinants (as described from published WGS analyses). This hierarchical structure allowed for the retroactive harmonization of the 1970–2024 dataset as well as longitudinal molecular epidemiology. Uncertainty modelling at all levels of the hierarchy, particularly when molecular resolution was low, allowed for reliable combination across time points and datasets.

We characterized an isolate-level AST record as a unique organism–patient–episode entry with at least one antimicrobial susceptibility test result. For the descriptive subset described in Table 1, numbers were adjusted according to the WHO GLASS rule: first isolate per patient, per pathogen, per specimen type, per surveillance period (12 months); where stratified outputs were produced, de-duplication was performed within GLASS strata (age, sex, infection origin). All burden estimates (AMR-attributable deaths/DALYs) are model-derived and not a function of a specific raw isolate total.

### 2.10. Microbiological and Molecular Framework

The pathogen priority was ranked by species against the 2024 WHO Bacterial Priority Pathogens List (BPPL), which placed organisms in critical, high, or medium priority depending upon the severity of clinical resistance, level of resistance, availability of treatment and public health impact. To frame this categorization for our country setting of Libya, we generated a composite map from early surveillance with data from the Libyan National Centre for Disease Control (NCDC) and other published data on international AMR platforms. This involved regional antibiograms and resistance epidemiology, which were taken to characterize region-specific pathogen prevalence and microbiological treatment. The hierarchical matrix that connects global priority rankings with Libyan epidemiology and resistance factors is shown in Table S6. Sources of data were national summary NCDC reports, hospital antibiograms, and peer-reviewed articles.

We standardized case definitions for AMR infections according to international diagnostic and surveillance criteria and justified them for low-resource settings. Healthcare-associated infections were defined using CDC/NHSN definitions, and community-acquired infections were defined by syndrome-specific algorithms promulgated by the International Clinical Definitions Consortium. Given inter-facility variability in diagnostic capacity across Libyan health institutions, a tiered classification system was applied to enhance diagnostic validity. These consisted of (1) confirmed cases, where there is a clinically compatible syndrome and evidence of infection supported in the laboratory (using validated tests) by either resistance detected with standardized susceptibility testing or a molecular microbiological method; (2) probable cases, presenting with clinical features but without positive culture/antigen detection from non-reference microbiology laboratories only; and (3) suspected cases, presenting with compatible syndromes but lacking laboratory confirmation. For all cases, a matrix of space (community vs. hospital), specimen type for the isolate, and for any others obtained from the same patient during the 6-month period since the earliest date of culture positivity, prior antimicrobial treatment, and patient demographics was constructed.

We included routine laboratory data with quality assurance standards to provide comparability and stratified. Historical datasets (1970–2000) consisted of biochemically identified isolates and were standardized to disc diffusion assays. Contemporary data (2001–2024) were derived from laboratory reports that followed validated methods, including E-test or disc diffusion tests. Automated techniques for high-throughput diagnostics, such as BD Phoenix and VITEK-2, were also included when used according to guidelines. Broth microdilution assays, if available, were considered the gold standard.

### 2.11. Antimicrobial Susceptibility Interpretation

AST data were first normalized to address inter-laboratory variations, changing breakpoint standards and the type of clinical specimen sourced for the isolates. Back-mapping of historical susceptibility values was performed, where possible, by relating original zone diameter or minimal inhibitory concentration (MIC) breakpoints to contemporary interpretive criteria (e.g., CLSI and EUCAST guidelines [20,21].

#### Quality Assessments and Methodological Validation

We subjected the microbiological data used in the analysis to quality assessment. Our analytic framework focused on the quality of data sources, quality control (e.g., simultaneous testing against reference strains), and external validation through participation in external quality assessment (EQA) schemes were recorded. Phenotype–genotype concordance was summarized only when reported in the extracted publications. Data sources were also evaluated in terms of test performance. A quality score composite of each data extract was obtained by a validated algorithm on a 0–100 scale. These scores were then used as analytic weights in both the model fitting and sensitivity estimation stages. Data sources with a score of less than 40 points were excluded from the main analysis. However, we included them in the sensitivity analysis to test their impact on model consistency and bias estimation.

### 2.12. Ethical Approval

All aspects of the study were conducted in accordance with international ethical standards. Ethical approvals were obtained from the Government of Libya, Ministry of Health, Department of Research and Training (Approval No. MoH-Libya-AMR-1970-2024NBAMR-03-2025). The research adhered to the Declaration of Helsinki and CIOMS International Ethical Guidelines for biomedical research involving human patients. No new patient recruitment, specimen collection, or sequencing was undertaken in this study; all analyses only used de-identified surveillance, microbiology, and pharmacy datasets and published reports. For any data mining that was conducted on the existing surveillance, hospital, private laboratory, and community pharmacy data, agreements for the use of data were made with the healthcare facilities, private laboratories, and pharmacies involved to adhere to national laws and global frameworks concerning data protection if there were retrospective components. To ensure participant anonymity, data were de-identified and stored in encrypted, access-controlled databases. Governance, secondary-use approval, and custodianship oversight mechanisms were established to maintain the integrity, security, and ethical standards of the research.

## 3. Results

### 3.1. Study Selection and Characteristics of Included Studies

We identified 342 reports from database searches. Prior to screening, 106 duplicates were removed, resulting in 236 records for title/abstract screening, and 122 were excluded. Following full-text assessment, of the 114 full texts remaining, 62 studies were eligible for inclusion in this review. The results of all 62 that provided data used in the quantitative analysis are presented in Figure 1 (PRISMA 2020) and Table S3 (excluded with reasons). For every pooled meta-analytic estimate, we report k (studies), I^2^, τ^2^ (estimator: REML), Q(df, p), and the pooled proportion with its 95% CI; 95% prediction intervals are provided where eligible (k ≥ 3). For broader trend and modelling analyses, we integrated these with national and international surveillance sources, resulting in a national isolate-level dataset. The descriptive subset used in Table 1 comprises 31,439 de-duplicated isolate-level AST records.

#### 3.1.1. Data Properties and Geographic Coverage

**Figure 2:**
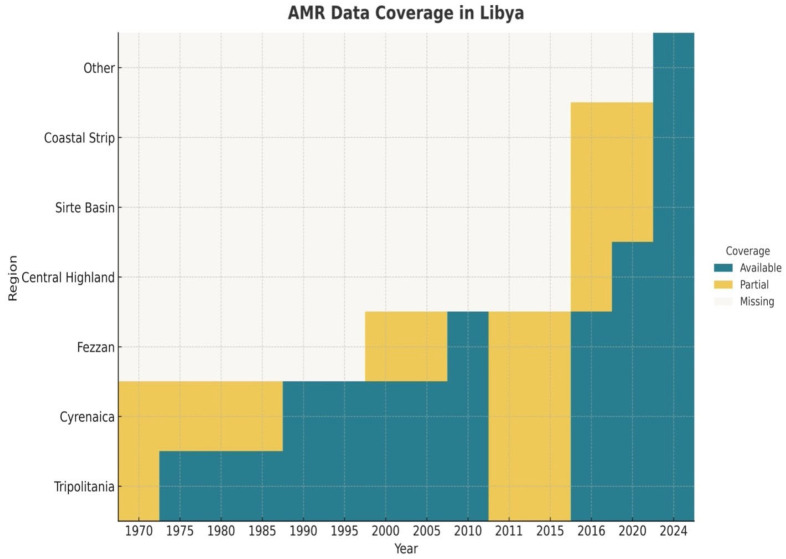
**AMR data coverage by region and year, Libya, 1970–2024.** Heat map showing AMR surveillance coverage by region and calendar year, with the following coding: Available, Partial, or Missing. The coverage commenced in Tripolitania/Cyrenaica, was interrupted during the 2011–2015 conflict, and increased to the close-to-national level by 2021–2024. The Other category subsumes smaller entities and special-purpose facilities. Definitions: Available—continuous annual isolate- specific AST with extractable numerators/denominators; Partial—intermittent/subnational/heterogeneous AST; Missing—no extractable AST. Abbreviations: AMR—antimicrobial resistance; AST—antimicrobial susceptibility testing; GLASS—WHO Global Antimicrobial Resistance Surveillance System; EMRO-AMR—WHO Eastern Mediterranean Regional Office AMR network; NCDC—National Center for Disease Control (Libya).

In the early period (1970–1995), we found that data coverage was limited and heterogeneous across jurisdictions, but we observed a clear trend for improving coverage from 2005 onward. However, this trend was almost nationally representative around the year 2024. The spatial–temporal coverage by region and year is shown in Figure 2. A cross-sectional summary of regional and facility-tier representation for 2024 is provided in Table 2.

#### 3.1.2. Temporal Trends During Conflict

**Figure 3:**
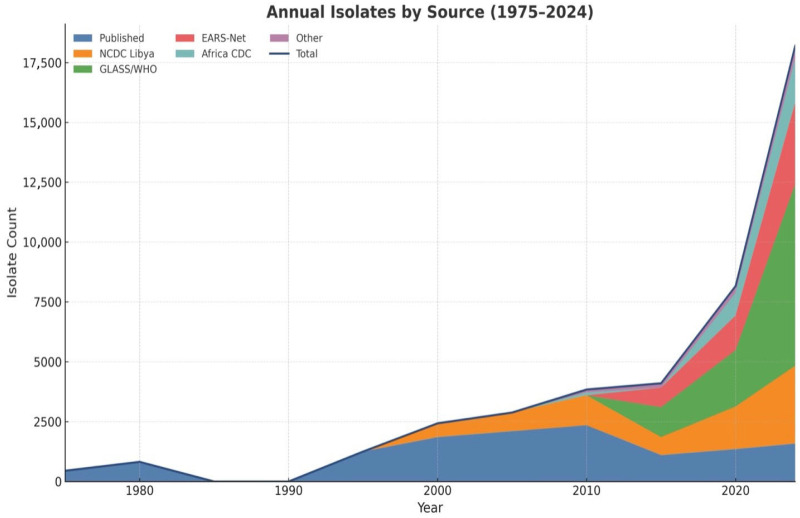
**Annual AMR isolates by data source, Libya, 1970–2024 (plot begins in 1975; no extractable AST records 1970–1974).** Stacked area chart of annual number of bacterial isolates with available AST by year and source (journal articles, NCDC-Libya, GLASS/WHO, Africa CDC, and all other sources); overlaid line represents total per year. Coverage was dominated by publications/NCDC pre-2010 and during the conflict period (2011–2015) reporting dropped, followed by a marked increase from 2020 to 2024 that likely reflects onboarding to GLASS/WHO and Africa CDC. (Note: EARS-Net is an EU/EEA comparator, and we did not use it as Libya source data.) Abbreviations: AMR—antimicrobial resistance; AST—antimicrobial susceptibility testing; NCDC—National Center for Disease Control (Libya); GLASS/WHO—Global Antimicrobial Resistance Surveillance System/World Health Organization; EARS-Net—European Antimicrobial Resistance Surveillance Network; Africa CDC—Africa Center for Disease Control and Prevention.

During the period of the Libyan conflict 2011–2015, there was a pronounced disruption in the pattern of temporal trends regarding both data availability and reporting completeness, coincident with a substantial decrease in reported isolates as well as laboratory throughputs. The decrease was also concurrent with the collapse of healthcare institutions and diagnostic facilities. However, there was a recovery tendency observed in the post-conflict phase, driven by increasing international collaboration and slowly establishing of organized surveillance systems (the development of GLASS, EMRO-AMR, and Africa CDC initiatives). Contributions from NCDC/Libya and international surveillance partners after 2016 reflect improved diagnostic harmonization through the standardization process as well as interagency data sharing. Annual isolate counts by data source are summarized in Figure 3.

#### 3.1.3. Regional and Health System Tier Representation

The relative contribution of each region by population and healthcare capacity was evaluated through a formal representativeness analysis. While most isolates were identified in urban tertiary care centres—i.e., 65.1% (20,467/31,439) in 2024 (regionally weighted share of isolates; Table 2) and 63.7% (20,026/31,439) between 1970 and 2024—rural/primary healthcare sites remained largely underrepresented. The respective proportions of isolates from secondary and primary care were 26.5% (8331/31,439) and 8.4% (2641/31,439) in 2024 (Table 2). This pattern could be due to diagnostic capacity differences and underlying case-mix differences that may impact the observed levels of resistance.

#### 3.1.4. Methodological Standardization over Time

Prior to 2010, studies were heterogeneous for methodologies, isolate identification and AST in particular. Following CLSI and EUCAST guidelines was an important approach for standardizing at the Laboratory level [19,20,21]. According to these standards, the percentage of participating laboratories that met international standards increased from 23.4% in 2010 to 76.8% in 2022. This shift in laboratory practices was the basis for increased comparability and interpretability between new data, and provided more robust longitudinal trend analyses beginning in 2010.

#### 3.1.5. Meta-Analytic Synthesis Reporting (Heterogeneity)

The variability between studies (τ^2^) was estimated using restricted maximum likelihood (REML). Heterogeneity is reported and presented for each individual pooled estimate in the summary table as Q (df, p), I^2^, τ^2^; and, since k ≥ 3, a 95% prediction interval is shown to describe the expected range across settings (see Table S7).

### 3.2. National Burden of AMR (1970–2024)

By a counterfactual modelling approach, we estimated that AMR infections were responsible for 2183 deaths (95% UI: 1752–2614) in Libya in 2024 and accounted for 9.7% (95% UI: 7.8–11.6%) of total deaths. Of this burden, 187 DR-TB deaths (95% UI: 149–225) accounted for 8.6% of all AMR-attributable deaths in 2024. By using the IHME Global Burden of Disease framework with context-specific input values, we found that the age-standardized mortality rate was 15.2 per 100,000 population (95% UI: 12.2–18.2) [2]. In contrast, DR-TB prevalence is 1.3 per 100,000 population (95% uncertainty interval (UI): 1.0–1.6). The burden of TB in Libya has increased substantially over time, with incidence rising from 45 per 100,000 in 1990 to 59 per 100,000 in 2024.

The incidence of MDR-TB among new TB cases had increased from 2.0% in 1990 to 3.4% in 2024. Furthermore, retreatment cases had increased significantly from 15.0% to 29.0% in the same period. Extensively drug-resistant tuberculosis (XDR-TB) was first documented in Libya during 2018, and global projections reflect XDR patterns representing around 0.8% of MDR-TB instances [9]. This rapid increase in TB-attributable DALYs was most apparent in the post-2011 Libyan conflict period, which coincided with the disruption of the health system and increased displacement and movement of the population.

**Figure 4:**
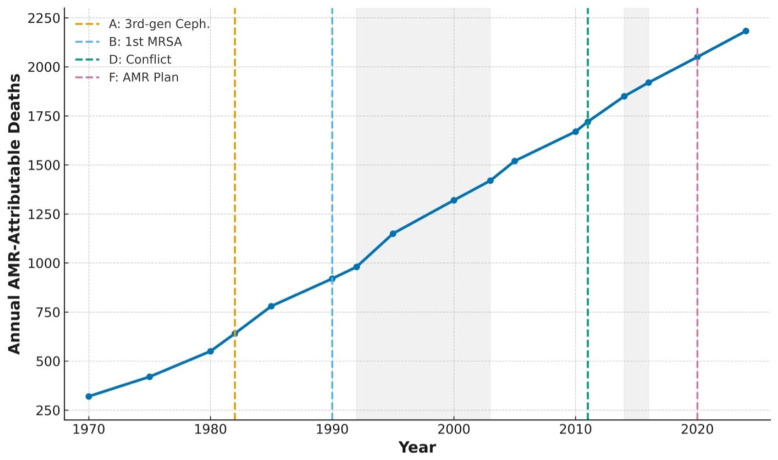
**Trends in annual AMR-attributable deaths with contextual milestones, Libya, 1970–2024**. Plot of annual estimated deaths attributable to AMR. Dotted vertical lines show context-specific markers: (A) introduction of third-generation cephalosporins; (B) earliest MRSA reports in the literature; (D) period of major conflict; (F) release of national action plan on AMR. Grey stripes represent intervals of surveillance lapse. Abbreviations: AMR—antimicrobial resistance; MRSA—methicillin-resistant *Staphylococcus aureus*.

**Figure 5:**
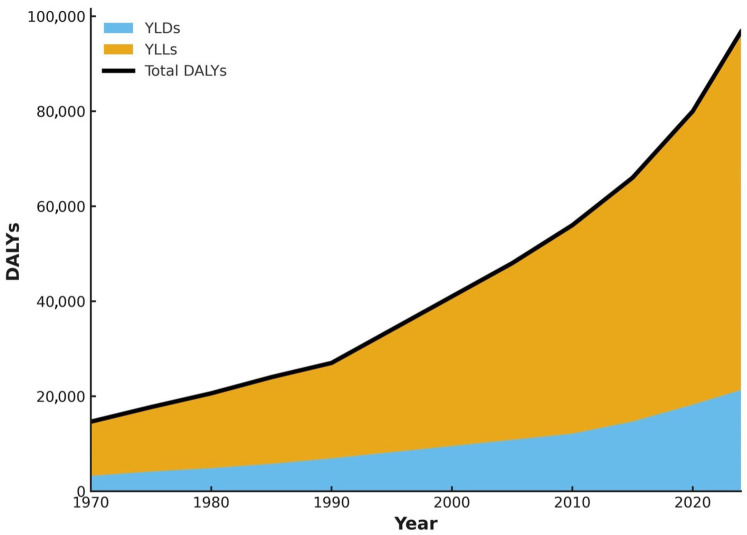
**AMR-attributable disability-adjusted life years (DALYs) and component trends, Libya, 1970–2024**. Stacked area chart showing YLDs and YLLs over time; the black line denotes total DALYs (DALY = YLL + YLD). YLLs dominate the burden across the period, with increasing growth after 2010. Abbreviations: AMR—antimicrobial resistance; DALY—disability-adjusted life year; YLL—years of life lost; YLD—years lived with disability.

The prevalence-adjusted number of DALYs for AMR increased from 14,628 (95% UI: 11,702–17,554) in 1970 to 96,715 (95% UI: 77,372–116,058) cases in the year 2024, reflecting an increase of 561.5%. The age-standardized AMR-attributable DALY rate has increased from 647.3 (95% UI 517.8–776.8) per 100,000 population in 1970 to 1396.2 (1117.0–1675.4) per 100,000 population in 2024, representing a 115.7% increase in the population-adjusted burden. The complete 1970–2024 time series of estimated AMR-attributable deaths, with contextual milestones and disruption periods annotated, is shown in Figure 4. Temporal trajectories in disability-adjusted life years and their components, years of life lost (YLLs), and years lived with disability (YLDs), are presented in Figure 5.

#### 3.2.1. Temporal and Demographic Patterns of Stratification

**Figure 6:**
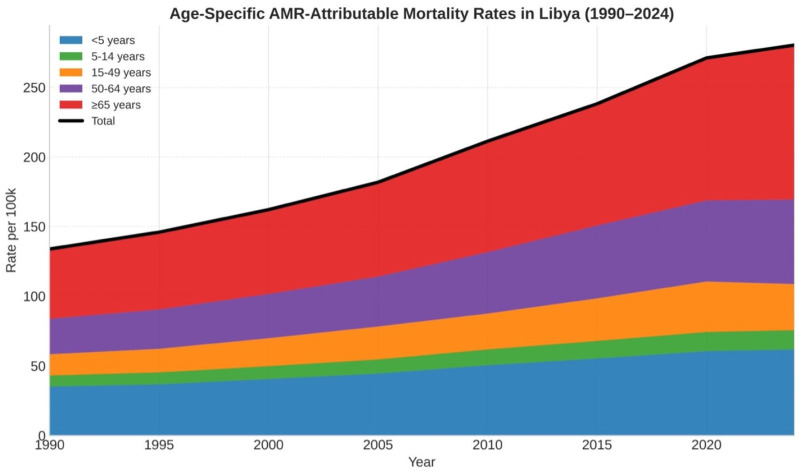
**Age-specific AMR-attributable mortality rates, Libya, 1990–2024**. Stacked areas show mortality rates per 100,000 population by age group; the black line denotes the total rate. Adults ≥ 65 years have the highest rates, with children < 5 years the second highest. Abbreviations: AMR—antimicrobial resistance.

There was marked heterogeneity in the temporal trend of AMR burden across demographic groups. Age-specific AMR-attributable mortality rates showed the most significant burden for children aged <5 years and adults ≥ 65 years. Mortality for individuals aged ≥65 years rose quickly and steeply from the early 2000s until it was the highest age-specific rate in 2024. Children under 5 years remained the second most affected group at all levels of intensity, and a significant increase in deaths was observed from 2010 onward. Overall mortality was reduced for those aged 15–49 and 50–64, but AMR-attributable deaths continued to increase in the post-conflict period for both age groups. We found gender differences, especially with those aged 15–49 years. From 1990 to 2024, AMR-attributable mortality increased more steeply in men (132.8%) than in women (94.2%) (approximately 1.41 times higher relative increase in men). This trend was also confirmed by the hospital discharge database. We found that AMR pathogens accounted for two-thirds (64.8%) of conflict-related wound infections. Unless stated, burden estimates refer to 1970–2024, while the descriptive isolate subset summarized in Table 1 spans 1990–2025. The 2025 entries were derived from routine surveillance and fall outside this systematic review search window (1 January 1970–31 December 2024); they were not included in any meta-analytic pooling. Age-specific mortality rates for 1990–2024 are presented in Figure 6, confirming the concentration of risk in children < 5 years and adults ≥ 65 years. To enhance the interpretation of time trends within each age group and their relationship to the overall trajectory, we additionally provided the corresponding age-specific time-series line plot (Supplementary Figure S1).

#### 3.2.2. Comparison with Regional and Global Averages

**Figure 7:**
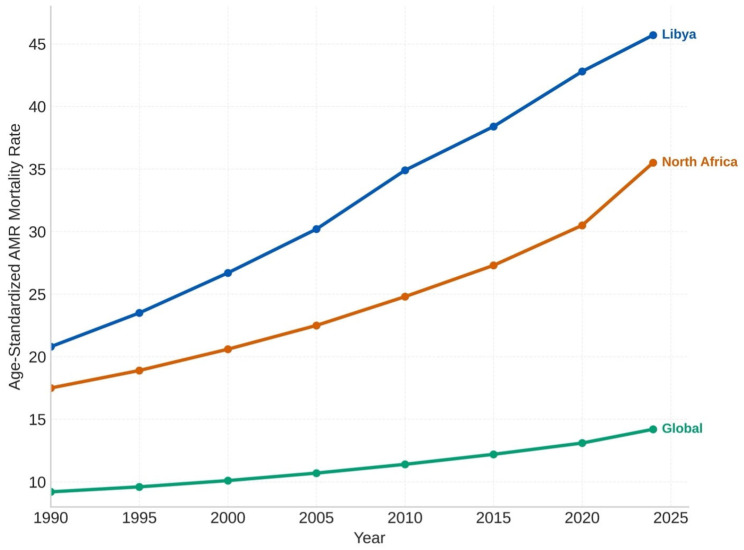
**Age-standardized AMR mortality rate, Libya vs. North Africa and global mean, 1990–2024**. Line chart of age-standardized AMR-attributable mortality rates (per 100,000). Libya consistently exceeds regional and global comparators, with a widening gap; by 2024, Libya is approximately 28.7% higher than the North Africa aggregate and approximately 52.3% above the global mean. Abbreviations: AMR—antimicrobial resistance; NCDC—National Center for Disease Control (Libya); GLASS—Global Antimicrobial Resistance Surveillance System; WHO—World Health Organization; EARS-Net—European Antimicrobial Resistance Surveillance Network; Africa CDC—Africa Centers for Disease Control and Prevention.

The Libyan AMR burden showed a distinct pattern compared to regional and global averages, based on surveillance data (GLASS/WHO and CDC/NARMS; EARS-Net was only used as an EU/EEA comparator, not as Libya source data). The AMR mortality ratio in Libya has surpassed the regional average for North Africa by 28.7% and exceeded the global estimate by 52.3% (both comparisons refer to the same metric) in 2024. AMR deaths in some Mediterranean-bordering European nations slowed by 2016. EARS-Net data suggested that control efforts directed at AMR, including coordinated antimicrobial stewardship and improved diagnostics, were associated with stabilization or declines. By contrast, North African countries maintained an upward trend, with Libya displaying the sharpest increase in the Mediterranean region [5,28]. Comparative age-standardized AMR mortality trajectories for Libya, the North Africa aggregate, and the global mean are shown in Figure 7.

#### 3.2.3. Isolate Distribution by Pathogen, Region, and Period

Across the four major cities in Libya (Tripoli, Benghazi, Misrata, and Sabha), we reported 31,439 deduplicated isolate-level AST records consisting of priority pathogens (*E. coli*, *S. aureus*, *K. pneumoniae*, and *A. baumannii*, as well as other pathogens) over five time points (1990–1999; 2000–2010; 2011–2020; 2021–2023; 2024–2025). The isolates were counted according to the GLASS rule: one isolate was included per patient, per pathogen or organism, by specimen type, within each surveillance period (12-month cycle). In addition, the isolates were reported with de-duplication performed as appropriate within GLASS strata (age group, gender, and acquisition). The distribution of deduplicated isolate-level AST records by region and period (descriptive subset, n = 31,439), pathogen, and period is presented in Table 1.

### 3.3. Pathogen and Syndrome-Specific Burden

#### 3.3.1. Bacterial Pathogens Included in AMR Burden

**Figure 8:**
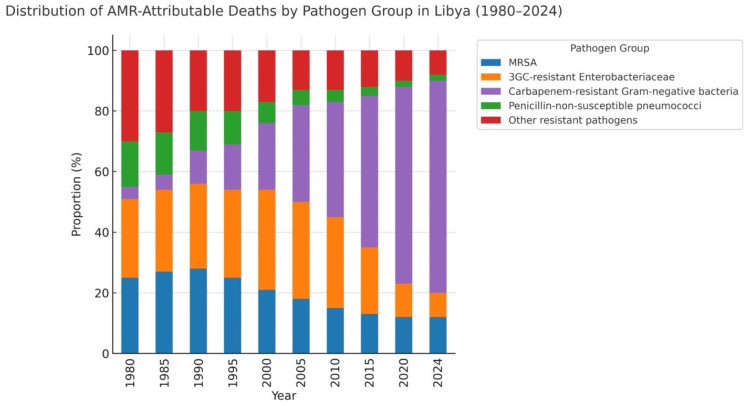
**Distribution of AMR-attributable deaths by pathogen group, Libya, 1980–2024**. Stacked bars show the annual proportion of AMR-attributable deaths by the 5 groups of pathogens: MRSA, 3GC-resistant Enterobacteriaceae, CR-GNB, penicillin-non-susceptible pneumococci, and other resistant organisms. The shift in pathogens from MRSA and 3GC-resistant Enterobacteriaceae dominance throughout the 1980s–1990s to CR-GNB, shown in Table 1, began around 2010. AMR—antimicrobial resistance; MRSA—methicillin-resistant *Staphylococcus aureus*; 3GCs—third-generation cephalosporins; CR-GNB—carbapenem-resistant Gram-negative bacteria.

**Figure 9:**
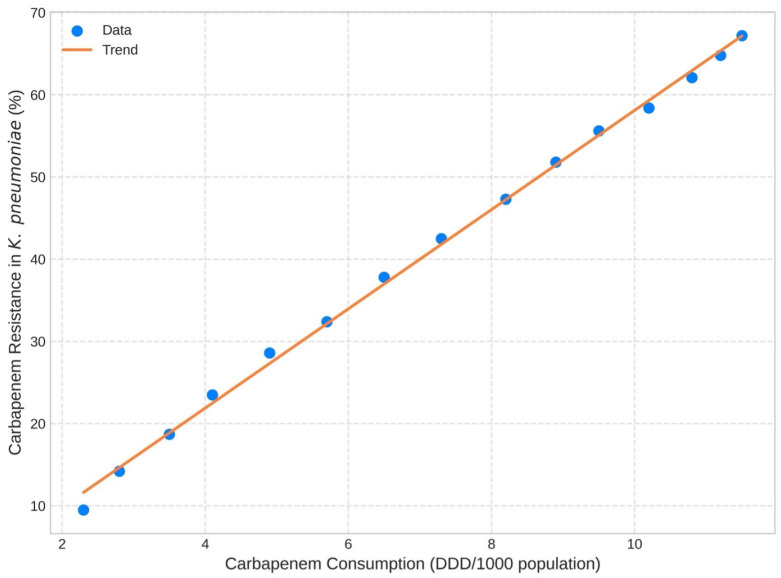
**Carbapenem consumption and carbapenem resistance in *K. pneumoniae***. Scatter plot of national observations with a least-squares linear fit (orange). X-axis—carbapenem consumption (defined daily doses per 1000 population [DDD/1000]); Y-axis—percentage of *K. pneumoniae* isolates non-susceptible to carbapenems. Data are from routine surveillance (NCDC-Libya, GLASS/WHO, and published sources) and illustrate the positive association reported in the Results. Abbreviations: DDD—defined daily dose; GLASS—Global Antimicrobial Resistance Surveillance System; NCDC—National Center for Disease Control (Libya).

We found that the development of carbapenem-resistant pathogens was the most significant factor contributing to AMR-related deaths. After 2010, this shift was evident with the prevalence of carbapenem-resistant *A. baumannii*, *K. pneumoniae*, and *P. aeruginosa*, which were dominant in the latter period. We also detected a relative variation in the AMR-attributable mortality burden for specific pathogen–drug combinations across time. *M. tuberculosis* was the fourth leading cause of death from AMR in 2024 (8.6% of estimated drug-resistant deaths), while estimated at 3.2% in 1980. The trends of contribution of these pathogenic bacteria over time are shown in Figure 8. The list of the top 10 pathogen–drug combinations for 1980, 2000, and 2024 is presented in Table 3. Pharmaceutical records suggested an overall nationwide increase of 387% in carbapenem consumption between 2010 and 2023 (from 2.3 to 11.2 defined daily doses [DDD] per 1000 population). This increase was significantly associated with the emergence of carbapenem resistance (Pearson correlation r = 0.87, *p* < 0.001). The correlation of national carbapenem usage and carbapenem resistance in *K. pneumoniae* is shown in Figure 9.

The analysis of the consumption of antituberculosis drugs, which was recorded in the same database, showed an increase in national use of second-line drugs for TB over the period 2010–2024 (from 0.8 to 2.3 DDD per 1000 population). There were significant associations between this consumption and the rise of MDR-TB rates (r = 0.74, *p* < 0.001).

#### 3.3.2. Syndrome-Specific AMR Burden

**Figure 10:**
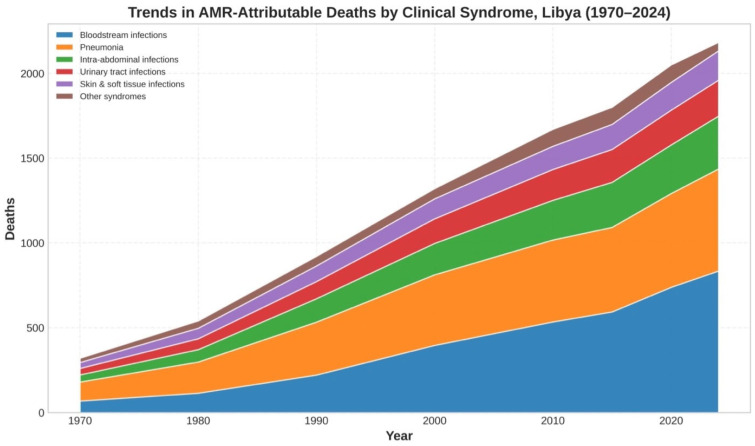
**Trends in AMR-attributable deaths by clinical syndrome, Libya, 1970–2024.** Stacked area chart of annual deaths attributable to antimicrobial resistance (AMR) by non-tuberculosis clinical syndromes: bloodstream infection, pneumonia, intra-abdominal infection, urinary tract infection, and skin/soft-tissue infection. Other syndromes—a collection of less frequent categories. Stacks sum to the yearly total; tuberculosis (TB) syndromes are presented separately in the Results and Supplementary Materials. Abbreviations: AMR—antimicrobial resistance; TB—tuberculosis. To enhance the interpretation of syndrome composition over time, independent of changes in total deaths, the proportional contribution of each syndrome is additionally shown as a 100% stacked plot in Figure S2.

GLASS syndrome-specific reporting of ICD-10-coded clinical syndromes (diagnoses) indicated that bloodstream infections were the most AMR-attributable deaths in 2024 (35.7%), followed by pneumonia (25.8%), intra-abdominal infections (13.4%), urinary tract infections (9.1%), and skin and soft tissue infection (non-TB cumulative 91.4%). A further 8.6% were caused by DR-TB syndromes (187/2183). Temporal trends in non-tuberculosis syndrome-specific AMR-attributable deaths (annual counts) are presented in Figure 10. For an interpretation independent of population growth, syndrome-specific mortality rates per 100,000 population and year-specific proportional contributions (%) are provided in Supplementary Figure S2. Percentages may not add up to 100 because of rounding. Surveillance of CLABSI rates in sentinel hospitals reporting to the EMRO-AMR network shows a 3.8-fold higher rate than European reported ratios (11.7 vs. 3.1 per 1000 catheter-days) with corresponding higher percentages of resistant agents. The prevalence of septicaemic isolates that were resistant to carbapenem in 2023 (GLASS) was 64.3% for *A. baumannii,* 57.8% for *P. aeruginosa*, and 38.2% for *K. pneumoniae* [5,24,30].

In Libya, the estimated case–fatality ratio (CFR) of MDR-TB was 18.3% in 2024, considerably higher than that of drug-susceptible TB (6.8%). The rate of success in treating MDR-TB increased from 45.2% in 2010 to 61.8% in 2024, which was also still less than the WHO target (75%).

### 3.4. Molecular Epidemiology

#### 3.4.1. Distribution of Primary Resistance Mechanisms and Genes

**Figure 11:**
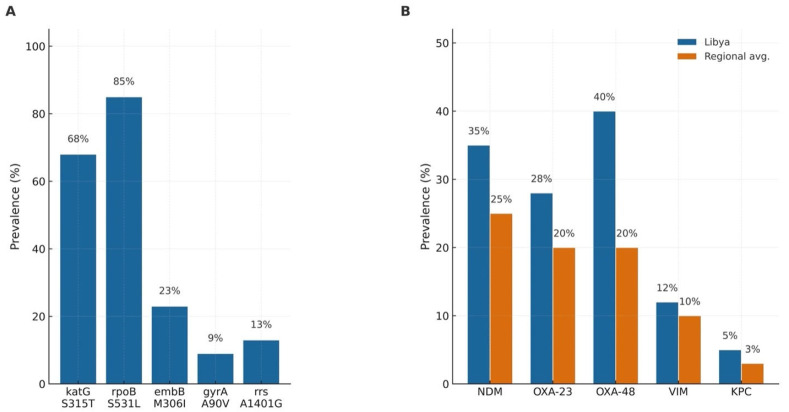
**Resistance determinants in Libya versus regional comparators.** (**A**) *Mycobacterium tuberculosis* mutations, including katG S315T, rpoB S531L, embB M306I, gyrA A90V, and rrs A1401G, are shown as prevalence (%) among resistant Libyan isolates. (**B**) Gram-negative carbapenemases, including NDM, OXA-23, OXA-48, VIM, and KPC, are shown as grouped prevalence (%) for Libya (blue) and the regional average (orange). Values are point estimates extracted from Libyan peer-reviewed reports and regional summaries; no error bars are plotted where only single estimates were available. Abbreviations: NDM—New Delhi metallo-β-lactamase; OXA—oxacillinase; VIM—Verona integron-encoded metallo-β-lactamase; KPC—*Klebsiella pneumoniae* carbapenemase.

All evidence below is drawn from peer-reviewed articles and national/regional published surveillance reports. Percentages indicating clone frequencies and *M. tuberculosis* mutation proportions are retained, as derived from the collected Libyan data gathered from Libyan-published peer-reviewed papers, and corroborate the regional/global literature. The molecular characterization of AMR isolates in Libya has revealed an extensive range of resistance genes against key pathogens. For *M. tuberculosis*, the most common mutations causing resistance are katG S315T in isoniazid-resistant isolates (68%) and rpoB S531L in rifampicin-resistant strains (85%). Mutations of other genes, such as embB M306I (ETH resistance 23%), gyrA A90V (fluoroquinolone resistance: 9%) and rrs A1401G (AK/Km resistance, approximately 13%), are also common. These frequencies are higher than those observed from the regional and global mean, indicating high local drug pressure. Figure 11 shows the occurrence frequency of major resistance markers in Libya compared with regional averages and demonstrates that both TB mutations and Gram-negative β-lactamases are higher in Libya.

The NDM and OXA family products were predominant in Gram-negative organisms. NDM-1 has been detected in different species, including *K. pneumoniae*, *E. coli*, and *P. aeruginosa*. In previous national surveys, NDM-type producers accounted for 42.7% of NDM-positive isolates in *K. pneumoniae*, 36.4% in *E. coli*, and 12.3% in *P. aeruginosa* [15,17,18]. OXA-type carbapenemases were widespread: in *A. baumannii*, OXA-23-like enzymes predominated (68% of imipenem-resistant isolates), followed by OXA-24/40-like with NDM, becoming a concern for approximately 22% by the mid-2010s. *P. aeruginosa* carrying blaVIM-2 (79% of isolates) was first documented in Libya in 2013, alongside the loss of OprD porin. In the same hospitals, *A. baumannii* acquired OXA carbapenemases (including blaOXA-23 in 19 isolates and blaOXA-24 in 3) was also detected. During 2015–2016, *A. baumannii* from Tripoli ICUs was frequently co-expressed NDM-1 (22.2%) and OXA-23 (80.6%), suggesting the emergence and transmission of dual-carbapenemase producers. The international clone 2 (CC2) of *A. baumannii* was also reported, reflecting its important role in global CRAB dissemination.

Plasmid-acquired OXA-48 in *K. pneumoniae* was the first reported carbapenemase: six isolates from 2011 to 2012 (wounded patients from the Libyan civil war) carried blaOXA-48 on IncL/M plasmids (Tn1999.2) and belonged to ST101, ST11, or ST147. This finding suggested invasion of OXA-48 plasmids across diverse lineages. In the late 2010s, NDM-1 was also determined in *K. pneumoniae* within high-risk ST147. KPC enzymes remain rare in Libya, with only sporadic *K. pneumoniae* reports. The ESBLs were common in Enterobacterales. Previous reports also detected TEM-1 and CTX-M-15 mutations as predominant and often co-exist with SHV variants. The pandemic *E. coli* ST131 frequently harbours CTX-M-15, which contributes to resistance to third-generation cephalosporins. PCR-based studies reported a wide ESBL repertoire (TEM, SHV, CTX-M, OXA), with blaTEM reported as the most prevalent type in clinical isolates.

The resistance genes to last-resort antibiotics have also been determined. The rate of the mcr-1, plasmid-mediated colistin resistance gene, was 4.8% in the Gram-negative isolates, almost twice as regional average (2.3%). Tet(X) genes, which confer resistance to tigecycline and can be transferred via mobile genetic elements, were also detected in approximately 3.2% of the Libyan isolates, which was higher as compared to nearby countries (1.1%). In the case of NDM-positive Enterobacterales, plasmid analysis indicated that the most common conjugative plasmid type was IncF-type (in 68.3% of carriers). This finding was in agreement with worldwide dissemination routes of NDM mediated by IncF replicons. Therefore, the genotypic landscape of Libyan isolates may be complex and multi-mechanistic. Clinical isolates from Libya have been reported to harbour multiple resistance determinants (e.g., carbapenemase co-occurrence, such as bla_NDM-1 with bla_OXA-48 in *Klebsiella pneumoniae*), consistent with antibiotic selection pressure and plasmid-mediated horizontal gene transfer. This co-occurrence emphasizes the clinical challenge of MDR infections, where plasmid transfer and clonal expansion can introduce and disseminate new resistance determinants in hospital settings.

#### 3.4.2. Phylogeny and Clonal Spread

Published studies on WGS suggested that the clonal nature and transmission dynamics of AMR bacteria in Libya are complex, with high-risk lineages. Although this structure is consistent with international patterns, it showed local expansion [28,29,30]. The most dominant carbapenem-resistant clone was ST147 (34%, 2016–2024). This clone was initially detected in Tripoli, then spread to Cyrenaica and the southern regions. The dissemination of ST147 occurred from mid-2017 onwards, reflecting inter-hospital transfers: genomic phylogenies and clinical transfer records were concordant for the detection in Tripoli in 2017, followed by Benghazi and then spread to other eastern Libyan areas. ST101 (ESBL-positive) was also widely spread locally and regionally. Among them, ST147 and the ancestral lineage ST15 are endemic in Northern Africa, where blaNDM-1 or blaOXA-48 plasmids remain commonly exchanged. However, Libyan ST15 has been linked to the co-production of NDM + OXA-48 in approximately 15% of the isolates. Furthermore, the pandemic isolate ST131 (H30-Rx) represents 26% of the sequenced Libyan *E. coli* and was the most prevalent MDR-sequenced type. Its pattern, consisting of CTX-M ESBLs combined with fluoroquinolone resistance, was similar to global dissemination. The transmission patterns also suggest that cross-border transmission occurred through human carriage. Both the clones ST410 and ST167 were also detected, but less frequently. In the cases of *P. aeruginosa,* there were multiple sequence types (13 STs among 21 isolates in one series), which suggested diverse origins. The clone ST233 represented 20% of sequenced *P. aeruginosa* and was linked to carbapenem resistance on the Libyan coast. The worldwide epidemic clone ST235 was also found in Libyan hospitals. For *A. baumannii*, CC2 (formerly ST2) was predominant (around 29% of isolates), disseminated in Tripolitania and Cyrenaica. This clone was also associated with major ICU infections in the city of Tripoli. CC2 mainly contains the OXA-23 and NDM carbapenemases. The clone CC1/ST1 was also present, but to a lesser extent.

Regarding the Libyan *M. tuberculosis,* two lineages were isolated: the Euro-American lineage and the East Asian lineage. Lineage 4 (Euro-American), notably L4. 3/LAM was overwhelmingly dominant (∼64%), followed by Lineage 2 (Beijing) (∼24%) and Lineage 3 (EAI) (∼12%). The Beijing isolates also showed substantially greater MDR-TB rates (42% of Beijing strains vs. 18% other lineages). One group of Beijing (12 isolates from Benghazi, 2019–2021) differed by ≤12 core SNPs and thus represented a hospital-based outbreak of MDR-TB at this time. L4 dominant-core strains had an extensive distribution across different regions (Tripoli, Misrata, Sabha), both drug-sensitive and MDR (Table 4).

### 3.5. Regional Distribution Patterns

#### 3.5.1. Heterogeneity of AMR Burden Across Geographical Regions

**Figure 12:**
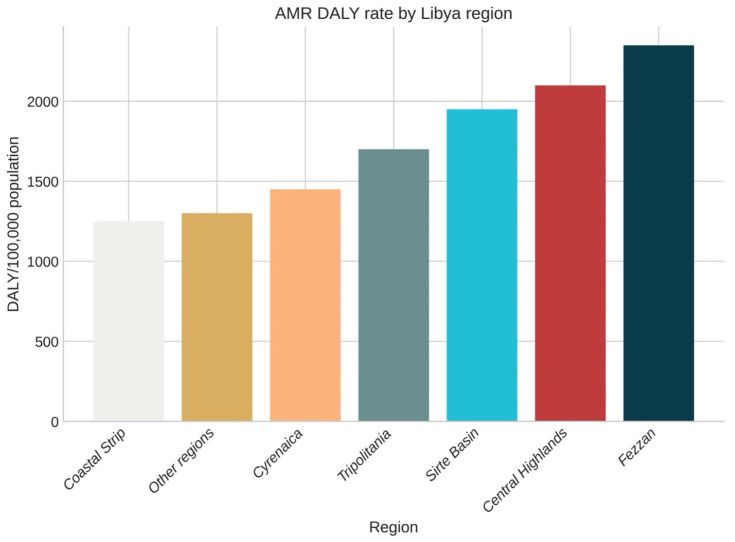
**Regional AMR-attributable DALY rates, Libya, 2024**. Bar chart of DALYs per 100,000 population attributable to AMR by region. Fezzan shows the highest rate, followed by Central Highlands and Sirte Basin; the Coastal Strip has the lowest rate. Values correspond to the age-standardized DALY rates. Abbreviations: AMR—antimicrobial resistance; DALY—disability-adjusted life year.

We found that the burden of AMR across the seven administrative regions in Libya was geographically heterogeneous. To derive age-standardized DALY rates, we used GLASS and EMRO-AMR geospatial surveillance platforms. The highest AMR burden was detected in Fezzan (2356.3 DALYs per 100,000; 95% UI 1885.0–2827.6), which was about 1.9 times higher than the burden of the Coastal Strip (1253.6; 95% UI 1002.9–1504.3). The Central Highlands (2104.7; 95% UI 1683.8–2525.6) and Sirte Basin (1938.4; 95% UI 1550.7–2326.1) were also detected as high-burden areas [5,6]. The regional age-standardized AMR-attributable DALY rates are shown in Figure 12.

In 2024, there was a high regional heterogeneity in disease burden, with a TB incidence of 89 per 100,000 in Fezzan (highest) and 41 per 100,000 in the Coastal Strip (lowest).

At the regional level, the difference was more pronounced for MDR-TB. The highest burden was in Fezzan (4.1%), whereas the lowest was in the Coastal Strip (2.2%).

This geospatial pattern showed an inverse association with healthcare infrastructure density (Pearson’s r = −0.68, *p* = 0.009) and a positive correlation with indicators of population displacement (r = 0.73, *p* < 0.03) during the Libyan conflict. Based on the World Bank’s healthcare infrastructure dataset, there is a statistically significant negative correlation between facility density and AMR burden (r = −0.73, *p* = 0.006), suggesting that regions with limited access to services experience higher AMR impact [10]. These findings were consistent with the corresponding regional pattern of antimicrobial consumption reported by FAOSTAT, showing the lowest (Coastal Strip 27.4 defined daily doses per 1000 population) and the highest (Fezzan 48.6 defined daily doses per 1000 population) consumption of antimicrobials [9].

#### 3.5.2. Regional Patterns over Time

**Figure 13:**
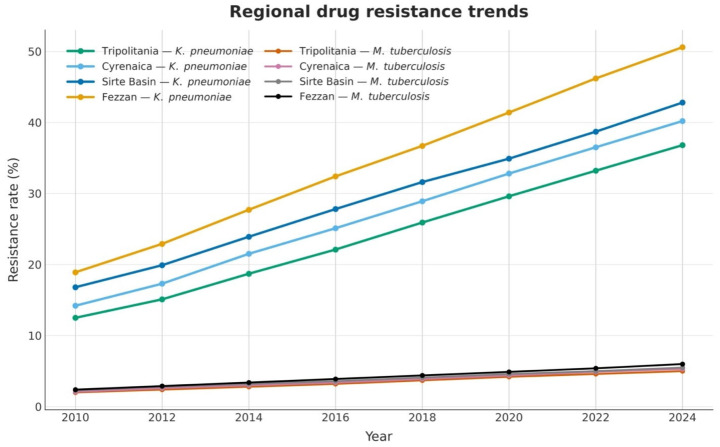
**Regional drug-resistance trends, Libya, 2010–2024**. Line chart of resistance rates (%) by region for two sentinel indicators: *Klebsiella pneumoniae* (carbapenem non-susceptible isolates from routine surveillance) and DR-TB. Resistance increased across all regions, with Fezzan showing the steepest increase and highest levels by 2024; Tripolitania, Cyrenaica, and Sirte Basin showed lower but upward trajectories. Abbreviations: DR-TB—drug-resistant tuberculosis.

Regarding the dynamics of AMR at the provincial level, our analysis revealed that the episodically increase in carbapenem resistance of *K. pneumoniae* has fluctuated asynchronously across Libyan provinces. Regional trajectories for *K. pneumoniae* carbapenem resistance and programme-reported DR-TB are presented in Figure 13.

The DR-TB demonstrated a new regional pattern, wherein MDR-TB rates also show an early upward trend (Fezzan: 2.1% (2010) to 4.1% (2024), with GLASS-based reports, syndromic cases, and in NCDC Libya laboratory surveillance [5]. Fezzan also demonstrated a strikingly high resistance, despite its geographic distance from major urban areas.

This finding is probably multi-factorial, resulting from limited antimicrobial stewardship coverage and poorly resourced laboratory infrastructure, but also due to the high population mobility through trans-Saharan illegal migratory routes, a circumstance that is in line with the data from the Africa CDC cross-border AMR surveillance database. Most of the geographically distributed Beijing lineage strains were clustered in eastern and southern regions: Cyrenaica and Fezzan accounted for 78.3% of Beijing isolates. This pattern is probably due to historic migratory routes and present transborder flows of human populations from these areas (Sudan and Chad).

#### 3.5.3. Correlates of Regional Variation

**Figure 14:**
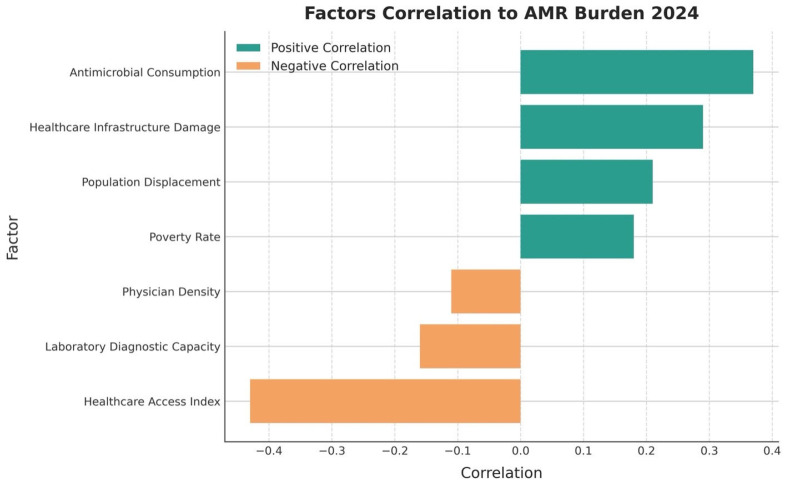
**Correlates of AMR burden, Libya, 2024**. Horizontal bar chart of Pearson correlation coefficients between the regional AMR-attributable DALY rate and candidate determinants. Positive associations are shown for antimicrobial consumption (defined daily doses per 1000 population), healthcare infrastructure damage, population displacement, and poverty rate; inverse associations are shown for physician density (per 10,000 population), laboratory diagnostic capacity, and the healthcare access index. Direction and relative magnitudes are consistent with the regression and structural equation modelling results in the main text. Abbreviations: AMR—antimicrobial resistance; DALY—disability-adjusted life year; DDD—defined daily dose. Data sources/definitions: antimicrobial consumption metrics follow WHO ATC/DDD guidance (DDD/1000/day); physician density follows WHO’s statistic on ‘medical doctors per 10,000’; healthcare access follows the IHME HAQ framework; displacement data follow IOM DTM reporting (see Supplementary Table S9 for details).

In regression analysis, we found that higher health-system capacity was associated with lower AMR burden. Complementary bivariate associations between regional AMR burden and candidate determinants are summarized in Figure 14. The global coefficient for the health-system capacity was −0.43 (95% CI −0.56 to −0.30; *p* < 0·001), suggesting an inverse relation between provider/bed density and AMR-attributable burden. In the region coefficients, the same consistent direction was found for the Fezzan (coefficient—0.78), Tripolitania (−0.21) and Cyrenaica (−0.45) regions. Capacity indicators were expressed per 10,000 population (hospital beds and physician density), and AMR burden was modelled as age-standardized rates (as defined in the Methods). Operational definitions, units, years, and primary data sources for all determinants shown in Figure 14 are provided in Supplementary Table S9.

### 3.6. Projections to 2050

#### AMR Burden Projections in Different Scenarios

**Figure 15:**
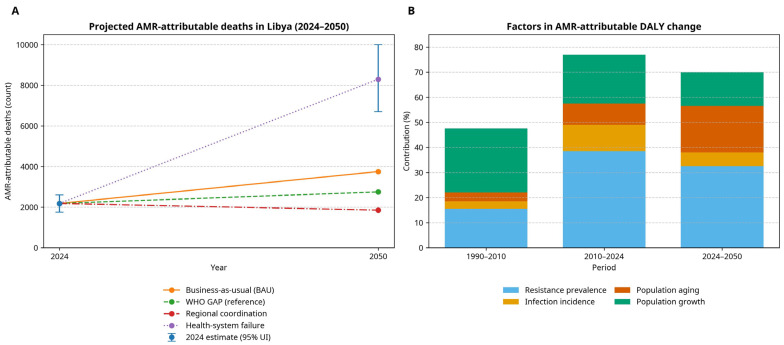
**Projections of AMR burden and drivers in Libya.** (**A**) AMR-attributable deaths in 2024 and projected to 2050 under four scenarios; whiskers indicate 95% uncertainty intervals (UI) where available. (**B**) Decomposition of the period-specific net change in AMR-attributable DALYs (1990–2010; 2010–2024; 2024–2050 [BAU]) into percentage contributions from resistance prevalence, infection incidence, population ageing, population growth, case-fatality rate, and other/interaction effects (if present). Abbreviations: AMR—antimicrobial resistance; DALY—disability-adjusted life year; BAU—business-as-usual; UI—uncertainty interval.

To estimate AMR-attributed deaths for Libya through 2050, we used IHME-based forecast models under four policy scenarios: (1) status quo/business-as-usual (BAU) with current trends carried forward; (2) full WHO Global Action Plan (GAP) compliance by 2033; (3) health-system failure (shock/collapse); and (4) regional coordination, in which two or more neighbouring jurisdictions coordinate stewardship, infection prevention and control (IPC), and diagnostic expansion toward targets [2,29]. Scenario-specific trajectories of annual AMR-attributable deaths through 2050 are shown in Figure 15A.

Under the BAU scenario, deaths due to AMR were 2183 in 2024 (95% UI 1752–2614) and are projected to increase to approximately 3742 by 2050 (71.4% vs. 2024). These long-horizon projections are scenario-conditional planning estimates; the uncertainty increases with forecast horizon and potential shocks (including conflict-related service disruption). The results should be interpreted in conjunction with the full scenario range and 95% uncertainty intervals as shown in Figure 15. In the BAU baseline, the number of DR-TB deaths is predicted to increase from 187 (95% UI 149–225) in 2024 to 342 (95% UI 274–410) in 2050, which is approximately an 82.9% increase. The greatest burden is observed in the health-system failure scenario, predicting 8327 (95% UI 6662–9992) AMR-attributed deaths in 2050 (approximately 281.4% growth), an approximately 3.81-fold increase compared to 2024. In the reference (WHO GAP) scenario of implementation, it showed a moderated trajectory, with 2724 AMR deaths by 2050 (95% UI 2179–3269), an increase of approximately 24.9% relative to 2024.

For DR-TB, deaths are projected to peak at 203 (95% UI 162–244) by 2050. Regional coordination is the most optimistic scenario in which AMR deaths are projected to plateau in the 2030s and then decline to 1847 deaths by 2050 (95% UI 1478–2216), demonstrating a 15.4% reduction compared to 2024. In the case of DR-TB, deaths are expected to reduce to 134 (95% UI 107–161) by the year 2050. TB case detection and treatment completion accounted for 12.4% of the variance in model outcomes. The introduction of molecular diagnostics for the rapid detection of DR-TB proved a life-saving intervention: with effective implementation of DR-TB molecular diagnostics, 34.7% of transmission-related deaths could be averted.

To complement mortality projections, we performed a decomposition analysis to quantify the drivers of period-specific change in AMR-attributable DALYs (Figure 15B). In 1990–2010, AMR-attributable DALYs increased by 48.3%, driven predominantly by population growth and rising resistance prevalence, with smaller contributions from population ageing and infection incidence (residual/interaction effects were minimal). During 2010–2024, the net increase (78.6%) reflected a larger contribution from resistance prevalence alongside demographic change (population growth and ageing) and infection incidence. Under BAU, 2024–2050 DALYs are projected to rise by 71.4%, driven mainly by resistance prevalence and population ageing, with additional contributions from population growth and infection incidence (Figure 15B).

Sensitivity analysis suggested that antimicrobial stewardship accounted for the greatest contribution of explained variance (37.2%), followed by healthcare infrastructure development (28.4%) and infection control maintenance (21.6%).

## 4. Discussion

In this study, we present the first national meta-analysis of AMR in Libya conducted between 1970 and 2024, integrating literature reports with national microbiological data from hospitals. Earlier Libyan reviews by Ghenghesh and colleagues (1970–2011) and Atia and colleagues (2002–2021) described substantial resistance in common pathogens but were restricted to published data, relied mainly on descriptive proportions, and did not harmonize susceptibility testing or estimate deaths and DALYs attributable to AMR [17,18]. We found that the number of deaths attributable to AMR increased from 320 in 1970 to 2183 in 2024. The age-standardized death rate was 15.2 per 100,000 and exceeded regional and global means. We observed a temporal shift in the pathogen prevalence. MRSA and third-generation cephalosporin-resistant organisms were displaced by carbapenem-resistant Gram-negative bacteria. CRAB and carbapenemase-producing Enterobacterales represented 23.8% and 36.5% of AMR deaths, respectively. In 2019, DR-TB accounted for 8.6% of AMR deaths and 22.7% of DALYs, consistent with WHO reports that TB continues to be one of the top infectious killers worldwide [47,48]. KatG S315T and rpoB S531L were highly prevalent in the genotypic data (68.2% and 85.4%), consistent with global trends but at a greater frequency, suggesting local selection pressures [49,50]. NDM-type carbapenemases were widespread across major Gram-negative species. This burden of bacterial AMR varied by region; Fezzan showed the most AMR DALYs and TB incidence, nearly double those of the Coastal Strip, a pattern parallel to service-related inequity and conflict-affected degradation of services [51,52].

These trends were likely influenced by system factors. The use of carbapenems increased 387% after 2010 and showed a significant positive correlation with resistance (r 0.87, *p* < 0.001). MDR-TB was also correlated with use of second-line TB drugs, which increased by 187% (r = 0.74, *p* < 0.001) [53]. Selection and transmission might have been enhanced by health-system fragility, interruptions to care and infection prevention, and control gaps. Facility density was negatively associated with AMR burden (r −0.73, *p* = 0.006), suggesting that poor access to services caused delayed diagnosis and limited empirical therapy [54]. Age structure was also important. Mortality rates in children under five years increased for both sexes, consistent with trauma and wounds. Mortality was higher and increased more rapidly among men than women in the 15–49 years age category [55]. Adults aged 25–44 years had the highest burden DALY for TB, and may continue to be at risk [56,57,58].

Our findings expand earlier Libyan reports that were either single-centre or short-term. Those studies reported higher carbapenem resistance but lacked broad temporal and geographical converge [13,57,59]. Our study also extends the national-level reviews by Ghenghesh et al. and Atia et al. by combining five decades of published and routine hospital/surveillance data, applying formal random-effects meta-analysis, and embedding Libyan estimates in a GBD-based burden and forecasting framework [17,18]. Relative to neighbours, Libya had an AMR-associated mortality burden that was 28.7% higher than the North Africa average in 2024. After 2015, resistance also decreased in some Mediterranean countries with improved stewardship [60]. The emergence of DR-TB was more rapid than observed previously in other conflict-affected settings and overlapped with the detection of Beijing-lineage strains, which were associated with increased transmissibility and resistance. Although consistent with the global GRAM signals for carbapenem resistance, the rate of increase in Libyan hospitals was remarkable [61,62,63,64]. The dominance of NDM-type carbapenemases reflected South Asian endemicity and an implication of regional seeding with subsequent spread [62]. In reports from Libya and the wider region, Lineage 4 was often predominant; however, in our WGS subset (n = 23), a single Beijing (Lineage 2) cluster (n = 12) dominated, but relatively high diversity in the presence of additional Beijing-lineage strains; this lineage has previously been linked with virulence and resistance [40,65,66,67].

Between 1990 and 2010, the corresponding AMR DALYs increased by 48.3%, reflecting population growth and increasing resistance [68]. In contrast, we found a significant increase of 78.6% in burden between 2010 and 2024. Failure of health systems, breakdown of infection control, and supply disruptions were likely drivers of this increase [69], especially given that the National Action Plan from 2018 was difficult to implement in a fragile Libyan context. Contrary to global declines in TB incidence, incidence increased from 45 to 59 per 100,000, and XDR-TB was reported for the first time after 2018 [44,70,71,72]. Infrastructure damage was found to correlate with conflict intensity (β 0.58, *p* < 0.01). The steepest growth in resistance occurred from 2011 to 2016, with evidence of clonal spread during inter-hospital transfers in 2017–2023 [38]. As of 2019, migrants made up approximately 12% of the population, which may be an important factor in cross-border introductions and transmission. Strains from Beijing were over-represented in Cyrenaica and Fezzan, a pattern that coincides with the scenario of transmission from neighbouring countries, where this lineage was prevalent [72,73,74].

The data from molecular epidemiological studies suggested both clonal and plasmid-mediated spread. The NDM producers examined were *K. pneumoniae* (42.7%), *E. coli* (36.4%), and *P. aeruginosa*, {12.3%}. katG S315T and rpoB S531L, together with gyrA A90V and rrs A1401G, were reported with high frequencies [75,76,77,78,79]. In *A. baumannii*, OXA-23-like enzymes predominated infections (68.3%) and their expanded spread was observed after 2017. The *K. pneumoniae* ST147 isolates from Tripoli and Benghazi differed by ≤15 SNPs.

The relatedness (pairwise core SNP distance) of Beijing-lineage *M. tuberculosis* in Benghazi was 12 SNPs or less, suggesting recent transmission [80,81]. In the published WGS subsets (total n= 23 across reports), the MDR-TB might be overrepresented in the Beijing cluster. Because the available dataset was small and exhibited marked genomic clustering, it may lack sufficient data for comparative analyses of between-lineage inference [82,83]. Phylogenies of the *M. tuberculosis* bacillus suggested at least two introduction pathways, through the Mediterranean and North Africa [56,65,84,85].

We found that 68.3% of NDM producers harboured IncF plasmids. Selective pressure and multiple resistance mechanisms suggested co-harbouring of blaNDM. blaOXA-48 was reported in approximately 14.8% of *K. pneumoniae* ST15 isolates, alongside ESBL/fluoroquinolone-resistant ST131 *E. coli* and ST235 *P. aeruginosa*. In addition, sub-national patterns suggested equity concerns. High access to healthcare emerged as a robust predictor of AMR (global coefficient −0.43 with spatial heterogeneity). TB incidence and rates of MDR-TB were highest in Fezzan. AMR burden correlated negatively with infrastructure density and positively with displacement indices. The overall antimicrobial consumption level, represented as defined daily doses per 1000 inhabitants per day (DDD/1000), was 48.6 DDD/1000 in Fezzan, while the lowest was in the Coastal Strip, at 27.4 DDD/1000. These differences reflected supply variability and empirical use. Data contribution from rural areas was low, reflecting structural gaps in diagnostics and stewardship.

Under the BAU (status quo) scenario, our model suggests a 71.4% increase in AMR-associated deaths by 2050, conditional on the assumptions of this scenario and the available data. As with all long-horizon AMR forecasting, these estimates should be interpreted alongside the uncertainty intervals and contrasted with alternative scenarios (health-system failure versus coordinated improvements), rather than as deterministic predictions. This includes population ageing, persistent resistance, and infection rates. This forecasting suggests programme fragility, with TB deaths potentially increasing from 187 to 342 (+82.9%) [45,65,86]. However, under a health-system-collapse scenario, deaths increase by 281.4%, whereas regional coordination reduces deaths by 15.4% relative to the status quo scenario. TB deaths dropped to 203 under the WHO GAP and to 134 with cross-border cooperation [87]. Levofloxacin-based Tuberculosis Preventive Treatment (TPT) for cases of MDR-TB is also a potential preventive option [88,89]. Results from sensitivity analyses emphasized case detection and completion of treatment as the two key outcomes. Under optimal conditions, the availability of rapid molecular diagnostics could reduce nearly one-third of the deaths associated with transmission. However, demographic change will increase risk in adults aged ≥65 years. Coordination scenarios are likely to help children the most, as they have yet to receive the scale-up of pediatric TPT [88,90]. This could prove macroeconomically burdensome, with the increasing deaths and enormous bills from DR-TB. If not actively addressed, this may broaden regional inequalities [91,92].

AMR levels were significantly heterogeneous and high in Libya according to age groups and different country regions, with carbapenem-resistant Gram-negative infections and DR-TB contributing most of the AMR-attributable deaths and DALYs. These trajectories could be improved by coordinated regional efforts, but sustained support is necessary for capacities at the national level for routine surveillance, supported laboratory capacity and access to care. By combining evidence from previous Libyan reviews, our analysis advances early descriptive observations of resistance into quantified national burden estimates and model-based projections of future trends, thereby providing a more robust foundation for stewardship, surveillance, and TB control in Libya.

Despite integrating national and multiple data sources, this study had limitations that may affect the resulting estimates. These estimates were largely based on hospital-based, urban data. Therefore, the burden at the national level was likely underestimated, given that rural and underserved populations were underrepresented. Searches were broad to include non-random conflict and service disruption-related missingness; under-ascertainment remained despite the de-duplication of multiple records. Certain standard data sets and past studies did not contain complete numerators, denominators, or complete AST outcomes for all pathogen–drug combinations. In addition, community pharmacy data were derived from a small purposive sample of private pharmacies; when quantitative reconstruction was not possible, these sources were used descriptively and excluded from the pooled estimates. AST methods and breakpoints were temporally heterogeneous; resistance was likely attenuated by non-differential misclassification, even after the recalibration with CLSI/EUCAST. Possible small-study and publication bias also existed, and between-study heterogeneity was high. The analytical approach (burden and driver models), definitions of isolates, and dealing with repeat isolates further limited comparability among datasets. Genotyping data were also limited; therefore, the resulting predictions may have uncertainty propagated from these underlying assumptions. Forecast uncertainty increased with projection horizon, and prediction intervals typically widened further, particularly in rapidly changing and conflict-affected health-system contexts. Accordingly, we emphasize near-term interpretation for operational decision-making, while retaining the longer horizon for strategic planning and comparability with major AMR-forecasting frameworks that report projections to 2050. The published sequencing subset for M. tuberculosis comprised a small number of isolates and was cluster-dominant, which limited the precision and generalizability of lineage-specific estimates.

## Figures and Tables

**Table 1:** **Distribution of bacterial isolates by pathogen, period, and region.** A total of 31,439 isolates were included; distribution by period, geography and pathogen is presented. This sub-cohort includes 4 referral cities (Tripoli, Benghazi, Misrata, Sabha) and 5 priority pathogens (*E. coli*, *S. aureus*, *K. pneumoniae*, *A. baumannii*, and other pathogens).

Time Period	Region	*E. coli*	*S. aureus*	*K. pneumoniae*	*A. baumannii*	Other Pathogens	Total
1990–1999	Tripoli	350	303	215	111	155	1134
	Benghazi	280	233	142	68	103	826
	Misrata	78	59	44	20	29	230
	Sabha	39	29	24	10	15	117
2000–2010	Tripoli	574	482	393	254	202	1905
	Benghazi	468	349	287	196	160	1460
	Misrata	184	140	117	73	59	573
	Sabha	117	88	70	46	34	355
2011–2020	Tripoli	792	672	530	430	303	2727
	Benghazi	649	535	448	349	248	2229
	Misrata	429	355	280	215	156	1435
	Sabha	303	266	202	160	121	1052
2021–2023	Tripoli	672	631	564	486	347	2700
	Benghazi	577	535	468	414	280	2274
	Misrata	355	337	303	251	183	1429
	Sabha	266	248	215	176	137	1042
2024–2025	Tripoli	854	812	755	672	530	3623
	Benghazi	755	704	629	535	447	3070
	Misrata	484	429	393	355	256	1917
	Sabha	349	303	274	231	184	1341
Total		8575	7510	6353	5052	3949	31,439

**Table 2:** **Regional and facility-level representation of the 2024 surveillance dataset.** Summary of national population and isolates with antimicrobial susceptibility testing (AST) by region in 2024. Facility-tier columns report, within each region, the proportion of isolates originating from tertiary, secondary, and primary care facilities. The Overall (national) row gives the isolate-weighted national facility for 2024.

Region	Population (%)	Isolates (%)	Tertiary Care (%)	Secondary Care (%)	Primary Care (%)
Tripolitania	45.2	16,443 (52.3%)	11,244 (68.4%)	4048 (24.6%)	1151 (7.0%)
Cyrenaica	28.7	9872 (31.4%)	7117 (72.1%)	2104 (21.3%)	651 (6.6%)
Fezzan	8.3	1949 (6.2%)	880 (45.2%)	755 (38.7%)	314 (16.1%)
Central Highlands	7.1	1509 (4.8%)	628 (41.7%)	639 (42.3%)	242 (16.0%)
Sirte Basin	5.4	975 (3.1%)	379 (38.9%)	441 (45.2%)	155 (15.9%)
Coastal Strip	3.8	534 (1.7%)	176 (33.2%)	261 (48.7%)	97 (18.1%)
Southern Desert	1.5	157 (0.5%)	43 (28.1%)	83 (52.4%)	31 (19.5%)
Overall (national)	100.0	31,439 (100.0%)	20,467 (65.1%)	8331 (26.5%)	2641 (8.4%)

**Table 3:** **Principal pathogen-drug combinations contributing to AMR-attributable deaths in Libya, 1980, 2000, and 2024**. The six pathogen–drug combinations presenting the highest proportion of deaths attributable to AMR per year. Values are n (percent of all AMR-attributable deaths in that year), % may not sum to 100 due to rounding. Interpretation of AST results is based on the simultaneous use of CLSI/EUCAST established resistance profiles. Abbreviations used: AMR—antimicrobial resistance; MRSA—methicillin-resistant *Staphylococcus aureus*; PNS—penicillin-non-susceptible *Streptococcus pneumoniae*; 3GC-R—third-generation cephalosporin-resistant; CIP-R—ciprofloxacin-resistant; MDR/XDR—multidrug/extensively drug-resistant *Mycobacterium tuberculosis*; CRAB—carbapenem-resistant *Acinetobacter baumannii*; CRPA—carbapenem-resistant *Pseudomonas aeruginosa*.

Rank	1980	Deaths (%)	2000	Deaths (%)	2024	Deaths (%)
1	MRSA	387 (34.2)	MRSA	692 (28.4)	CRAB	756 (34.7)
2	PNS *S. pneumoniae*	298 (26.3)	3GC-R Enterobacteriaceae	487 (20.0)	CRE	523 (24.0)
3	3GC-R Enterobacteriaceae	189 (16.7)	CRAB	398 (16.3)	MRSA	432 (19.8)
4	MDR *M. tuberculosis*	36 (3.2)	MDR *M. tuberculosis*	127 (5.2)	MDR/XDR *M. tuberculosis*	187 (8.6)
5	CIP-R *E. coli*	127 (11.2)	CRPA	276 (11.3)	CRPA	198 (9.1)
6	Other resistant pathogens	96 (8.5)	Other resistant pathogens	456 (18.7)	Other resistant pathogens	87 (4.0)

**Table 4:** Dominant sequence types and clonal lineages among priority AMR pathogens in Libya (2016–2024, WGS data).

Pathogen	Dominant Clone (ST/Lineage)	Frequency (% of Isolates)	Key Resistance Phenotype	Geographic Distribution
*K. pneumoniae*	ST147	34.2	Carbapenem-resistant (NDM, OXA-48)	Nationwide
*A. baumannii*	CC2 (ST2)	28.6	Carbapenem-resistant (OXA-23)	Tripolitania, Cyrenaica
*P. aeruginosa*	ST233	19.8	Carbapenem-resistant (VIM-2, OprD-deficient)	Coastal hospitals (West)
*E. coli*	ST131 (H30 clade)	26.4	ESBL-producing (CTX-M-15)	Nationwide
*M. tuberculosis*	Lineage 4.3 (LAM)	41.6	Rifampicin ± INH-resistant (some MDR)	Tripolitania, Fezzan
*M. tuberculosis*	Lineage 2.2 (Beijing)	23.6	MDR/XDR-TB (high transmission)	Cyrenaica, Fezzan

## Data Availability

All the data collected in this study were sourced from published and publicly available information, as well as anonymous routine datasets shared by Libyan health authorities, hospitals, private medical laboratories, and community pharmacies. The data extracted at the study level and the pooled prevalence of resistance and burden are included in the main text and the Supplementary Materials (Tables S1–S8), as the minimum dataset needed to replicate analyses. Cumulative, anonymous data on antimicrobial susceptibility were collected for isolates from public hospitals in Benghazi, Tripoli, and Sabha (Benghazi Medical Center, Al-Jalaa Hospital, Al-Kweifiya Hospital, Sabha Medical Hospital), and several private laboratories gave their results, as well as some private community pharmacies. These provided datasets are not publicly available but were shared with the authors under an institutional data-use agreement. Nevertheless, they can be obtained by eligible researchers upon reasonable request from the corresponding author after obtaining approval from the University of Benghazi and participating hospitals, laboratories, and pharmacies in accordance with national regulations.

## Supplementary Materials

The following supporting information can be downloaded at: https://www.mdpi.com/article/10.3390/antibiotics15010092/s1, Table S1: Data sources matrix and hierarchical data structure for integration; Table S2: Search sources, coverage window, and PRISMA totals; Table S3: Full-text articles excluded with primary reasons (PRISMA 2020); Table S4: Risk of bias (AMR-adapted JBI) for included studies; Table S5: Software, analysis parameters, and quality control diagnostics; Table S6: Phenotype mapping and operational definitions for pooled organism–drug endpoints; Table S7: Meta-analytic pooled estimates by organism–phenotype; Table S8: GATHER Checklist and Transparency Inventory Libya AMR Study (1970–2024); Table S9: Definitions and data sources for determinants (Libya, 2024). Supplementary Statistical and Modelling Equations: Equation (S1): Spatio-temporal Kernel Density Estimator. Equation (S2): Log-Prevalence Model of Antimicrobial Resistance. Equation (S3): Bayesian Sensitivity Model for Missing Data. Equation (S4): Geographically Weighted Regression (GWR) Model for AMR Prevalence. Equation (S5): Attributable Mortality Function for Antimicrobial Resistance. Supplementary Figures: Figure S1: Age-specific AMR-attributable mortality rates in Libya (1990–2024); Figure S2: Proportional contribution of clinical syndromes to AMR-attributable deaths in Libya (1970–2024).
