# Supplementary material for: Burden of Bacterial Antimicrobial Resistance in Libya, 1970–2024: A Systematic Meta-Analysis with Projections to 2050
Source: Antibiotics (Basel). 2026 Jan 16;15(1):92. doi: 10.3390/antibiotics15010092 (PMC12837905; doi:10.3390/antibiotics15010092)
Supplement: Supplementary file 1 [file antibiotics-15-00092-s001.zip › PRISMA 2020 checklist_V9.pdf]

PRISMA

PRISMA 2020 Checklist

Section and Topic	Item #	Checklist item	Location where item is reported
**TITLE**			
Title	1	Identify the report as a systematic review.	Title page -manuscript title clearly identifies this work as a systematic review and meta-analysis.
**ABSTRACT**			
Abstract	2	See the PRISMA 2020 for Abstracts checklist.	Abstract -single-paragraph summary describing background, objectives, methods (PRISMA-2020 systematic review and meta-analysis), key results, and conclusions.
**INTRODUCTION**			
Rationale	3	Describe the rationale for the review in the context of existing knowledge.	Introduction -first three paragraphs outlining the global and Libyan context for AMR and gaps in existing evidence.
Objectives	4	Provide an explicit statement of the objective(s) or question(s) the review addresses.	Introduction- stating the objectives and questions; complemented by Methods, Section 1 'Study Design'.
**METHODS**			
Eligibility criteria	5	Specify the inclusion and exclusion criteria for the review and how studies were grouped for the syntheses.	Methods -Section 3 'Systematic Review and Meta-analysis' (paragraphs describing inclusion and exclusion criteria for studies, populations, specimens, and AST methods).
Information sources	6	Specify all databases, registers, websites, organisations, reference lists and other sources searched or consulted to identify studies. Specify the date when each source was last searched or consulted.	Methods -Sections 2 'Data Sources and Collection' and 3 'Systematic Review and Meta-analysis'; Supplementary

PRISMA 2020 Checklist

Section and Topic	Item #	Checklist item	Location where item is reported
			Information -Table S1 (description of databases, surveillance systems, laboratories, and registries used as information sources).
Search strategy	7	Present the full search strategies for all databases, registers and websites, including any filters and limits used.	Methods -Section 3 'Systematic Review and Meta-analysis' (search strategy description, databases, date ranges and keywords); Supplementary Information -Table S2 (full Boolean search strings and limits for each database).
Selection process	8	Specify the methods used to decide whether a study met the inclusion criteria of the review, including how many reviewers screened each record and each report retrieved, whether they worked independently, and if applicable, details of automation tools used in the process.	Methods -Section 3 'Systematic Review and Meta-analysis' (title/abstract and full-text screening by two independent reviewers, de-duplication rules); Results -Section 1 'Study selection and characteristics of included studies'; Figure 1 (PRISMA 2020 study selection flow diagram).
Data collection process	9	Specify the methods used to collect data from reports, including how many reviewers collected data from each report, whether they worked independently, any processes for obtaining or confirming data from study investigators, and if applicable, details of automation tools used in the process.	Methods -Sections 2 'Data Sources and Collection' and 3 'Systematic Review and Meta-analyses, and 4.1 'Data Integration and Management' (standardised data abstraction forms, ETL pipeline, extraction of numerators/denominators and covariates).

Section and Topic	Item #	Checklist item	Location where item is reported
Data items	10a	List and define all outcomes for which data were sought. Specify whether all results that were compatible with each outcome domain in each study were sought (e.g. for all measures, time points, analyses), and if not, the methods used to decide which results to collect.	Methods – Section 3 “Systematic Review and Meta-analysis” (primary outcome: prevalence of non-susceptible/resistant isolates for each pathogen–drug combination; definitions of AMR metrics); Sections 6–7 “Burden Estimation” and “Forecasting and Scenario-Based Modelling” (outcomes: AMR-attributable deaths, DALYs, YLL, YLD, mortality rates, and projections to 2050).
	10b	List and define all other variables for which data were sought (e.g. participant and intervention characteristics, funding sources). Describe any assumptions made about any missing or unclear information.	Methods -Sections 2 ‘Data Sources and Collection’, 4.1 ‘Data Integration and Management’, 5.1 ‘Geographic Stratification’, 9 ‘Integration of Laboratory and Surveillance Data’, and 10 ‘Microbiological and Molecular Framework’; Supplementary Tables S1–S3 (variables such as study design, setting, time, region, age group, specimen type, pathogen, resistance phenotype, and data source characteristics).
Study risk of bias assessment	11	Specify the methods used to assess risk of bias in the included studies, including details of the tool(s) used, how many reviewers assessed each study and whether they worked independently, and if applicable, details of automation tools used in the process.	Methods -Section 3 ‘Systematic Review and Meta-analysis’ (risk-of-bias tools: AMR-adapted

Section and Topic	Item #	Checklist item	Location where item is reported
			Joanna Briggs Institute checklist for prevalence studies, ROBINS-I for non-randomised studies); Methods -Section 11.1 ‘Quality assessments and methodological validation’; Supplementary Table S4 (study-level risk-of-bias judgments and domains).
Effect measures	12	Specify for each outcome the effect measure(s) (e.g. risk ratio, mean difference) used in the synthesis or presentation of results.	Methods -Sections 4 ‘Statistical and Computational Analysis’ and 6 ‘Burden Estimation’ (effect measures: logit-transformed resistance proportions, rates, and AMR-attributable deaths/DALYs with 95% uncertainty intervals); Results -Sections 2–3 ‘National Burden of AMR’ and ‘Pathogen and Syndrome-Specific Burden’.
Synthesis methods	13a	Describe the processes used to decide which studies were eligible for each synthesis (e.g. tabulating the study intervention characteristics and comparing against the planned groups for each synthesis (item #5)).	Methods -Sections 3 ‘Systematic Review and Meta-analysis’ and 4.1–4.2 (grouping of studies by pathogen–drug phenotype, clinical syndrome, age group, region, and period; handling of overlapping cohorts); Supplementary Tables S3 and S7 (mapping of studies to pathogen–drug syntheses and meta-analytic summaries).

PRISMA

PRISMA 2020 Checklist

Section and Topic	Item #	Checklist item	Location where item is reported
	13b	Describe any methods required to prepare the data for presentation or synthesis, such as handling of missing summary statistics, or data conversions.	Methods- Sections 4.1 ‘Data Integration and Management’, 4.2 ‘Handling Overlapping and Duplicate Data’, 4.3 ‘Spatiotemporal Data Processing’, 4.4 ‘Missing Data Methodology’, and 11 ‘Antimicrobial Susceptibility Interpretation and Quality Control Framework’ (data cleaning, transformation, breakpoint harmonisation, imputation and preparation steps before synthesis).
	13c	Describe any methods used to tabulate or visually display results of individual studies and syntheses.	Methods – Section 4 “Statistical and Computational Analysis” and Sections 6–7 “Burden Estimation” and “Forecasting and Scenario-Based Modelling” (tabulation and visualisation strategies for study-level and modelled estimates); Results – Tables 1–2 and Figures 2–11; Supplementary Tables S5–S7 (tabulated estimates by pathogen, phenotype, region, and time).
	13d	Describe any methods used to synthesize results and provide a rationale for the choice(s). If meta-analysis was performed, describe the model(s), method(s) to identify the presence and extent of statistical heterogeneity, and software package(s) used.	Methods – Section 4 “Statistical and Computational Analysis” and Sections 6–7

Section and Topic	Item #	Checklist item	Location where item is reported
			"Burden Estimation" and "Forecasting and Scenario-Based Modelling" (random-effects meta-analysis, Bayesian hierarchical models, and scenario projections; rationale for model choice, covariates and heterogeneity measures); Results – Sections 2–3 "National Burden of AMR" and "Pathogen and Syndrome-Specific Burden", and Section 6 "Projections to 2050".
	13e	Describe any methods used to explore possible causes of heterogeneity among study results (e.g. subgroup analysis, meta-regression).	Methods -Sections 4.3 'Spatiotemporal Data Processing', 5 'Geospatial and Regional Analytical Methods', and 4.1 'Data Integration and Management' (meta-regression and spatial models exploring heterogeneity by region, healthcare access and demographics); Results - Sections 1.3, 2.1, 2.3 and 5 ('Regional and health system tier representativeness', 'Temporal and Demographic Patterns', 'Isolate distribution by pathogen, region, and period', and 'Regional distribution patterns').
	13f	Describe any sensitivity analyses conducted to assess robustness of the synthesized results.	Methods-Sections 4.4 'Missing Data Methodology', 6

Section and Topic	Item #	Checklist item	Location where item is reported
			‘Burden Estimation’ and 7 ‘Forecasting and Scenario-Based Modelling’; Supplementary Information -Section 4 ‘Validation, Diagnostics and Sensitivity’ (MNAR assumptions, tipping-point and scenario sensitivity analyses); Results -Section 6 ‘Projections to 2050’ and associated sensitivity analysis paragraph in the main text.
Reporting bias assessment	14	Describe any methods used to assess risk of bias due to missing results in a synthesis (arising from reporting biases).	Methods -Sections 4.4 ‘Missing Data Methodology’ and 4 ‘Statistical and Computational Analysis’ (use of imputation, hierarchical models and publication-bias diagnostics); Supplementary Table S7 and Supplementary Section 4 ‘Validation, Diagnostics and Sensitivity’ (Peters test and small-study effect diagnostics for key pathogen–drug syntheses).
Certainty assessment	15	Describe any methods used to assess certainty (or confidence) in the body of evidence for an outcome.	No formal GRADE-style certainty-of-evidence framework was applied; overall certainty and uncertainty are addressed narratively in Methods -Sections 11.1 ‘Quality assessments and

PRISMA 2020 Checklist

Section and Topic	Item #	Checklist item	Location where item is reported
			methodological validation' and 6–8 (propagation of uncertainty in burden estimates) and in Discussion -final paragraph describing limitations and uncertainty in the evidence base.
**RESULTS**			
Study selection	16a	Describe the results of the search and selection process, from the number of records identified in the search to the number of studies included in the review, ideally using a flow diagram.	Results -Section 1 'Study selection and characteristics of included studies' (description of records identified, screened, eligible and included); Figure 1 (PRISMA-2020 study selection flow diagram).
	16b	Cite studies that might appear to meet the inclusion criteria, but which were excluded, and explain why they were excluded.	Supplementary Information -Table S3 'Screening and exclusion log' (studies that appeared eligible but were excluded and reasons); Methods -Section 3 'Systematic Review and Meta-analysis' (screening and eligibility criteria).
Study characteristics	17	Cite each included study and present its characteristics.	Results -Section 1.1 'Data properties and geographical extent', 1.2 'Temporal trends and conflict disruption', and 1.3 'Regional and health system tier representativeness'; Table 1 (regional and facility-level

Section and Topic	Item #	Checklist item	Location where item is reported
			representation of the 2024 surveillance dataset); Supplementary Tables S1, S4–S6 (study characteristics, data source types and coverage).
Risk of bias in studies	18	Present assessments of risk of bias for each included study.	Results -mention of study-level risk-of-bias in Section 1.5 ‘Meta-analytic synthesis reporting (heterogeneity)’; Supplementary Table S4 (risk-of-bias assessments for each included observational study) and Supplementary Section 4 ‘Validation, Diagnostics and Sensitivity’.
Results of individual studies	19	For all outcomes, present, for each study: (a) summary statistics for each group (where appropriate) and (b) an effect estimates and its precision (e.g. confidence/credible interval), ideally using structured tables or plots.	Results -Section 2.3 ‘Isolate distribution by pathogen, region, and period’, Section 3 ‘Pathogen and Syndrome-Specific Burden’, and Section 4 ‘Molecular Epidemiology’; Table 2 and supplementary tables (study-level or source-level estimates and summary statistics by pathogen–drug–region combinations).
Results of syntheses	20a	For each synthesis, briefly summarise the characteristics and risk of bias among contributing studies.	Results -Sections 2 and 3 (‘National Burden of AMR (1970–2024)’ and ‘Pathogen and Syndrome-Specific Burden’) and Section 6 ‘Projections to 2050’

Section and Topic	Item #	Checklist item	Location where item is reported
			(summary of each synthesis and its contributing evidence); Supplementary Table S7 (pooled resistance estimates, heterogeneity statistics and small-study effect diagnostics for each pathogen–phenotype synthesis).
	20b	Present results of all statistical syntheses conducted. If meta-analysis was done, present for each the summary estimate and its precision (e.g. confidence/credible interval) and measures of statistical heterogeneity. If comparing groups, describe the direction of the effect.	Results -Sections 2–3 and 6 (meta-analytic outputs, model-based estimates and projections with 95% uncertainty intervals, including summary tables and Figures 4–10); Methods -Section 6 'Burden Estimation' (details of counterfactual modelling and effect metrics).
	20c	Present results of all investigations of possible causes of heterogeneity among study results.	Results -Section 1.5 'Meta-analytic synthesis reporting (heterogeneity)' and Section 5 'Regional distribution patterns' (findings from heterogeneity analyses across settings and regions); Supplementary Table S7 and Supplementary Section 4 'Validation, Diagnostics and Sensitivity' (meta-regression, spatial heterogeneity and small-study effect analyses).
	20d	Present results of all sensitivity analyses conducted to assess the robustness of the synthesized results.	Results -Section 6 'Projections to 2050' (scenario-based

Section and Topic	Item #	Checklist item	Location where item is reported
			sensitivity analyses for future AMR burden); Discussion -paragraph beginning ‘Sensitivity analysis suggested that…’; Supplementary Section 4 ‘Validation, Diagnostics and Sensitivity’.
Reporting biases	21	Present assessments of risk of bias due to missing results (arising from reporting biases) for each synthesis assessed.	Results -Section 1.5 ‘Meta-analytic synthesis reporting (heterogeneity)’ and Supplementary Section 4 ‘Validation, Diagnostics and Sensitivity’ (publication bias and small-study effect diagnostics, including Peters test) and Supplementary Table S7.
Certainty of evidence	22	Present assessments of certainty (or confidence) in the body of evidence for each outcome assessed.	No formal quantitative certainty-of-evidence grading (e.g. GRADE) was performed; overall certainty is discussed qualitatively in Methods -Sections 11.1 ‘Quality assessments and methodological validation’ and 6–8, and in Discussion -limitations paragraph describing data gaps, bias risks and uncertainty in estimates.
**DISCUSSION**			
Discussion	23a	Provide a general interpretation of the results in the context of other evidence.	Discussion -first and second paragraphs (overall interpretation of AMR burden in Libya in the context of previous

PRISMA 2020 Checklist

Section and Topic	Item #	Checklist item	Location where item is reported
			Libyan reports and regional/global estimates).
	23b	Discuss any limitations of the evidence included in the review.	Discussion -paragraph beginning 'Despite integrating national and multiple data sources…' and related text discussing strengths and limitations of the underlying evidence (e.g. urban bias, incomplete surveillance, limited molecular data).
	23c	Discuss any limitations of the review processes used.	Discussion -limitations paragraph (constraints of the multi-source review and modelling framework, assumptions regarding missing data, breakpoint harmonisation and surveillance coverage); Supplementary Section 4 'Validation, Diagnostics and Sensitivity'.
	23d	Discuss implications of the results for practice, policy, and future research.	Discussion -final paragraphs (implications for stewardship, diagnostics, surveillance and policy in Libya and neighbouring countries; recommendations for future research and capacity-building).
**OTHER INFORMATION**			
Registration and protocol	24a	Provide registration information for the review, including register name and registration number, or state that the review was not registered.	Methods -Section 3 'Systematic Review and Meta-analysis' (statement that the review protocol was preregistered in

PRISMA 2020 Checklist

Section and Topic	Item #	Checklist item	Location where item is reported
			PROSPERO, with registry ID CRD420251066018).
	24b	Indicate where the review protocol can be accessed, or state that a protocol was not prepared.	Methods -Section 3 ‘Systematic Review and Meta-analysis’ (PROSPERO registration number CRD420251066018, protocol details); Supplementary Information -protocol description in the PROSPERO registry and summarised in the Supplement.
	24c	Describe and explain any amendments to information provided at registration or in the protocol.	Not applicable -no amendments to the preregistered PROSPERO protocol were made; any minor clarifications are described within the Methods and Discussion sections.
Support	25	Describe sources of financial or non-financial support for the review, and the role of the funders or sponsors in the review.	Funding statement - ‘Funding’ subsection in the Declarations (no external funding; roles of institutions and contributors); Supplementary Information -Section 6 ‘Funding, Roles, and Conflicts’.
Competing interests	26	Declare any competing interests of review authors.	‘Competing interests’ subsection in the Declarations (authors declare no competing interests); Supplementary Information -Section 6

PRISMA

PRISMA 2020 Checklist

Section and Topic	Item #	Checklist item	Location where item is reported
			'Funding, Roles, and Conflicts'.
Availability of data, code and other materials	27	Report which of the following are publicly available and where they can be found: template data collection forms; data extracted from included studies; data used for all analyses; analytic code; any other materials used in the review.	'Data Availability' subsection in the Declarations (access to aggregated and de-identified data from hospitals, laboratories and pharmacies); Supplementary Information -Section 5 'Data and Code Availability' (planned deposition of de-identified extraction sheets, PRISMA logs and analysis code in an open repository).

From: Page MJ, McKenzie JE, Bossuyt PM, Boutron I, Hoffmann TC, Mulrow CD, et al. The PRISMA 2020 statement: an updated guideline for reporting systematic reviews. BMJ 2021;372:n71. doi: 10.1136/bmj.n71. This work is licensed under CC BY 4.0. To view a copy of this license, visit https://creativecommons.org/licenses/by/4.0/
