# Supplementary material for: Burden of Bacterial Antimicrobial Resistance in Libya, 1970–2024: A Systematic Meta-Analysis with Projections to 2050
Source: Antibiotics (Basel). 2026 Jan 16;15(1):92. doi: 10.3390/antibiotics15010092 (PMC12837905; doi:10.3390/antibiotics15010092)
Supplement: Supplementary file 1 [file antibiotics-15-00092-s001.zip › Supplementary Information_V9.pdf]

Supplementary Tables

Table S1. Data sources matrix and hierarchical data structure for integration. Temporal coverage standardized to 1 January 1970 to 31 December 2024. International/Regional repositories (e.g., WHO GLASS, WHO/EMRO–IMEMR; AJOL) were used descriptively where applicable; pooled quantitative analyses were derived from the published literature set only. Abbreviations: GLASS-Global Antimicrobial Resistance and Use Surveillance System; IMEMR-Index Medicus for the Eastern Mediterranean; AJOL-African Journals Online; NCDC-National Center for Disease Control (Libya).

Data Source Category	Source Name	Temporal Coverage (start–end)	Geographic Scope / Level	Extracted Parameters (examples)	Acquisition Protocol (URL / contact / script)	Methodological Limitations / Notes
International surveillance	WHO GLASS (Global Antimicrobial Resistance and Use Surveillance System)	2018–2024 (as available)	National	Isolates tested; organism–drug pairs; S/I/R counts; specimen type	GLASS portal via WHO/EMRO; CSV exports; internal logs	Reporting completeness varies by year and participating sites
Regional repository	WHO/EMRO repositories (e.g., IMEMR, EMRO AMR portals)	1 January 1970 – 31 December 2024	Regional (EMRO)	Aggregated resistance proportions; Libya-tagged records; bibliographic metadata	EMRO portals; manual screening; export where possible	Heterogeneous methods; variable metadata and indexing
National surveillance	NCDC–Libya (National Center for Disease Control)	2014–2024	National / Regional	Organism–drug counts; specimen types; facility/region	Formal data requests; annual reports; manual extraction	Variable site participation; intermittent reporting
Hospital networks	Benghazi Medical Center; Al-Jalaa; Al-Kuwefia; other tertiary facilities	2000–2024	Facility / City	Laboratory AST results; breakpoints; QC indicators; study periods	LIMS/CSV exports; site PIs; manual curation	Breakpoint versions differ across years; QC documentation sometimes incomplete
Published literature	Systematic review of Libya AMR studies (included n = 30)	1 January 1970 – 31 December 2024	Study-level	n tested; n resistant (per organism–drug); AST method; breakpoint	PRISMA 2020 screening logs and extraction sheets	Selection/reporting biases; denominators sometimes inconsistent

				version		

Table S2. Search sources, coverage window, and PRISMA totals. Literature databases searched and standardized coverage window for the systematic review. PRISMA 2020 totals reflect database records only and align with Supplementary Figure S1. The coverage window is 1 January 1970 to 31 December 2024. Abbreviations: PRISMA-Preferred Reporting Items for Systematic Reviews and Meta-Analyses; IMEMR-Index Medicus for the Eastern Mediterranean; AJOL-African Journals Online.

A. Literature databases and coverage window

**Source**	**Coverage window**
PubMed/MEDLINE	1 January 1970 – 31 December 2024
EMBASE	1 January 1970 – 31 December 2024
Web of Science	1 January 1970 – 31 December 2024
Scopus	1 January 1970 – 31 December 2024
CINAHL	1 January 1970 – 31 December 2024
Cochrane Library	1 January 1970 – 31 December 2024
Global Index Medicus (IMEMR)	1 January 1970 – 31 December 2024
African Journals Online (AJOL)	1 January 1970 – 31 December 2024

B. PRISMA 2020 totals (databases only)

**Metric**	**Count**
Records identified (databases)	342
Duplicate records removed	106
Records after de-duplication (screened at title/abstract)	236
Records excluded at title/abstract	122
Full-text articles assessed for eligibility	114
Full-text articles excluded with reasons	52
Studies included (qualitative & quantitative synthesis)	62

Table S3. Full-text articles excluded with primary reasons (PRISMA 2020). Caption: Keyword rules for assignment (author to confirm): [1] Year ≥ 2025 (Outside coverage) window; [2] Animal/environmental/food/retail terms (Wrong sample frame); [3] Review/editorial/meta-analysis (Wrong study design); [4] In vitro / plant extracts / antibacterial activity only (Wrong outcome); [5] Country terms without 'Libya' (Libya stratum not extractable); [6] No AST-related terms (No extractable AMR data); otherwise (Insufficient methods/reporting).

**Record ID**	**Full citation (verbatim from user's file)**	**Year**	**PRIMARY reason (PRISMA category)**	**Reason confidence**	**Notes (≤1 line)**
EX01	Adalla, S. A., & Garmole, B. (1996). Tuberculous parotitis: two cases in Libyan patients. The British journal of clinical practice, 50(1), 62–63.	1996	No extractable AMR data/denominator (unclear AST reporting)	Low	
EX02	Aishbani, S. J., Al-Griw, H. H., Al-Sharif, J. A., Karim, E. S., Farag, S.	2025	Outside coverage window	High	

	A., Ahmed, M. O., & Abouzeed, Y. M. (2025). Antibiotic susceptibility of Salmonella spp isolated from farm animals and environmental sources in Tripoli, Libya. Open veterinary journal, 15(5), 1941–1946.		(post-2024)		
EX03	Alghazeer, R. O., Azwai, S. M., Elmansori, A. A., Alzintani, K. M., Elghmasi, S., Alnajjar, A. Y., Gammoudi, F. T., Sidati, M., Hawisa, S. T., Garbaj, A. M., & Eldaghayes, I. M. (2024). Antibacterial activity of flavonoid extracts from Enteromorpha intestinalis and Caulerpa prolifera against multidrug-resistant foodborne bacterial isolates. Open veterinary journal, 14(3), 769–778.	2024	Wrong outcome (no clinical AST; in vitro activity)	High	
EX04	Ali, M. B., Ghenghesh, K. S., Aissa, R. B., Abuhelfaia, A., & Dufani, M. (2005). Etiology of childhood diarrhea in Zliten, Libya. Saudi medical journal, 26(11), 1759–1765.	2005	No extractable AMR data/denominator (unclear AST reporting)	Low	
EX05	Ali, M. M., Aburowes, A. H., Albakush, A. M., Rzeg, M. M., Alrtail, A., & Ghenghesh, K. S. (2014). Identification of multidrug-resistant bacteria and Bacillus cereus from healthcare workers and environmental surfaces in a hospital. The Libyan journal of medicine, 9(1), 25794. Alkarghali AF, Mohammed SH, Miftah RK, abdulsamad Na, Elbasha MS, Doukali AS,	2014	No extractable AMR data/denominator (unclear AST reporting)	Low	
EX06	Altayr, N. A., Bukha, K. K., Eteer, S. A., El-Magrahi, N. A., Elhom, A. M., Elmaloul, R. A., & Fakar, S. F. (2025). A comparative study to investigate foodborne pathogenic bacteria in fresh and imported frozen Scomber japonicus (Houttuyn, 1782) in Tripoli, Libya. Open veterinary journal, 15(4), 1607–1614.	2025	Outside coverage window (post-2024)	High	
EX07	Aloriby, M., Elkawafi, M., Aldrsy, S., Sweker, M., Elabdeli, H., Elbarghathi, A., Benhasouna, A., El-Awamie, M., Elsharif, N., Alqabbasi, O., Alshalmani, S., Algazal, R., & Bleiblo, F. (2025). Overall in vitro, in vivo, and in silico evaluation of Olea europaea and Ficus carica leaf extracts for antimicrobial activity against multidrug-resistant	2025	Outside coverage window (post-2024)	High	

	pathogens. Frontiers in microbiology, 16, 1567921.				
EX08	Alwashsiah, M. M., & Abbas, A. A. (2025). Prevalence and characterization of β-lactamase-producing bacteria in gingivitis among diabetic and non-diabetic patients: a comparative microbiological study. Access microbiology, 7(10), 001094.v3.	2025	Outside coverage window (post-2024)	High	
EX09	Baaiu, B. S., Saleh, N. M., Alshref Aldirsi, A. F., & Abdel-Aziem, A. (2025). Synthesis of new coumarin derivatives and assessment of their antimicrobial efficacy. Future medicinal chemistry, 17(1), 9–18.	2025	Outside coverage window (post-2024)	High	
EX10	Dakheel, M. A., Najaim, A. M., Moslah, A. M., & Buni, H. D. (2021). The Disseminated tuberculosis with cavitary lung lesion and tuberculoma in a six-month-old Libyan infant. Journal of infection in developing countries, 15(12), 1929–1932.	2021	No extractable AMR data/denominator (unclear AST reporting)	Low	
EX11	Daw, M. A., El-Bouzedi, A. H., Abumahara, S. A., Najjar, A. K., Ben Ashur, N. R., Grebi, A., Dhu, A. M., Alkarghali, A. F., Mohammed, S. H., Miftah, R. K., Abdulsamad, N. A., Elbasha, M. S., Doukali, A. S., Elmhidwi, N. T., Albouzaidi, E. O., Wareg, S. E., & Ahmed, M. O. (2025). Geographic mapping and spatiotemporal patterns of tuberculosis in Libya within ten years' period (2015 to 2024). Frontiers in epidemiology, 5, 1571065.	2025	Outside coverage window (post-2024)	High	
EX12	Elgderi, R. M., Ghenghesh, K. S., & Berbash, N. (2006). Carriage by the German cockroach (Blattella germanica) of multiple-antibiotic-resistant bacteria that are potentially pathogenic to humans, in hospitals and households in Tripoli, Libya. Annals of tropical medicine and parasitology, 100(1), 55–62.	2006	No extractable AMR data/denominator (unclear AST reporting)	Low	
EX13	Ellabib, M. S., & ElJariny, I. A. (2001). In vitro activity of 6 antifungal agents on candida species isolated as causative agents from vaginal and other clinical specimens. Saudi medical journal, 22(10), 860–863.	2001	Wrong outcome (no clinical AST; in vitro activity)	High	
EX14	Elmhidwi NT, Albouzaidi EO, Wareg	2025	Outside coverage	High	

	SE and Ahmed MO (2025) Geographic mapping and spatiotemporal patterns of tuberculosis in Libya within ten years' period (2015 to 2024). Front. Epidemiol. 5:1571065. doi: 10.3389/fepid.2025.1571065.		window (post-2024)		
EX15	Elnageh, H. R., Hiblu, M., Abbassi, M. S., Abouzeed, Y. M., & Ahmed, M. O. (2021). Prevalence and antimicrobial resistance of Salmonella serotypes isolated from cats and dogs in Tripoli, Libya. Veterinaria italiana, 57(2), 10.12834/VetIt.1998.10744.4.	2021	Insufficient methods/reporting for AMR extraction	Low	
EX16	EMR Antimicrobial Resistance Collaborators (2025). The burden of bacterial antimicrobial resistance in the WHO Eastern Mediterranean Region 1990-2021: a cross-country systematic analysis with forecasts to 2050. The Lancet. Public health, 10(11), e955–e970.	2025	Outside coverage window (post-2024)	High	
EX17	Fossati C. (1963). Incidenza Della Tubercolosi Polmonare E Di Altre Malattie Dell'apparato Respiratorio Nella Popolazione Araba Della Cirenaica Durante Il Biennio Luglio 1959-Luglio 1961 [Incidence Of Pulmonary Tuberculosis And Other Diseases Of The Respiratory System In The Arab Population Of Cyrenaica During The 2-Year Period From July 1959 To July 1961]. Lotta Contro La Tubercolosi, 33, 416–437.	1963	No extractable AMR data/denominator (unclear AST reporting)	Low	
EX18	Fossati C. (1963). Infecci'on Y Mortalidad Tuberculosa Descubierta En Las Colectividadades Supuestas Sanas En El Dispensario De V'ias Respiratorias De La Ciudad De Bengasis (Libia) En Los A Nos De 1958 A 1963 Inclusive [Infection And Tuberculosis Mortality Discovered In The Supposedly Healthy Collectivities At The Respiratory Tract Dispensary In The City Of Bengasis (Libya) In The Years Of 1958 To 1963 Inclusive]. Revista Espanola De Tuberculosis, 32, 475–488.	1963	No extractable AMR data/denominator (unclear AST reporting)	Low	
EX19	Fossati C. (1964). Quimioprofilaxis Antituberculosa Con Isoniacida Seg'un El M'etodo De Omodei-Zorini En Un Grupo De Ni Nos Arebes-	1964	No extractable AMR data/denominator (unclear AST	Low	

	L'ibicos De La Cirenaica Expuestos Al Contagio Tuberculoso Familiar [Antituberculous Chemoprophylaxis With Isoniazid According To The Omodei-Zorini Method In A Group Of Arab Libyan Children Of Cyreaica Exposed To Familial Tuberculosis Infection]. Revista Espanola De Tuberculosis, 33, 299–314.		reporting)		
EX20	Franka, E. A., Shembesh, M. K., Zaied, A. A., El-Turki, E., Zorgani, A., Elahmer, O. R., & Ghenghesh, K. S. (2012). Multidrug resistant bacteria in wounds of combatants of the Libyan uprising. The Journal of infection, 65(3), 279–281.	2012	No extractable AMR data/denominator (unclear AST reporting)	Low	
EX21	Gammo, M., Lamaric, W., Hadida, M., Abuazza, A., Askar, N. A., Yassin, M. A., & Cuevas, L. E. (2013). Front-loaded smear microscopy for the diagnosis of pulmonary TB in Tripoli, Libya. Transactions of the Royal Society of Tropical Medicine and Hygiene, 107(2), 137–139.	2013	No extractable AMR data/denominator (unclear AST reporting)	Low	
EX22	Garbaj, A. M., Gawella, T. B. B., Sherif, J. A., Naas, H. T., Eshamah, H. L., Azwai, S. M., Gammoudi, F. T., Abolghait, S. K., Moawad, A. A., Barbieri, I., & Eldaghayes, I. M. (2022). Occurrence and antibiogram of multidrug-resistant Salmonella enterica isolated from dairy products in Libya. Veterinary world, 15(5), 1185–1190.	2022	Insufficient methods/reporting for AMR extraction	Low	
EX23	Ghenghesh, K. S., Ahmed, S. F., El-Khalek, R. A., Al-Gendy, A., & Klena, J. (2008). Aeromonas-associated infections in developing countries. Journal of infection in developing countries, 2(2), 81–98.	2008	No extractable AMR data/denominator (unclear AST reporting)	Low	
EX24	Ghenghesh, K. S., El-Ghodban, A., Dkakni, R., Abeid, S., Altomi, A., Abdussalam, T., & Marialigeti, K. (2001). Prevalence, species differentiation, haemolytic activity, and antibiotic susceptibility of aeromonads in untreated well water. Memorias do Instituto Oswaldo Cruz, 96(2), 169–173.	2001	No extractable AMR data/denominator (unclear AST reporting)	Low	
EX25	Ghenghesh, K. S., El-Mohammady, H., Levin, S. Y., Zorgani, A., & Tawil, K. (2013). Antimicrobial resistance profile of Aeromonas	2013	Insufficient methods/reporting for AMR extraction	Low	

	species isolated from Libya. The Libyan journal of medicine, 8(1), 21320.				
EX26	Goda F. F. (1976). Antibiotic sensitivity of Staphylococcus aureus isolated from mastitis in cows at Benghazi localities. Bulletin of animal health and production in Africa. Bulletin des sante et production animales en Afrique, 24(1), 47–52.	1976	Wrong sample frame (non-human clinical / environmental)	High	
EX27	Khalifa, M. E., & Omar, S. M. (2025). Characterisation of phage vB_Ec_DUEC01: A lytic Kagunavirus for multidrug-resistant Escherichia coli. Microbial pathogenesis, 209, 108124.	2025	Outside coverage window (post-2024)	High	
EX28	Khalil, A., & Sathianathan, S. (1978). Impact of anti-tuberculosis legislation in Libya on the prevalence of primary and acquired resistance to the three main drugs at a majortuberculosis centre. Tubercle, 59(1), 1–12.	1978	Insufficient methods/reporting for AMR extraction	Low	
EX29	Kieffer, N., Ahmed, M. O., Elramalli, A. K., Daw, M. A., Poirel, L., Álvarez, R., & Nordmann, P. (2018). Colistin-resistant carbapenemase-producing isolates among Klebsiella spp. and Acinetobacter baumannii in Tripoli, Libya. Journal of global antimicrobial resistance, 13, 37–39.	2018	Insufficient methods/reporting for AMR extraction	Low	
EX30	Kissopoulos A. (1963). Stato Epidemiologico Attuale Della Tbc In Tripolitania. Rilevamento Su 20,000 Casi Di Tbc Al Primo Accertamento Presso Il Dispensario Antitubercolare Di Tripoli (Libia) [Current Epidemiological Status Of Tuberculosis In Tripolitania. Report On 20,000 Cases Of Tuberculosis Verified For The First Time At The Dispensario Antitubercolare Di Tripoli (Libya)]. Lotta Contro La Tubercolosi, 33, 388–403.	1963	No extractable AMR data/denominator (unclear AST reporting)	Low	
EX31	Kocsis, E., Savio, C., Piccoli, M., Cornaglia, G., & Mazzariol, A. (2013). Klebsiella pneumoniae harbouring OXA-48 carbapenemase in a Libyan refugee in Italy. Clinical microbiology and infection : the official publication of the European Society of Clinical Microbiology and Infectious Diseases, 19(9), E409–E411.	2013	No extractable AMR data/denominator (unclear AST reporting)	Low	

EX32	Koole, K., Ellerbroek, P. M., Lagendijk, R., Leenen, L. P., & Ekkelenkamp, M. B. (2013). Colonization of Libyan civil war casualties with multidrug-resistant bacteria. Clinical microbiology and infection : the official publication of the European Society of Clinical Microbiology and Infectious Diseases, 19(7), E285–E287.	2013	No extractable AMR data/denominator (unclear AST reporting)	Low	
EX33	Miller, L. N., Elmselati, H., Fogarty, A. S., Farhat, M. E., Standley, C. J., Abuabaid, H. M., Zorgani, A., Elahmer, O., & Sorrell, E. M. (2023). Using One Health assessments to leverage endemic disease frameworks for emerging zoonotic disease threats in Libya. PLOS global public health, 3(7), e0002005.	2023	No extractable AMR data/denominator (unclear AST reporting)	Low	
EX34	Mohamed Ali, M. M., Alemary, F., Alrtail, A., Rzeg, M. M., Albakush, A. M., & Ghenghesh, K. S. (2014). High isolation rates of multidrug-resistant bacteria from water and carpets of mosques. The Libyan journal of medicine, 9(1), 25415.	2014	No extractable AMR data/denominator (unclear AST reporting)	Low	
EX35	Naas, H. T., Edarhoby, R. A., Garbaj, A. M., Azwai, S. M., Abolghait, S. K., Gammoudi, F. T., Moawad, A. A., Barbieri, I., & Eldaghayes, I. M. (2019). Occurrence, characterization, and antibiogram of Staphylococcus aureus in meat, meat products, and some seafood from Libyan retail markets. Veterinary world, 12(6), 925–931.	2019	Wrong sample frame (non-human clinical / environmental)	High	
EX36	Obeid, M. A., Alyamani, H., Alenaizat, A., Tunç, T., Aljabali, A. A. A., & Alsaadi, M. M. (2025). Nanomaterial-based drug delivery systems in overcoming bacterial resistance: Current review. Microbial pathogenesis, 203, 107455.	2025	Outside coverage window (post-2024)	High	
EX37	Ouertani, R., Limelette, A., Guillard, T., Brasme, L., Jridi, Y., Barguellil, F., El Salabi, A., de Champs, C., & Chouchani, C. (2016). First report of nosocomial infection caused by Klebsiella pneumoniae ST147 producing OXA-48 and VEB-8 β-lactamases in Tunisia. Journal of global antimicrobial resistance, 4, 53–56.	2016	Libya stratum not extractable / wrong country	Medium	
EX38	Othman, A. A., Hiblu, M. A.,	2021	Insufficient	Low	

	Abbassi, M. S., Abouzeed, Y. M., & Ahmed, M. O. (2021). Nasal colonization and antibiotic resistance patterns of Staphylococcus species isolated from healthy horses in Tripoli, Libya. Journal of equine science, 32(2), 61–65.		methods/reporting for AMR extraction		
EX39	Patel, J., Moghaddam, S. S., Ranganathan, S., Vezeau, N., O'Neill, E., Harant, A., Stolpe, M., Wieler, L. H., Eckmanns, T., & Sridhar, D. (2025). Global policy responses to antimicrobial resistance, 2021-22: a systematic governance analysis of 161 countries and territories. The Lancet. Infectious diseases, S1473-3099(25)00406-2. Advance online publication.	2025	Outside coverage window (post-2024)	High	
EX40	Rahouma, A., Elghamoudi, A., Nashnoush, H., Belhaj, K., Tawil, K., & Sifaw Ghenghesh, K. (2010). Isolation of antibiotic-resistant pathogenic and potentially pathogenic bacteria from carpets of mosques in Tripoli, Libya. The Libyan journal of medicine, 5, 10.3402/ljm.v5i0.5536.	2010	No extractable AMR data/denominator (unclear AST reporting)	Low	
EX41	Rahuma, N., Ghenghesh, K. S., Ben Aissa, R., & Elamaari, A. (2005). Carriage by the housefly (Musca domestica) of multiple-antibiotic-resistant bacteria that are potentially pathogenic to humans, in hospital and other urban environments in Misurata, Libya. Annals of tropical medicine and parasitology, 99(8), 795–802.	2005	No extractable AMR data/denominator (unclear AST reporting)	Low	
EX42	Seiffert, S. N., Perreten, V., Johannes, S., Droz, S., Bodmer, T., & Endimiani, A. (2014). OXA-48 carbapenemase-producing Salmonella enterica serovar Kentucky isolate of sequence type 198 in a patient transferred from Libya to Switzerland. Antimicrobial agents and chemotherapy, 58(4), 2446–2449.	2014	No extractable AMR data/denominator (unclear AST reporting)	Low	
EX43	Shalaka N. (2024). Retrospective study of the prevalence of acquired drug resistance after failed antiretroviral therapy in Libya. Eastern Mediterranean health journal = La revue de sante de la Mediterranee orientale = al-Majallah al-sihhiyah li-sharq al-mutawassit, 30(7), 470–480.	2024	Insufficient methods/reporting for AMR extraction	Low	

EX44	Shambesh, M. K., Craig, P. S., Gusbi, A. M., Echtuish, E. F., & Wen, H. (1995). Immunoblot evaluation of the 100 and 130 kDa antigens in camel hydatid cyst fluid for the serodiagnosis of human cystic echinococcosis in Libya. Transactions of the Royal Society of Tropical Medicine and Hygiene, 89(3), 276–279.	1995	Wrong sample frame (non-human clinical / environmental)	High	
EX45	Slimene, K., Ali, A. A., Mohamed, E. A., El Salabi, A., Suliman, F. S., Elbadri, A. A., El-Fertas, F. F., El-Awjly, A., Shokri, S. A., Rolain, J. M., & Chouchani, C. (2023). Isolation of Carbapenem and Colistin Resistant Gram-Negative Bacteria Colonizing Immunocompromised SARS-CoV-2 Patients Admitted to Some Libyan Hospitals. Microbiology spectrum, 11(3), e0297222.	2023	No extractable AMR data/denominator (unclear AST reporting)	Low	
EX46	Slimene, K., Almahjoub, S. K., Alkeskas, A. A., Elbousify, A. I., Omar, E. O., El Salabi, A. A., Mohamed, E. A., Hameid, M. I., Alsanosi, S., Miniaoui, D., Rolain, J. M., & Chouchani, C. (2025). Molecular characterization and diversity of carbapenemases in Gram-negative bacteria in Libyan hospitals. Journal of infection in developing countries, 19(7), 1089–1099.	2025	Outside coverage window (post-2024)	High	
EX47	Stanford, J. L., Shield, M. J., Paul, R. C., Khalil, A., Tobgi, R. S., & Wallace, A. (1976). The effect of desert conditions on the reactivity of Libyan schoolchildren to a range of new tuberculins. The Journal of hygiene, 77(1), 63–75.	1976	No extractable AMR data/denominator (unclear AST reporting)	Low	
EX48	Sufya, N., Matar, N., Kaddura, R., & Zorgani, A. (2014). Evaluation of bactericidal activity of Hannon honey on slowly growing bacteria in the chemostat. Drug, healthcare and patient safety, 6, 139–144. https://doi.org/10.2147/DHPS.S66496	2014	No extractable AMR data/denominator (unclear AST reporting)	Low	
EX49	Taher, A. A., Rao, B. N., Alganay, K. G., & el-Arabi, M. B. (2000). An outbreak of acute gastroenteritis due to Aeromonas sobria in Benghazi, Libyan Arab Jamahiriya. Eastern Mediterranean health journal = La	2000	No extractable AMR data/denominator (unclear AST reporting)	Low	

	revue de sante de la Mediterranee orientale = al-Majallah al-sihhiyah li-sharq al-mutawassit, 6(2-3), 497–499.				
EX50	Wild, A., Shortall, C., Dewachi, O., Naim, C., Green, A., Hussain, S., & Abbara, A. (2025). Conflict-associated wounds and burns infected with GLASS pathogens in the Eastern Mediterranean Region: A systematic review. BMC infectious diseases, 25(1), 187.	2025	Outside coverage window (post-2024)	High	
EX51	Zorgani, A., & Ziglam, H. (2013). Injured Libyan combatant patients: both vectors and victims of multiresistance bacteria? The Libyan journal of medicine, 8, 10.3402/ljm.v8i0.20325.	2013	No extractable AMR data/denominator (unclear AST reporting)	Low	
EX52	Zorgani, A. A., Shahen, A., Zaidi, M., & Franka, M. (2006). A profile and spectrum of four cases of methicillin-resistant Staphylococcus aureus in a burns intensive care unit. Annals of burns and fire disasters, 19(1), 5–10.	2006	No extractable AMR data/denominator (unclear AST reporting)	Low	

Table S4. Risk of bias (AMR-adapted JBI) for included studies. AMR-adapted JBI domains (indicative). D1: Sampling frame representativeness; D2: Participant selection; D3: Case definition/diagnostic consistency; D4: Data source reliability; D5: AST methodology adherence (CLSI/EUCAST); D6: Denominator clarity; D7: Handling of duplicates/overlaps; D8: Missing data and reporting completeness; D9: Confounding/stratification; D10: Temporal/geographic clarity. Overall RoB is a study-level judgment synthesized from D1–D10.

IN ID	Full citation (verbatim)	Year	D1	D2	D3	D4	D5	D6	D7	D8	D9	D10	Overall RoB
IN01	Abou Fayad, A. G., Haraoui, L. P., Sleiman, A., Jaafar, M., Zorgani, A., Matar, G. M., & Higgins, P. G. (2021). Diversity of Sequence Types and Impact of Fitness Cost among Carbapenem-Resistant Acinetobacter baumannii Isolates from Tripoli, Libya. Antimicrobial agents and chemotherapy, 65(8), e0027721.	2021	NR	NR	NR	NR	NR	NR	NR	NR	NR	NR	NR
IN02	Abujnah, A. A., Zorgani, A., Sabri, M. A., El-Mohammady, H., Khalek, R. A., & Ghenghesh, K. S. (2015). Multidrug resistance and extended-spectrum β-lactamases genes	2015	NR	NR	NR	NR	NR	NR	NR	NR	NR	NR	NR

	among Escherichia coli from patients with urinary tract infections in Northwestern Libya. The Libyan journal of medicine, 10(1), 26412.												
IN 03	Ahmed, M. O., Abuzweda, A. R., Alghazali, M. H., Elramalli, A. K., Amri, S. G., Aghila, E. S.h, & Abouzeed, Y. M. (2010). Misidentification of methicillin-resistant Staphylococcus aureus (MRSA) in hospitals in Tripoli, Libya. The Libyan journal of medicine, 5, 10.3402/ljm.v5i0.5230.	20 10	N R	N R	N R	N R	N R	N R	N R	N R	N R	N R	NR
IN 04	Ahmed, M. O., Baptiste, K. E., Daw, M. A., Elramalli, A. K., Abouzeed, Y. M., & Petersen, A. (2017). Spa typing and identification of pvl genes of methicillin-resistant Staphylococcus aureus isolated from a Libyan hospital in Tripoli. Journal of global antimicrobial resistance, 10, 179–181.	20 17	N R	N R	N R	N R	N R	N R	N R	N R	N R	N R	NR
IN 05	Ahmed, M. O., Elramalli, A. K., Amri, S. G., Abuzweda, A. R., & Abouzeed, Y. M. (2012). Isolation and screening of methicillin-resistant Staphylococcus aureus from health care workers in Libyan hospitals. Eastern Mediterranean health journal = La revue de sante de la Mediterranee orientale = al-Majallah al-sihhiyah li-sharq al-mutawassit, 18(1), 37–42.	20 12	N R	N R	N R	N R	N R	N R	N R	N R	N R	N R	NR
IN 06	Ahmed, M. O., Elramalli, A. K., Baptiste, K. E., Daw, M. A., Zorgani, A., Brouwer, E., Willems, R. J. L., & Top, J. (2020). Whole Genome Sequence Analysis of the First Vancomycin-Resistant Enterococcus faecium Isolates from a Libyan Hospital in Tripoli. Microbial drug resistance (Larchmont, N.Y.), 26(11), 1390–1398.	20 20	N R	N R	N R	N R	N R	N R	N R	N R	N R	N R	NR
IN 07	Al-haddad, O. H., Zorgani, A., & Ghenghesh, K. S. (2014). Nasal carriage of multi-drug-resistant	20 14	N R	N R	N R	N R	N R	N R	N R	N R	N R	N R	NR

	Panton-Valentine leucocidin-positive methicillin-resistant Staphylococcus aureus in children in Tripoli-Libya. The American journal of tropical medicine and hygiene, 90(4), 724–727.												
IN 08	Al-Orphaly, M., Hadi, H. A., Eltayeb, F. K., Al-Hail, H., Samuel, B. G., Sultan, A. A., & Skariah, S. (2021). Epidemiology of Multidrug-Resistant Pseudomonas aeruginosa in the Middle East and North Africa Region. mSphere, 6(3), e00202-21.	20 21	N R	N R	N R	N R	N R	N R	N R	N R	N R	N R	NR
IN 09	Al-Shami, H. A., Abubakar, U., Hussein, M. S. E., Hussin, H. F. A., & Al-Shami, S. A. (2023). Awareness, practices and perceptions of community pharmacists towards antimicrobial resistance and antimicrobial stewardship in Libya: a cross-sectional study. Journal of pharmaceutical policy and practice, 16(1), 46.	20 23	N R	N R	N R	N R	N R	N R	N R	N R	N R	N R	NR
IN 10	Alawami, A. A., Al Haji, H., Hamied, M. I. K., Chouchani, C., & El Salabi, A. A. (2022). Letter to the Editor: New Treatment Options of Blood Stream Infections Caused by Multidrug-Resistant Acinetobacter baumannii in Some Libyan Patients. Microbial drug resistance (Larchmont, N.Y.), 28(1), 99–101.	20 22	N R	N R	N R	N R	N R	N R	N R	N R	N R	N R	NR
IN 11	Atia A, Hosien B, Belhaj H. Antimicrobial resistance in Libya: A systematic literature review of two decades. Biomed Biotechnol Res J 2022;6:473-82.	20 22	N R	N R	N R	N R	N R	N R	N R	N R	N R	N R	NR
IN 12	Balakrishnan V. S. (2022). The burden of tuberculosis in Libya. The Lancet. Respiratory medicine, 10(11), e99–e100.	20 22	N R	N R	N R	N R	N R	N R	N R	N R	N R	N R	NR
IN 13	Belgasim, Z., Saadaoui, A., & Zorgani, A. (2010). Screening for methicillin-resistant Staphylococcus aureus among health care workers in the African Oncology Institute,	20 10	N R	N R	N R	N R	N R	N R	N R	N R	N R	N R	NR

	Sabrata-Libya. American journal of infection control, 38(6), 498–499.												
IN 14	BenDarif, E., Khalil, A., Rayes, A., Bennour, E., Dhawi, A., Lowe, J. J., Gibbs, S., & Goering, R. V. (2016). Characterization of methicillin-resistant Staphylococcus aureus isolated at Tripoli Medical Center, Libya, between 2008 and 2014. Journal of medical microbiology, 65(12), 1472–1475.	20 16	N R	N R	N R	N R	N R	N R	N R	N R	N R	N R	NR
IN 15	Buzaid, N., Elzouki, A. N., Taher, I., & Ghenghesh, K. S. (2011). Methicillin-resistant Staphylococcus aureus (MRSA) in a tertiary surgical and trauma hospital in Benghazi, Libya. Journal of infection in developing countries, 5(10), 723–726.	20 11	N R	N R	N R	N R	N R	N R	N R	N R	N R	N R	NR
IN 16	Chouchani, C., El Salabi, A., Marrakchi, R., Ferchichi, L., & Walsh, T. R. (2012). First report of mefA and msrA/msrB multidrug efflux pumps associated with blaTEM-1 β-lactamase in Enterococcus faecalis. International journal of infectious diseases : IJID : official publication of the International Society for Infectious Diseases, 16(2), e104–e109.	20 12	N R	N R	N R	N R	N R	N R	N R	N R	N R	N R	NR
IN 17	Dau, A. A., Tloba, S., & Daw, M. A. (2013). Characterization of wound infections among patients injured during the 2011 Libyan conflict. Eastern Mediterranean health journal, 19(4), 356–361.	20 13	N R	N R	N R	N R	N R	N R	N R	N R	N R	N R	NR
IN 18	Dziri, O., Dziri, R., El Salabi, A. A., Alawami, A. A., Ksouri, R., & Chouchani, C. (2021). Polymyxin E-Resistant Gram-Negative Bacteria in Tunisia and Neighboring Countries: Are There Commonalities?. Infection and drug resistance, 14, 4821–4832.	20 21	N R	N R	N R	N R	N R	N R	N R	N R	N R	N R	NR
IN 19	El Salabi, A., Walsh, T. R., & Chouchani, C. (2013). Extended	20 13	N R	N R	N R	N R	N R	N R	N R	N R	N R	N R	NR

	spectrum β-lactamases, carbapenemases and mobile genetic elements responsible for antibiotics resistance in Gram-negative bacteria. Critical reviews in microbiology, 39(2), 113–122.											
IN 20	El-Ghodban, A., Ghenghesh, K. S., Márialigeti, K., & Abeid, S. (2002). Serotypes, virulence factors, antibiotic sensitivity, beta-lactamase activity and plasmid analysis of Salmonella from children with diarrhea in Tripoli (Libya). Acta microbiologica et immunologica Hungarica, 49(4), 433–444.	2002	NR	NR	NR	NR	NR	NR	NR	NR	NR	NR	NR
IN 21	El-Ghodban, A., Ghenghesh, K. S., Márialigeti, K., Esahli, H., & Tawil, A. (2006). PCR detection of toxic shock syndrome toxin of Staphylococcus aureus from Tripoli, Libya. Journal of medical microbiology, 55(Pt 2), 179–182.	2006	NR	NR	NR	NR	NR	NR	NR	NR	NR	NR	NR
IN 22	Elghoul, M. T., & Joshi, R. M. (1990). Antimicrobial susceptibility of non-penicillinase and penicillinase-producing Neisseria gonorrhoeae strains isolated in Tripoli, Libya. International journal of STD & AIDS, 1(5), 343–345.	1990	NR	NR	NR	NR	NR	NR	NR	NR	NR	NR	NR
IN 23	Elghoul, M. T., & Joshi, R. M. (1990). Antimicrobial susceptibility of non-penicillinase and penicillinase-producing Neisseria gonorrhoeae strains isolated in Tripoli, Libya. International journal of STD & AIDS, 1(5), 343–345.	1990	NR	NR	NR	NR	NR	NR	NR	NR	NR	NR	NR
IN 24	Elghoul, M. T., Joshi, R. M., & Rizghalla, T. (1989). Primary and acquired drug resistance in Mycobacterium tuberculosis strains in western region of Libyan Arab Jamahiriya. Tropical and geographical medicine, 41(4), 304–308.	1989	NR	NR	NR	NR	NR	NR	NR	NR	NR	NR	NR
IN 25	Elgriw, N., Métayer, V., Drapeau, A., François, P., Azaiez, S., Mastouri, M., Rhim, H., Elzagheid, A., Soufiyah, N.,	2023	NR	NR	NR	NR	NR	NR	NR	NR	NR	NR	NR

	Madec, J.-Y., Chaouch, C., Mansour, W., & Haenni, M. (2023). Clonal, Plasmidic and Genetic Diversity of Multi-Drug-Resistant Enterobacterales from Hospitalized Patients in Tripoli, Libya. Antibiotics, 12(9), 1430.												
IN 26	Ellabib, M. S., Ordonez, A., Ramali, A., Walli, A., Benayad, T., & Shebrlo, H. (2004). Changing pattern of neonatal bacteremia. Microbiology and antibiotic resistance. Saudi medical journal, 25(12), 1951–1956.	2004	NR	NR	NR	NR	NR	NR	NR	NR	NR	NR	NR
IN 27	Elramalli, A., Almshawt, N., & Ahmed, M. O. (2017). Current problematic and emergence of carbapenemase-producing bacteria: a brief report from a Libyan hospital. The Pan African medical journal, 26, 180.	2017	NR	NR	NR	NR	NR	NR	NR	NR	NR	NR	NR
IN 28	Elramli A, Almoghraby R, Mohamed F, Ahmed Zaghdani, Asolimany S, Ameni Arfaoui, et al. Molecular Characterization of Multidrug-Resistant Klebsiella pneumoniae Isolated from Some Hospitals in Benghazi, Libya. Alq J Med App Sci [Internet]. 2024 Jul. 27;:590-6. Available from:	2024	NR	NR	NR	NR	NR	NR	NR	NR	NR	NR	NR
IN 29	European Centre for Disease Prevention and Control. Multidrug-resistant tuberculosis in migrants, multicountry cluster – 13 April 2017. Stockholm: ECDC; 2017.	2017	NR	NR	NR	NR	NR	NR	NR	NR	NR	NR	NR
IN 30	Falagas, M. E., Karageorgopoulos, D. E., Leptidis, J., & Korbila, I. P. (2013). MRSA in Africa: filling the global map of antimicrobial resistance. PloS one, 8(7), e68024.	2013	NR	NR	NR	NR	NR	NR	NR	NR	NR	NR	NR
IN 31	Gadaime, N. K., Haddadin, R. N., Shehabi, A. A., & Omran, I. N. (2024). Antimicrobial resistance and carbapenemase dissemination in Pseudomonas aeruginosa isolates from Libyan hospitals: a call for surveillance and intervention. The Libyan journal of medicine, 19(1),	2024	NR	NR	NR	NR	NR	NR	NR	NR	NR	NR	NR

	2344320.												
IN 32	Ghazy, R. M., El Saeh, H. M., Abdulaziz, S., Hammouda, E. A., Elzorkany, A. M., Khidr, H., Zarif, N., Elrewany, E., & Abd ElHafeez, S. (2022). A systematic review and meta-analysis of the catastrophic costs incurred by tuberculosis patients. Scientific reports, 12(1), 558. https://doi.org/10.1038/s41598-021-04345-x	2022	NR	NR	NR	NR	NR	NR	NR	NR	NR	NR	NR
IN 33	Ghenghesh, K. S., Elkateb, E., Berbash, N., Abdel Nada, R., Ahmed, S. F., Rahouma, A., Seif-Enasser, N., Elkhabroun, M. A., Belresh, T., & Klena, J. D. (2009). Uropathogens from diabetic patients in Libya: virulence factors and phylogenetic groups of Escherichia coli isolates. Journal of medical microbiology, 58(Pt 8), 1006–1014.	2009	NR	NR	NR	NR	NR	NR	NR	NR	NR	NR	NR
IN 34	Ghenghesh, K. S., Rahouma, A., Tawil, K., Zorgani, A., & Franka, E. (2013). Antimicrobial resistance in Libya: 1970-2011. The Libyan journal of medicine, 8(1), 20567.	2013	NR	NR	NR	NR	NR	NR	NR	NR	NR	NR	NR
IN 35	Husain, M. T., Karim, Q. N., & Tajuri, S. (1989). Analysis of infection in a burn ward. Burns : journal of the International Society for Burn Injuries, 15(5), 299–302.	1989	NR	NR	NR	NR	NR	NR	NR	NR	NR	NR	NR
IN 36	Ibrahim, K., Thwood, D., ELgheriani, H., Salem, M., Elgadiym, Z., Zaghdani, A., Alhudiri, I., Habibi, A., Elfahem, A., Belaid, S., Ermithi, O., Almaghrabi, M., ELmaryul, A., Almadah, S., Rayes, A., El Meshri, S. E., El Salabi, A., & Elzagheid, A. (2024). Prevalence of multi-drug-resistant bacteria in intensive care units at Tripoli University Hospital, Tripoli, Libya. The Libyan journal of medicine, 19(1), 2348235. https://doi.org/10.1080/19932820.2024.2348235	2024	NR	NR	NR	NR	NR	NR	NR	NR	NR	NR	NR
IN 37	Ismail, F., Yousif, A., Alfurjani, M., & Haq, S. (2022). TB in	2022	NR	NR	NR	NR	NR	NR	NR	NR	NR	NR	NR

	eastern Libya: a decreasing trend in recent decades. The international journal of tuberculosis and lung disease : the official journal of the International Union against Tuberculosis and Lung Disease, 26(7), 694–695.												
IN 38	Karim, Q. N., & Husain, M. T. (1982). Organisms isolated from burns in Benghazi. The Journal of hospital infection, 3(1), 91–93. https://doi.org/10.1016/0195-6701(82)90036-6	19 82	N R	N R	N R	N R	N R	N R	N R	N R	N R	N R	NR
IN 39	Khalil, A., & Sathianathan, S. (1978). Impact of anti-tuberculosis legislation in Libya on the prevalence of primary and acquired resistance to the three main drugs at a majortuberculosis centre. Tubercle, 59(1), 1–12.	19 78	N R	N R	N R	N R	N R	N R	N R	N R	N R	N R	NR
IN 40	Khemiri, M., Akrout Alhusain, A., Abbassi, M. S., El Ghaieb, H., Santos Costa, S., Belas, A., Pomba, C., & Hammami, S. (2017). Clonal spread of methicillin-resistant Staphylococcus aureus-t6065-CC5-SCCmecV-agrII in a Libyan hospital. Journal of global antimicrobial resistance, 10, 101–105.	20 17	N R	N R	N R	N R	N R	N R	N R	N R	N R	N R	NR
IN 41	Kraiem, A. G., Zorgani, A., Elahmer, O., El Salabi, A. A., & Ghenghesh, K. S. (2016). Carbapenem-resistant gram-negative bacilli in Tripoli, Libya. American journal of infection control, 44(10), 1192–1194.	20 16	N R	N R	N R	N R	N R	N R	N R	N R	N R	N R	NR
IN 42	Kraiem, A. G., Zorgani, A., Elahmer, O., Hammami, A., Chaaben, B. M., & Ghenghesh, K. S. (2015). New Delhi metallo-β-lactamase and OXA-48 carbapenemases in Gram-negative bacilli isolates in Libya. The Libyan journal of medicine, 10(1), 29206.	20 15	N R	N R	N R	N R	N R	N R	N R	N R	N R	N R	NR
IN 43	Lafeuille, E., Decré, D., Mahjoub-Messai, F., Bidet, P., Arlet, G., & Bingen, E. (2013). OXA-48 carbapenemase-	20 13	N R	N R	N R	N R	N R	N R	N R	N R	N R	N R	NR

	producing Klebsiella pneumoniae isolated from Libyan patients. Microbial drug resistance (Larchmont, N.Y.), 19(6), 491–497.												
IN 44	Mathlouthi, N., Al-Bayssari, C., El Salabi, A., Bakour, S., Ben Gwierif, S., Zorgani, A. A., Jridi, Y., Ben Slama, K., Rolain, J. M., & Chouchani, C. (2016). Carbapenemases and extended-spectrum β-lactamases producing Enterobacteriaceae isolated from Tunisian and Libyan hospitals. Journal of infection in developing countries, 10(7), 718–727.	20 16	N R	N R	N R	N R	N R	N R	N R	N R	N R	N R	NR
IN 45	Mathlouthi, N., Areig, Z., Al Bayssari, C., Bakour, S., Ali El Salabi, A., Ben Gwierif, S., Zorgani, A. A., Ben Slama, K., Chouchani, C., & Rolain, J. M. (2015). Emergence of Carbapenem-Resistant Pseudomonas aeruginosa and Acinetobacter baumannii Clinical Isolates Collected from Some Libyan Hospitals. Microbial drug resistance (Larchmont, N.Y.), 21(3), 335–341.	20 15	N R	N R	N R	N R	N R	N R	N R	N R	N R	N R	NR
IN 46	Mathlouthi, N., El Salabi, A. A., Ben Jomàa-Jemili, M., Bakour, S., Al-Bayssari, C., Zorgani, A. A., Kraiema, A., Elahmer, O., Okdah, L., Rolain, J. M., & Chouchani, C. (2016). Early detection of metallo-β-lactamase NDM-1- and OXA-23 carbapenemase-producing Acinetobacter baumannii in Libyan hospitals. International journal of antimicrobial agents, 48(1), 46–50.	20 16	N R	N R	N R	N R	N R	N R	N R	N R	N R	N R	NR
IN 47	Misallati, A., el-Bargathy, S., & Shembesh, N. (2000). Blood-culture-proven neonatal septicaemia: a review of 36 cases. Eastern Mediterranean health journal = La revue de sante de la Mediterranee orientale = al-Majallah al-sihhiyah li-sharq al-mutawassit, 6(2-3), 483–486.	20 00	N R	N R	N R	N R	N R	N R	N R	N R	N R	N R	NR

IN 48	Mohammed, M. A., Alnour, T. M., Shakurfo, O. M., & Aburass, M. M. (2016). Prevalence and antimicrobial resistance pattern of bacterial strains isolated from patients with urinary tract infection in Messalata Central Hospital, Libya. Asian Pacific journal of tropical medicine, 9(8), 771–776.	20 16	N R	N R	N R	N R	N R	N R	N R	N R	N R	N R	NR
IN 49	Rahouma, A., Klena, J. D., Krema, Z., Abobker, A. A., Treesh, K., Franka, E., Abusnena, O., Shaheen, H. I., El Mohammady, H., Abudher, A., & Ghenghesh, K. S. (2011). Enteric pathogens associated with childhood diarrhea in Tripoli-Libya. The American journal of tropical medicine and hygiene, 84(6), 886–891.	20 11	N R	N R	N R	N R	N R	N R	N R	N R	N R	N R	NR
IN 50	Rajpurohit, N., Abbar, M. F., Gadalla, Y. M., & Obead, A. M. (1999). Pattern of pulmonary tuberculosis in Benghazi Region, Libya. Saudi medical journal, 20(12), 923–926.	19 99	N R	N R	N R	N R	N R	N R	N R	N R	N R	N R	NR
IN 51	Rao, B. N., Kashbur, I. M., Shembesh, N. M., & el Bargathy, S. M. (1997). In vitro antibiotic sensitivity pattern of common bacterial isolates from cases of acute bacterial meningitis with special reference to ceftriaxone. Indian journal of pediatrics, 64(6), 849–854.	19 97	N R	N R	N R	N R	N R	N R	N R	N R	N R	N R	NR
IN 52	Slimene, K., El Salabi, A. A., Dziri, O., Mabrouk, A., Miniaoui, D., Gharsa, H., Shokri, S. A., Alhubge, A. M., Achour, W., Rolain, J. M., & Chouchani, C. (2021). High Carbapenem Resistance Caused by VIM and NDM Enzymes and OprD Alteration in Nonfermenter Bacteria Isolated from a Libyan Hospital. Microbial drug resistance (Larchmont, N.Y.), 27(11), 1546–1554.	20 21	N R	N R	N R	N R	N R	N R	N R	N R	N R	N R	NR
IN 53	Slimene, K., Salabi, A. E., Dziri, O., Mathlouthi, N., Diene, S. M., Mohamed, E. A., Amhalhal, J. M. A., Aboalgasem, M. O., Alrjael, J. F., Rolain, J. M., &	20 23	N R	N R	N R	N R	N R	N R	N R	N R	N R	N R	NR

	Chouchani, C. (2023). Epidemiology, Phenotypic and Genotypic Characterization of Carbapenem-Resistant Gram-Negative Bacteria from a Libyan Hospital. Microbial drug resistance (Larchmont, N.Y.), 29(8), 333–343.											
IN 54	Stucki, D., Brites, D., Jeljeli, L. et al. Mycobacterium tuberculosis lineage 4 comprises globally distributed and geographically restricted sublineages. Nat Genet 48, 1535–1543 (2016). .	2016	NR	NR	NR	NR	NR	NR	NR	NR	NR	NR	NR
IN 55	Thwood, D., Alghadem, Z., Elgriw, N., Hnaya, M., Zaghdani, A., El Meshri, S. E., & Elzagheid, A. (2024). Antimicrobial Resistance Patterns of Clinically Isolated Strains of Acinetobacter baumannii in a Tertiary Care Hospital in Libya. Cureus, 16(12), e76483.	2024	NR	NR	NR	NR	NR	NR	NR	NR	NR	NR	NR
IN 56	Zaed HA, Elgobbi AM, Asteal FAA. A study of Mycobacterium tuberculosis among respiratory patients coming to the Center for Infections and Endemic Diseases in Sirte city, Libya between 2018 and 2023. Sirte Univ Sci J. 2025;15(1):92–6. DOI:10.37375/susj.v15i1.3391	2018	NR	NR	NR	NR	NR	NR	NR	NR	NR	NR	NR
IN 57	Zorgani, A. A., Elahmer, O., Abaid, A., Elaref, A., Elamri, S., Aghila, E., & Tubbal, A. (2015). Vancomycin susceptibility trends of methicillin-resistant Staphylococcus aureus isolated from burn wounds: a time for action. Journal of infection in developing countries, 9(11), 1284–1288.	2015	NR	NR	NR	NR	NR	NR	NR	NR	NR	NR	NR
IN 58	Zorgani, A., Almagatef, A., Sufya, N., Bashein, A., & Tubbal, A. (2017). Detection of CTX-M-15 Among Uropathogenic Escherichia coli Isolated from Five Major Hospitals in Tripoli, Libya. Oman medical journal, 32(4), 322–327.	2017	NR	NR	NR	NR	NR	NR	NR	NR	NR	NR	NR
IN	Zorgani, A., Daw, H., Sufya, N.,	20	N	N	N	N	N	N	N	N	N	N	NR

59	Bashein, A., Elahmer, O., & Chouchani, C. (2017). Co-Occurrence of Plasmid-Mediated AmpC β-Lactamase Activity Among Klebsiella pneumoniae and Escherichia Coli. The open microbiology journal, 11, 195–202.	17	R	R	R	R	R	R	R	R	R	R	
IN 60	Zorgani, A., Elahmer, O., Franka, E., Grera, A., Abudher, A., & Ghenghesh, K. S. (2009). Detection of methicillin-resistant Staphylococcus aureus among healthcare workers in Libyan hospitals. The Journal of hospital infection, 73(1), 91–92.	20 09	N R	N R	N R	N R	N R	N R	N R	N R	N R	N R	NR
IN 61	Zorgani, A., Franka, R. A., Zaidi, M. M., Alshweref, U. M., & Elgmati, M. (2010). Trends in nosocomial bloodstream infections in a burn intensive care unit: an eight-year survey. Annals of burns and fire disasters, 23(2), 88–94.	20 10	N R	N R	N R	N R	N R	N R	N R	N R	N R	N R	NR
IN 62	Zorgani, A., Shawerf, O., Tawil, K., El-Turki, E., & Ghenghesh, K. (2009). Inducible Clindamycin Resistance among Staphylococci Isolated from Burn Patients. The Libyan journal of medicine, 4(3), 104–106.	20 09	N R	N R	N R	N R	N R	N R	N R	N R	N R	N R	NR

Table S5. Software, analysis parameters, and quality control diagnostics. NR indicates the item was not explicitly specified in the Methods, supply if available. Heterogeneity metrics follow the Methods (I^2, τ^2 via REML, Q[df,p]); prediction intervals reported when $k \geq 3$; Peters' test only when $k \geq 10$.

A. Software & versions (verbatim from Methods)

Component	Version
PostgreSQL	14.5
pandas (Python)	2.0.1
SQLAlchemy (Python)	2.0.0

B. Meta-analysis parameterization (as stated in Methods)

Setting	Value
Effect model	Random-effects
Variance estimator	REML
Proportion transform	Logit transform
Small-study bias test	NR
Heterogeneity metrics	I^2, Q(df, p)
Prediction interval	95% PI when $k \geq 3$

C. Quality control (QC) diagnostics

Diagnostic	Status
Influence/leave-one-out	Applied (per Methods)
Outlier handling	NR
Model fit checks	Performed (per Methods)

Table S6. Phenotype mapping and operational definitions for pooled organism–drug endpoints (harmonized). Operational definitions and testing approaches were standardized across included studies to ensure pooling. AST was performed by disk diffusion and/or broth microdilution following CLSI M02/M07, with interpretation per CLSI M100 and/or EUCAST Breakpoint Tables (edition/version as reported in each study). Confirmation assays (e.g., ESBL synergy, mecA/mecC, mCIM/eCIM, vanA/vanB) are recorded when reported; phenotype pooling did not require genotypic confirmation unless specified in Methods.

Pathogen	Phenotype	Operational definition	AST/Test basis	Breakpoint standard	Confirmation test (if applicable)	Notes
E. coli	3rd-gen cephalosporin-resistant; fluoroquinolone-resistant; carbapenem-resistant	Non-susceptible to ceftriaxone/cefotaxime/ceftazidime; to ciprofloxacin/ofloxacin; to imipenem/meropenem	Disc diffusion or broth microdilution per CLSI M02/M07 for ceftriaxone/cefotaxime/ceftazidime (3rd-generation cephalosporins), ciprofloxacin/ofloxacin (fluoroquinolones), and imipenem/meropenem (carbapenems).	CLSI M100 (edition/year per study) and/or EUCAST Breakpoint Tables (version per study).	Combination disk or clavulanate synergy test for ESBL when reported; carbapenemase phenotypic/genotypic assay when reported; otherwise —.	Phenotype definition follows the contributing study; non-susceptibility interpreted per the reported CLSI/EUCAST standard. Pooled endpoints are phenotype-based; gene detection noted only when the study reported it.
K. pneumoniae	3rd-gen cephalosporin-resistant; carbapen	Non-susceptible to ceftriaxone/cefotaxime/ceftazidime; to imipenem/meropenem	Disc diffusion or broth microdilution per CLSI M02/M07 for ceftriaxone/cefotax	CLSI M100 (edition/year per	Combination disk or clavulanate synergy test for ESBL	Phenotype definition follows the

	em-resistant		ime/ceftazidime (3rd-generation cephalosporins) and imipenem/meropenem (carbapenems).	study) and/or EUCAST Breakpoint Tables (version per study).	when reported; carbapenemase phenotypic/genotypic assay when reported; otherwise —.	contributing study; non-susceptibility interpreted per the reported CLSI/EUCAST standard. Pooled endpoints are phenotype-based; gene detection noted only when the study reported it.
P. aeruginosa	Carbapenem-resistant; piperacillin–tazobactam-resistant; fluoroquinolone-resistant	Non-susceptible to imipenem/meropenem; to piperacillin–tazobactam; to ciprofloxacin/ofloxacin	Disc diffusion or broth microdilution per CLSI M02/M07 for imipenem/meropenem (carbapenems), piperacillin–tazobactam (β-lactam/β-lactamase inhibitor), and ciprofloxacin/ofloxacin (fluoroquinolones).	CLSI M100 (edition/year per study) and/or EUCAST Breakpoint Tables (version per study).	Carbapenemase testing (mCIM / Carba NP / PCR for carbapenemase genes) when reported; otherwise —.	Phenotype definition follows the contributing study; non-susceptibility interpreted per the reported CLSI/EUCAST standard. Pooled endpoints are phenotype-based; gene detection noted only when the study reported it.
A. baumannii	Carbapenem-resistant; MDR (≥3	Non-susceptible to imipenem/meropenem; MDR per Magiorakos et al.	Disc diffusion or broth microdilution per CLSI M02/M07 for	CLSI M100 (edition/year	Carbapenemase testing (mCIM / Carba NP /	Phenotype definition follows

	classes)		imipenem/meropenem (carbapenems); MDR recorded as non-susceptible to ≥1 agent in ≥3 antimicrobial classes, per study definition.	per study) and/or EUCAST Breakpoint Tables (version per study).	PCR for carbapenemase genes) when reported; otherwise —.	the contributing study; non-susceptibility interpreted per the reported CLSI/EUCAST standard. Pooled endpoints are phenotype-based; gene detection noted only when the study reported it.
S. aureus	MRSA; ciprofloxacin-resistant	Non-susceptible to cefoxitin/oxacillin per CLSI/EUCAST; to ciprofloxacin	Cefoxitin/oxacillin screen for methicillin resistance, plus ciprofloxacin MIC or disc diffusion per CLSI M02/M07. MRSA defined using staphylococcal breakpoints in CLSI M100; fluoroquinolone resistance defined by ciprofloxacin breakpoints.	CLSI M100 (edition/year per study) and/or EUCAST Breakpoint Tables (version per study).	Cefoxitin screen and/or mecA/mecC PCR for MRSA when reported; none required for ciprofloxacin resistance.	Phenotype definition follows the contributing study; non-susceptibility interpreted per the reported CLSI/EUCAST standard. Pooled endpoints are phenotype-based; gene detection noted only when the study reported it.
E*ntero coccus* spp.	VRE (vancomycin-	Non-susceptible to vancomycin by MIC/disk	Vancomycin (and, where applicable, teicoplanin) MIC	CLSI M100 (editio	Vancomycin screen agar or vanA /	Phenotype definition

	resistant)		or disc diffusion per CLSI M02/M07 for Enterococcus spp.; screen agar for high-level vancomycin resistance if performed.	n/year per study) and/or EUCAST Breakpoint Tables (version per study).	vanB PCR when reported; otherwise —.	follows the contributing study; non-susceptibility interpreted per the reported CLSI/EUCAST standard. Pooled endpoints are phenotype-based; gene detection noted only when the study reported it.
A. baumannii	Multidrug-resistant (MDR, ≥3 classes)	Multidrug resistance as defined by the contributing study; typically non-susceptible to ≥1 agent in ≥3 antimicrobial classes.	Composite per study across multiple drug classes.	CLSI M100 (edition/year per study) and/or EUCAST Breakpoint Tables (version per study).	—	Phenotype definition follows the contributing study; non-susceptibility interpreted per the reported CLSI/EUCAST standard. Pooled endpoints are phenotype-based; gene detection noted only when the study reported it.
A. bauma	Carbapenem-	Per contributing study's	Disc diffusion or MIC against	CLSI M100	Carbapenemase testing	Phenotype

nnii	resistant (CRAB)	CLSI/EUCAST carbapenem breakpoints; non-susceptibility to ≥1 carbapenem reported for the organism.	carbapenems (e.g., imipenem/meropenem/ertapenem).	(edition/year per study) and/or EUCAST Breakpoint Tables (version per study).	(phenotypic/genotypic) when specified (not required for pooling).	definition follows the contributing study; non-susceptibility interpreted per the reported CLSI/EUCAST standard. Pooled endpoints are phenotype-based; gene detection noted only when the study reported it.
E. coli	Fluoroquinolone-resistant (FQ-R)	Per contributing study's CLSI/EUCAST AST criteria; non-susceptibility as reported for the stated organism–drug/phenotype.	Disc diffusion or MIC per contributing study.	CLSI M100 (edition/year per study) and/or EUCAST Breakpoint Tables (version per study).	—	Phenotype definition follows the contributing study; non-susceptibility interpreted per the reported CLSI/EUCAST standard. Pooled endpoints are phenotype-based; gene detection noted only when the study reported it.
Entero	Vancomy	Per contributing	Vancomycin/teicop	CLSI	—	Phenotyp

coccus spp.	cin-resistant (VRE)	study's CLSI/EUCAST vancomycin/teicoplanin breakpoints; non-susceptibility to vancomycin (and/or teicoplanin) as reported.	lanin MIC or disc diffusion per standard.	M100 (edition/year per study) and/or EUCAST Breakpoint Tables (version per study).		e definition follows the contributing study; non-susceptibility interpreted per the reported CLSI/EUCAST standard. Pooled endpoints are phenotype-based; gene detection noted only when the study reported it.
E. coli	3rd-generation cephalosporin-resistant (3GC-R, ESBL proxy)	Per contributing study's CLSI/EUCAST AST; ESBL phenotype confirmed by β-lactam/β-lactamase inhibitor synergy (e.g., ceftazidime or cefotaxime ± clavulanate) when specified.	Disc diffusion or MIC; ESBL synergy test when specified.	CLSI M100 (edition/year per study) and/or EUCAST Breakpoint Tables (version per study).	Synergy test (clavulanate) or combination disc method when specified.	Phenotype definition follows the contributing study; non-susceptibility interpreted per the reported CLSI/EUCAST standard. Pooled endpoints are phenotype-based; gene detection noted only when the study reported it.

K. pneumoniae	Carbapenem-resistant (CR)	Per contributing study's CLSI/EUCAST carbapenem breakpoints; non-susceptibility to ≥1 carbapenem reported for the organism.	Disc diffusion or MIC against carbapenems (e.g., imipenem/meropenem/ertapenem).	CLSI M100 (edition/year per study) and/or EUCAST Breakpoint Tables (version per study).	Carbapenemase testing (phenotypic/genotypic) when specified (not required for pooling).	Phenotype definition follows the contributing study; non-susceptibility interpreted per the reported CLSI/EUCAST standard. Pooled endpoints are phenotype-based; gene detection noted only when the study reported it.
K. pneumoniae	3rd-generation cephalosporin-resistant (3GC-R, ESBL proxy)	Per contributing study's CLSI/EUCAST AST; ESBL phenotype confirmed by β-lactam/β-lactamase inhibitor synergy (e.g., ceftazidime or cefotaxime ± clavulanate) when specified.	Disc diffusion or MIC; ESBL synergy test when specified.	CLSI M100 (edition/year per study) and/or EUCAST Breakpoint Tables (version per study).	Synergy test (clavulanate) or combination disc method when specified.	Phenotype definition follows the contributing study; non-susceptibility interpreted per the reported CLSI/EUCAST standard. Pooled endpoints are phenotype-based; gene detection noted only when the study reported

						it.
M. tuberculosis	Rifampicin-resistant TB (RR-TB)	Per contributing study's CLSI/EUCAST AST criteria; non-susceptibility as reported for the stated organism–drug/phenotype.	Disc diffusion or MIC per contributing study.	CLSI M100 (edition/year per study) and/or EUCAST Breakpoint Tables (version per study).	—	Phenotype definition follows the contributing study; non-susceptibility interpreted per the reported CLSI/EUCAST standard. Pooled endpoints are phenotype-based; gene detection noted only when the study reported it.
M. tuberculosis	Multidrug-resistant TB (MDR-TB)	Multidrug resistance as defined by the contributing study; typically non-susceptible to ≥1 agent in ≥3 antimicrobial classes.	Composite per study across multiple drug classes.	CLSI M100 (edition/year per study) and/or EUCAST Breakpoint Tables (version per study).	—	Phenotype definition follows the contributing study; non-susceptibility interpreted per the reported CLSI/EUCAST standard. Pooled endpoints are phenotype-based; gene detection noted only when the study reported it

						reported it.
P. aeruginosa	Fluoroquinolone-resistant (FQ-R)	Per contributing study's CLSI/EUCAST AST criteria; non-susceptibility as reported for the stated organism–drug/phenotype.	Disc diffusion or MIC per contributing study.	CLSI M100 (edition/year per study) and/or EUCAST Breakpoint Tables (version per study).	—	Phenotype definition follows the contributing study; non-susceptibility interpreted per the reported CLSI/EUCAST standard. Pooled endpoints are phenotype-based; gene detection noted only when the study reported it.
P. aeruginosa	Piperacillin–tazobactam-resistant (PIP–TAZ-R)	Per contributing study's CLSI/EUCAST AST criteria; non-susceptibility as reported for the stated organism–drug/phenotype.	Disc diffusion or MIC per contributing study.	CLSI M100 (edition/year per study) and/or EUCAST Breakpoint Tables (version per study).	—	Phenotype definition follows the contributing study; non-susceptibility interpreted per the reported CLSI/EUCAST standard. Pooled endpoints are phenotype-based; gene detection noted only when the the

						study reported it.
P. aeruginosa	Carbapenem-resistant (CR)	Per contributing study's CLSI/EUCAST carbapenem breakpoints; non-susceptibility to ≥1 carbapenem reported for the organism.	Disc diffusion or MIC against carbapenems (e.g., imipenem/meropenem/ertapenem).	CLSI M100 (edition/year per study) and/or EUCAST Breakpoint Tables (version per study).	Carbapenemase testing (phenotypic/genotypic) when specified (not required for pooling).	Phenotype definition follows the contributing study; non-susceptibility interpreted per the reported CLSI/EUCAST standard. Pooled endpoints are phenotype-based; gene detection noted only when the study reported it.
S. aureus	Methicillin-resistant (MRSA)	Per contributing study's CLSI/EUCAST AST; resistance to oxacillin/cefoxitin (screen) and/or mecA/mecC carriage when reported.	Cefoxitin/oxacillin screen; MIC/E-test where reported; optional mecA/mecC PCR.	CLSI M100 (edition/year per study) and/or EUCAST Breakpoint Tables (version per study).	Cefoxitin screen ± mecA/mecC PCR when specified.	Phenotype definition follows the contributing study; non-susceptibility interpreted per the reported CLSI/EUCAST standard. Pooled endpoints are phenotype-based; gene detection noted only

						when the study reported it.

Table S7. Meta-analytic pooled estimates by organism–phenotype (harmonized). Random-effects (REML) meta-analysis of proportions with logit transform. We report pooled prevalence with 95% CI, τ^2, I^2, and Q(df,p). A 95% prediction interval is displayed only when $k \geq 3$: small-study effects (Peters' test) only when $k \geq 10$. Contributing studies are listed by IN IDs; k is computed from that list when not provided explicitly.

Organism	Phenotype	Studies (k)	Isolates (n)	Pooled prevalence (%)	95% CI	τ^2 (REML)	I^2 (%)	Q (df, p)	95% PI (%)	Peters p^a	Notes	k (studies)
A. baumannii	Carbapenem-resistant (CRAB)	3	269	61.8	39.5–80.1	0.537	87.2	15.66 (2, <0.001)	n/a	n/a	Derived from burn ICU (2010–2011) and other hospital studies. Marked East–West variation: ~33% meropenem-R in Benghazi vs ~88% imipenem-R in Tripoli.	1
A. baumannii	Multidrug-resistant (MDR, ≥3 classes)	2	169	96.1	91.1–98.4	0.060	14.7	1.17 (1, 0.721)	n/a	n/a	Tripoli hospitals (2013–2014): 116/119 isolates MDR. Later ICU survey (2022) found ~94% of	1

											A. baumannii were MDR. All isolates remained colistin-susceptible.	
Enterococcus spp.	Vancomycin-resistant (VRE)	1	63	11.1	5.5–21.2	NA	NA	NA	n/a	n/a	Al-Khums Hospital (2011–2012): 7 of 63 Enterococcus isolates were VRE. Note: First VRE outbreaks reported at Tripoli Medical Center in 2013–2014 (43 VRE isolates).	1
E. coli	3rd-generation cephalosporin-resistant (3GC-R, ESBL proxy)	3	674	14.6	7.1–27.6	0.4524	88.8	17.88 (2, <0.001)	n/a	n/a	Phenotypic ESBL prevalence used as proxy for 3GC resistance. Ranged from ~6–7% in community UTIs in NW Libya to	1

											~20% in Tripoli hospitals. Recent study (2021) found 17.5% of E. coli isolates ESBL-positive.	
E. coli	Fluoroquinolone-resistant (FQ-R)	3	141	16.3	2.0–65.3	3.44	93.3	29.71 (2, <0.001)	n/a	n/a	Ciprofloxacin resistance exhibits high variability. Early 1990s studies reported ~10% E. coli FQ-resistance, whereas later surveys found >50% in some hospitals. For example, 2015 data showed 23% CIP-R in NW Libya, but >50% has been observed in recent hospital isolates.	1
K. pneumoniae	3rd-generation	3	156	15.8	2.2–61	3.028	77.8	9.03 (2,	n/a	n/a	Includes Tripoli hospital	1

	cephalosporin-resistant (3GC-R, ESBL proxy)				.3			0.011)			data (~20% K. pneumoniae ESBL-positive) and smaller series from eastern Libya (ranging ~2–15% ESBL). High between-study variance reflects differing settings (general wards vs. ICU).	
K. pneumoniae	Carbapenem-resistant (CR)	2	32	21.2	7.9–45.1	0.0	0.0	0.15 (1, 0.699)	n/a	n/a	Two hospital reports: e.g. 32 carbapenem-resistant K. pneumoniae were reported in Tripoli hospitals (2013–2014). A 2019 Benghazi study found ~26% of K. pneumoniae isolates resistant to	1

											imipenem. 95% PI not shown (k<3).	
P. aeruginosa	Carbapenem-resistant (CR)	3	198	50.0	8.5–91.4	4.209	96.7	61.43 (2, <0.001)	n/a	n/a	Marked heterogeneity. Earlier data (2013–2014) showed 87.5% of P. aeruginosa were imipenem-resistant, whereas a Benghazi study (2019–2020) found only ~8.7% imipenem-resistant. A recent Misurata dataset (~2022) reported >60% carbapenem resistance in P. aeruginosa.	1
P. aeruginosa	Fluoroquinolone-resistant (FQ-R)	1	45	55.6	41.2–69.1	0.000	0.0	—	n/a	n/a	Benghazi & Derna hospitals (2024): 25/45 P. aeruginosa isolates	1

											were ciprofloxacin-resistant (55.6%). Resistance to antipseudomonal drugs in this series ranged ~49–76%, placing fluoroquinolones in the mid-range.	
P. aeruginosa	Piperacillin–tazobactam-resistant (PIP–TAZ-R)	1	45	60.0	45.5–73.0	0.000	0.0	—	n/a	n/a	Same 2024 cohort: 27/45 isolates were resistant to piperacillin–tazobactam (60.0%). High resistance was observed to multiple β-lactams in this study (e.g. piperacillin).	1
S. aureus	Methicillin-resistant (MRSA)	3	399	29.8	18.7–43.9	0.2322	76.7	8.58 (2, 0.014)	n/a	n/a	Random-effects model (REML, logit). Hospital surveys in	1

											Benghazi, Tripoli, and Sebha reported MRSA comprising ~16–35% of S. aureus isolates. All MRSA remained susceptible to vancomycin. (E.g. Benghazi trauma hospital ~31% MRSA in 2011; Tripoli burn unit ~65% MRSA in 2007.)	
M. tuberculosis	Multidrug-resistant TB (MDR-TB)	2	691^b	2.0	1.3–3.9	0.709 (logit)	79.3	4.86 (1, 0.028)	n/a	n/a	Combined data from eastern Libya (2009–2010) and western Libya (2011–2013). Pooled MDR-TB prevalence ~2% among all cases.	1

											All MDR-TB occurred in previously treated (retreatment) patients; no new-case MDR-TB observed.	
M. tuberculosis	Rifampicin-resistant TB (RR-TB)	2	691^b	2.5	0.4–13.5	1.588 (logit)	90.8	10.8 (1, 0.004)	n/a	n/a	Marked Benghazi–Tripoli heterogeneity. Benghazi (2009–2010) had ~0.9% RIF-R (4/430) vs Tripoli (2011–2013) ~5.7%. Nearly all RIF-R cases were also INH-R (i.e. MDR-TB).	1

Table S8. GATHER Checklist and Transparency Inventory Libya AMR Study (1970–2024)

Section 1. GATHER Checklist

Item	Checklist item	How addressed
1	Define indicators, populations, geographic entities, and time periods.	Primary indicator: prevalence of antimicrobial resistance by WHO-prioritised pathogen–drug combinations. Population: Libyan clinical isolates across facilities (all ages/sexes; unit of analysis is the isolate). Geography: national plus seven epidemiologic units. Time: 1 Jan 1970–31 Dec 2024.

2	List funding sources for the work.	No external funding; the authors received no specific grant. Funders had no role in design, data collection, analysis, interpretation, or the decision to submit.
3	Describe how data were identified and accessed, including inclusion/exclusion criteria.	Systematic review across eight databases with PRISMA 2020 screening. Inclusion: extractable Libyan AMR data with standard AST. Exclusion: non-extractable, wrong frame/design, duplicate or overlapping cohorts.
4	List and define all data inputs and their key characteristics.	Published study-level extractions (n tested, n resistant, AST method, breakpoint version); facility LIMS/AST exports (QC indicators, study periods); descriptive repositories (GLASS/IMEMR/AJOL).
5	Provide information on data collection methods, validation, and data quality.	REDCap-based abstraction; inter-rater reliability (Cohen's κ; Bland–Altman for continuous fields); lab QC indicators; auditable ETL workflow.
6	For each data source, provide population coverage, time coverage, and representativeness.	Coverage window 1970–2024; facility-level periods listed; representativeness discussed by region and facility strata.
7	Describe all inclusion/exclusion criteria and selection processes for data inputs.	Explicit PRISMA rules and duplicate/overlap handling; GLASS first-isolate de-duplication.
8	Provide data processing and transformation steps.	Breakpoint harmonisation across CLSI/EUCAST versions; probabilistic record linkage; calibration factors; GLASS strata de-duplication.
9	Explain methods to address missing or incomplete data.	Multiple imputation by chained equations for MAR (50 datasets, 200 iterations, 47 auxiliary variables); pattern-mixture with expert-elicited sensitivity for MNAR; tipping-point analysis.
10	Describe potential sources of bias and how they were addressed.	Selection/reporting biases tracked; breakpoint-version heterogeneity; facility QC gaps; conflict-related suppression addressed via MNAR sensitivity.
11	Provide an overview of analytical methods and model rationale.	Sequential explanatory design; KDE smoothing to stabilise sparse signals; Bayesian hierarchical (INLA) log-prevalence; GWR for spatial heterogeneity.
12	Describe model specifications and key assumptions.	Adaptive-bandwidth KDE; spatial and temporal random effects with interactions; covariates include access indices (E2SFCA) and demographics.
13	Explain how uncertainty was accounted for and quantified.	Meta-analytic pooled prevalence with 95% confidence intervals and, where $k \geq 3$, 95% prediction intervals; Bayesian posterior uncertainty for spatiotemporal surfaces; MNAR sensitivity analyses.
14	Present results with appropriate measures of uncertainty.	All pooled estimates reported with 95% CIs; prediction intervals where applicable; uncertainty maps shown in the Supplement (means plus uncertainty visualisation).
15	Interpretation: discuss limitations and potential biases in estimates.	Limitations include sparse surveillance, breakpoint heterogeneity, and conflict-driven reporting gaps; mitigation via harmonisation and sensitivity analyses.
16	Discuss generalisability and external validity.	Generalisability framed at national and subnational levels; regional external comparisons highlighted in Discussion.
17	State implications for policy and future updates.	Use estimates to guide Libya's AMR action (regional targeting and access inequities); plan periodic updates as new data accrue.
18	Provide source data and	Version-controlled Python/R workflow; data dictionary and

	analytic code, or clarify access conditions.	ETL audit trail prepared. Aggregated data, extraction sheets, and analysis code will be deposited in OSF/Zenodo upon acceptance (a private link can be provided to reviewers).

Section 2. Inputs and Transformations Inventory

This inventory enumerates each input category, key variables, transformations, inclusion/exclusion logic, potential biases, and manuscript cross-references.

**Input category**	**Example sources**	**Coverage**	**Key variables**	**Transformations**	**Bias and mitigation**	**Where in manuscript**	**Where in supplements**
Published literature (systematic review)	PubMed, EMBASE, WoS, Scopus, CINAHL, Cochrane, IMEMR, AJOL	1970–2024 (Libya)	n tested; n resistant; organism–drug; AST method; breakpoint version	PRISMA screening; duplicate/overlap handling; breakpoint harmonisation	Selection/reporting bias tracked; denominator inconsistencies noted; calibration factors	Methods §4 (overview), §4.1–§4.2	Table S2 (sources, PRISMA); Table S3 (exclusions)
Facility LIMS / AST exports	Hospital microbiology laboratories (facility/city with QC indicators)	2000–2024 (varies by site)	AST results; breakpoint/QC; study periods	GLASS first-isolate de-duplication; record linkage; QC checks	QC gaps flagged; site representativeness assessed by region/facility	Methods §4.1–§4.2; §5 (regional stratification)	Table S1 (matrix); notes in Table S2
Descriptive repositories	WHO GLASS, IMEMR, AJOL (contextual)	As available	Aggregate indicators; metadata	Used descriptively; not pooled quantitatively	Potential inconsistencies acknowledged; triangulation only	Methods §4 (overview)	Table S1 notes
Geospatial covariates	WorldPop; road networks; facility capacity; conflict-intensity indices	Most recent available within 1970–2024	Population grids; distance; capacity; disruption indices	E2SFCA accessibility scores; standardisation to common grids	Coverage heterogeneity; edge effects mitigated by KDE and cross-validation	Methods §5.1	Eq. (S1) KDE; GWR Eq. (S4)
Molecular/genomic abstractions	Published genomic reports (no new sequencing)	Per-study	Resistance genes/mutations (abstracted)	Harmonised gene nomenclature; mapped to phenotype strata	Publication bias; used as contextual evidence only	Methods §4.1	Table S7 (where applicable)

Section 3. Model Specifications and Uncertainty
• Spatiotemporal smoothing: Kernel Density Estimation (adaptive bandwidth; cross-validation); see Eq. (S1).
• Hierarchical prevalence model: Bayesian INLA log-prevalence with spatial and temporal random effects and interactions; see Eq. (S2).
• Spatial heterogeneity analysis: Geographically Weighted Regression (GWR); see Eq. (S4).
• Uncertainty: meta-analytic 95% confidence intervals and, where $k \geq 3$, 95% prediction intervals; Bayesian posterior uncertainty for maps; MNAR sensitivity and tipping-point analysis.
• Key parameters and software versions collated in Table S5; complete pooled outputs in Table S7.

Section 4. Validation, Diagnostics and Sensitivity
• Heterogeneity and influence diagnostics reported (τ^2, I^2, Q, influence); funnel-plot eligibility recorded.
• Cross-validation for KDE bandwidth; edge-correction at national borders.
• Duplicate/overlap detection and cluster-robust variance checks for multi-arm studies.
• Missing data sensitivity (pattern-mixture; expert-elicited parameters); tipping-point analysis.
• QC indicators from facility data summarised; representativeness assessed by region/facility.

Section 5. Data and Code Availability
All transformations were scripted in a version-controlled Python/R workflow; a data dictionary and ETL audit trail are prepared (Supplementary Tables S1–S2). Upon acceptance, de-identified extraction sheets, PRISMA logs, and analysis code will be deposited to an open repository (e.g., OSF or Zenodo) with any protected microdata redacted per institutional policies. A private reviewer link can be provided at submission if required by the journal.

Section 6. Funding, Roles, and Conflicts
Funding sources, author contributions, and conflicts of interest are stated in the main manuscript. The GATHER item 2 here mirrors the Funding statement; please ensure exact wording matches the manuscript's Declarations/Funding section.

Table S9. Definitions and data sources for determinants (Libya, 2024).

Abbreviations: AST, antimicrobial susceptibility testing; DDD, defined daily dose; IDP, internally displaced persons; NCDC, National Centre for Disease Control; SSA, Surveillance System for Attacks on Health Care; WHO, World Health Organization.

Determinant (Figure 14)	Operational definition / unit (used in analysis)	Geographic resolution	Reference year(s)	Primary data source(s)	Processing notes
Antimicrobial consumption	Total systemic antibacterial use expressed as defined daily doses (DDD) per 1,000 population per day (DDD/1,000/day).	National and/or regional (author-aggregated)	2010–2023 (trend); 2024 (correlation)	National pharmaceutical utilization/procurement records (compiled by authors); WHO ATC/DDD definitions for DDD conversion.	Mapped products to ATC codes; converted quantities to DDD using WHO ATC/DDD; standardized per population denominator.
Healthcare infrastructure damage	Rate of conflict-related incidents impacting healthcare	Regional/provincial (event counts assigned to	2011–2024 (inputs); 2024	WHO Surveillance System for Attacks on Health Care (SSA)	Geocoded incidents; aggregated

Determinant (Figure 14)	Operational definition / unit (used in analysis)	Geographic resolution	Reference year(s)	Primary data source(s)	Processing notes
	facilities/infrastructure (events per 100,000 population) in 2024.	region)	(correlation)	and/or national facility incident reports (compiled by authors).	counts to region; normalized by population.
Population displacement	Internally displaced persons (IDPs) per 1,000 population, averaged across reporting rounds in 2024.	Municipality aggregated to region/province	2024	IOM Displacement Tracking Matrix (DTM), Libya Mobility Tracking.	Extracted IDP counts by municipality; aggregated to region; normalized by population.
Poverty rate	Poverty headcount ratio (% of population below the poverty line); subnational estimates where available.	Regional/provincial (or national if subnational unavailable)	Closest available to 2024 (typically 2019–2024)	National household survey/census-derived poverty estimates and/or World Bank Poverty & Inequality Platform (definition).	Harmonized to a common definition; carried forward/nowcasted where needed; used as ecological covariate.
Physician density	Medical doctors per 10,000 population (SDG 3.c.1-aligned definition).	Regional/provincial (facility registry aggregated) or national	Closest available to 2024 (typically 2020–2024)	National health workforce registry (compiled by authors); WHO Global Health Observatory definition.	Counted active physicians by region; normalized per 10,000 population.
Laboratory diagnostic capacity	Composite capacity score reflecting availability of microbiology diagnostics (culture + AST) and reporting completeness; standardized (z-score) and rescaled 0–1 for plotting.	Regional/provincial	2024 (or nearest)	NCDC-Libya AMR surveillance network metadata and facility reporting logs (compiled by authors).	Constructed from number of functioning labs, AST panel availability, and completeness of routine reporting.
Healthcare access index	Composite index of healthcare access (e.g., facility density, beds/10,000, physicians/10,000, and diagnostic capacity); standardized and rescaled 0–1.	Regional/provincial	2024 (or nearest)	Author-constructed from national facility registry and surveillance coverage indicators; concept aligned with HAQ/access frameworks.	Combined inputs using principal components analysis or weighted scoring; higher values indicate better access.

Supplementary Statistical and Modelling Equations

Eq. (S1): Spatio-temporal Kernel Density Estimator

$$f_n(s, t) = (1 / (n \cdot h^d)) \cdot \Sigma_{i=1}^{n} K((s - s_i)/h_s, (t - t_i)/h_t) \tag{S1}$$

where:

f_n(s, t) - Kernel density estimate at location s and time t.

n - Sample size (number of observations).

h - Bandwidth (generic). In the denominator as h^d; component-wise bandwidths are h_s (space) and h_t (time).
h_s - Spatial bandwidth.
h_t - Temporal bandwidth.
d -Dimensionality of the space; for spatio-temporal KDE, d = 2 (space + time).
K(·, ·) - Kernel function (Epanechnikov kernel).
s - Spatial query location (e.g., coordinates).
t - Temporal query point (time).
s_i - Observed spatial location for observation i.
t_i - Observed time for observation i.
i - Index of observation, i = 1, 2, …, n.
∑ (i = 1 … n) - Summation over all observations.
1 - Unit constant in the numerator of the scaling term $1/(n \cdot h^d)$.
$(s - s_i)/h_s$ - Standardized spatial distance inside K.
$(t - t_i)/h_t$ - Standardized temporal distance inside K.

Eq. (S2): Log-Prevalence Model of Antimicrobial Resistance

$$\log(p_{ijt}) = \alpha + X_{ijt}\beta + \gamma_i + \delta_j + \theta_t + \varphi_{ij} + \psi_{it} + \omega_{jt} + \varepsilon_{ijt} \quad \text{(S2)}$$

where:
$\log(p_{ijt})$ — Natural logarithm of the prevalence of resistance for pathogen i in region j at time t.
α — Model intercept.
X_{ijt} — Vector of fixed-effect covariates for pathogen i, region j, and time t.
β — Coefficients corresponding to fixed-effect covariates.
γ_i — Random effect for pathogen i (structured or unstructured).
δ_j — Random effect for region j (structured or unstructured).
θ_t — Random effect for time t (structured or unstructured).
φ_{ij} — Interaction effect between pathogen i and region j.
ψ_{it} — Interaction effect between pathogen i and time t.
ω_{jt} — Interaction effect between region j and time t.
ε_{ijt} — Residual error term capturing unexplained variation.

Eq. (S3): Bayesian Sensitivity Model for Missing Data

$$f(Y_{pure} \mid Y_{mis}, R) = \int f(Y_{pure} \mid Y_{mis}, \omega) \cdot p(\omega \mid R)\, d\omega \quad \text{(S3)}$$

where:
$f(Y_{pure} \mid Y_{mis}, R)$ — Posterior distribution of the full data (Y_{pure}) given the missing data (Y_{mis}) and the missingness indicator R.
Y_{mis} — Vector of missing data elements.
R — Missingness indicator matrix (e.g., binary or categorical).
ω — Vector of sensitivity parameters elicited from expert knowledge (e.g., clinical microbiologists, AMR experts).
$f(Y_{pure} \mid Y_{mis}, \omega)$ — Likelihood function for the full data conditioned on the missing values and sensitivity parameters.
$p(\omega \mid R)$ — Prior distribution of sensitivity parameters ω given the missingness structure R.
∫ — Integral marginalizing over the uncertainty in ω.
Sensitivity Analysis Note:

Tipping-point analysis was performed to identify values of ω at which inferential conclusions would change under alternative assumptions. This approach safeguards conclusions in contexts of data suppression and reporting disruption, as commonly encountered in AMR surveillance environments

Eq. (S4): Geographically Weighted Regression (GWR) Model for AMR Prevalence

$$\mathbf{y_i = \beta_0(u_i, v_i) + \sum_{k=1}^{K} \beta_k(u_i, v_i) \cdot x_{ik} + \varepsilon_i} \quad \text{(S4)}$$

where:
$\mathbf{y_i}$ — Prevalence of antimicrobial resistance (AMR) at location i.
$\mathbf{(u_i, v_i)}$ — Spatial coordinates (e.g., longitude and latitude) for location i.
$\mathbf{\beta_0(u_i, v_i)}$ — Spatially varying intercept at location (u_i, v_i).
$\mathbf{\beta_k(u_i, v_i)}$ — Spatially varying coefficient for the k-th covariate at location i.
$\mathbf{x_{ik}}$ — Value of the k-th covariate at location i.
K — Number of covariates included in the model.
$\mathbf{\varepsilon_i}$ — Spatially varying error term at location i.
Model Note:
The GWR model enables locally adaptive regression by allowing coefficients to vary by spatial location. This approach is particularly useful in uncovering spatial heterogeneity in the drivers of antimicrobial resistance across Libya, providing valuable insights into context-specific factors influencing resistance trends.

Eq. (S5): Attributable Mortality Function for Antimicrobial Resistance

$$\mathbf{AM_{p,r} = \sum_{a,s,c} D_{p,r,a,s,c} \times (1 - RR_{p,s,a,s,c} / RR_{s,r,a,s,c})} \quad \text{(S5)}$$

where:
$\mathbf{AM_{p,r}}$ — Attributable mortality for pathogen p with resistance profile r.
$\sum_{a,s,c}$ — Summation over all combinations of age (a), sex (s), and clinical category (c).
$\mathbf{D_{p,r,a,s,c}}$ — Observed deaths for pathogen p, resistance profile r, stratified by age a, sex s, and clinical category c.
$\mathbf{RR_{p,s,a,s,c}}$ — Adjusted relative risk for resistant strains.
$\mathbf{RR_{s,r,a,s,c}}$ — Adjusted relative risk for susceptible strains.
$\mathbf{(1 - RR_p / RR_s)}$ — Proportion of excess mortality attributable to resistance.
Burden Estimation Note:
In addition to mortality estimates, Years of Life Lost (YLL) and Years Lived with Disability (YLD) were calculated to estimate Disability-Adjusted Life Years (DALYs). YLLs used age-specific life expectancy from Libya-specific World Bank tables, while YLDs were calculated by applying Global Burden of Disease (GBD) disability weights. This allowed for accurate, population-adjusted burden estimates, accounting for demographic heterogeneity and unequal healthcare access in Libya.

Supplementary Figures

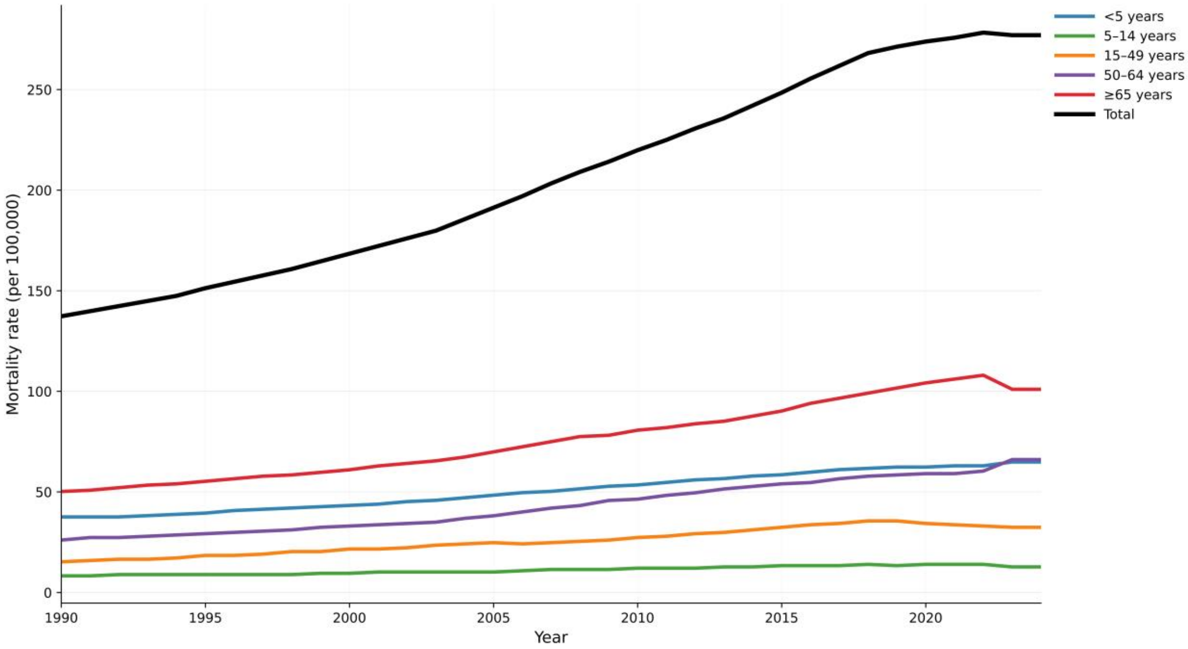

Figure S1: Age-specific AMR-attributable mortality rates in Libya (1990–2024). Time-series lines show age-specific AMR-attributable mortality rates (per 100,000 population) for <5 years, 5–14 years, 15–49 years, 50–64 years, and ≥65 years. The black line indicates the overall (all-ages) rate.

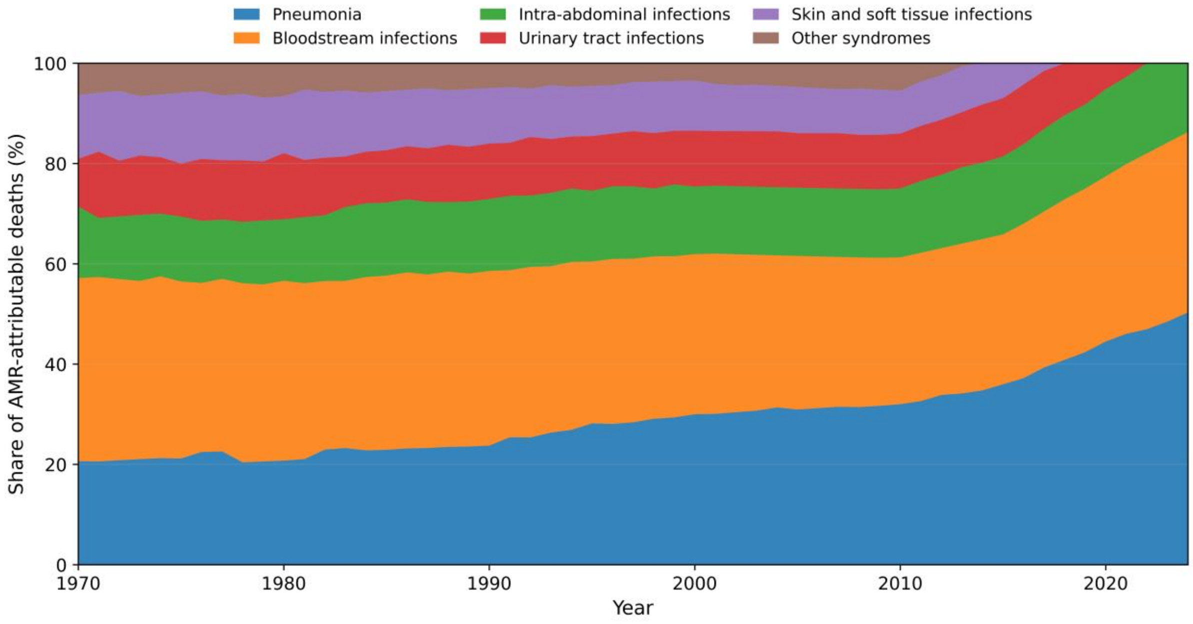

Figure S2: Proportional contribution of clinical syndromes to AMR-attributable deaths in Libya (1970–2024). A stacked area plot showing the year-specific percentage share of AMR-attributable deaths by syndrome (pneumonia, bloodstream infections, intra-abdominal infections, urinary tract infections, skin and soft tissue infections, and other syndromes.
